# Improved proteome coverage by using iTRAQ labelling and peptide OFFGEL fractionation

**DOI:** 10.1186/1477-5956-6-27

**Published:** 2008-10-13

**Authors:** Emilie Ernoult, Erick Gamelin, Catherine Guette

**Affiliations:** 1 Laboratory of Oncopharmacology-Pharmacogenetics, Centre INSERM Régional de Recherche sur le Cancer U892, Centre Régional de Lutte Contre le Cancer Paul Papin, Angers, France

## Abstract

**Background:**

The development of mass spectrometric techniques and fractionation methods now allows the investigation of very complex protein mixtures ranging from subcellular structures to tissues. Nevertheless, this work is particularly difficult due to the wide dynamic range of protein concentration in eukaryotic tissues. In this paper, we present a shotgun method whereby the peptides are fractionated using OFFGEL electrophoresis after iTRAQ labelling.

**Results:**

We demonstrated that iTRAQ peptide labelling enhances MALDI ionisation and that the OFFGEL fractionation of the labelled peptides introduces a supplementary criterion (pI) useful for validation and identification of proteins. We showed that iTRAQ samples allowed lower-concentrated proteins identification in comparison with free-labelled samples.

**Conclusion:**

The combined use of iTRAQ labelling and OFFGEL fractionation allows a considerable increase in proteome coverage of very complex samples prepared from total cell extracts and supports the low-concentrated protein identification.

## Background

The iTRAQ-reagent is well known for relative and absolute quantitation of proteins [1-3]. The interest of this multiplexing reagent is that 4 or 8 analysis samples [4] can be quantified simultaneously.

In this technique, the introduction of stable isotopes using iTRAQ reagents occurs on the level of proteolytic peptides. The iTRAQ technology uses an NHS ester derivative to modify primary amino groups by linking a mass balance group (carbonyl group) and a reporter group (based on N-methylpiperazine) to proteolytic peptides via the formation of an amide bond. Due to the isobaric mass design of the iTRAQ reagents, differentially-labelled peptides appear as a single peak in MS scans, reducing the probability of peak overlapping. When iTRAQ-tagged peptides are subjected to MS/MS analysis, the mass balancing carbonyl moiety is released as a neutral fragment, liberating the isotope-encoded reporter ions which provides relative quantitative information on proteins.

An inherent drawback of the reported iTRAQ technology is due to the enzymatic digestion of proteins prior to labelling, which artificially increases sample complexity. Since it has been shown that a reliable determination of protein dynamics requires quantitative evaluation of an adequate set of proteolytic peptides derived from each protein, the iTRAQ approach needs a powerful, multi-dimensional fractionation method of peptides before MS identification.

Reported peptide separation methods include strong cation exchange (SCX) chromatography and reverse-phase chromatography [5]. Recently, isoelectric focusing (IEF), a high-resolution electrophoresis technique for separation and concentration of amphoteric biomolecules at their isoelectric point (pI), has been used in shotgun proteomic experiments [6]. IEF runs in a buffer-free solution containing carrier ampholytes or in Immobilized pH gradient (IPG) gels. Recently, the use of IPG-IEF for the separation of complex peptide mixtures has been applied to the analysis of plasma and amniotic fluid [7,8] as well as to bacterial material [9]. However, a major limitation of this method is the tedious post-IEF sample processing. The IPG gel strip is divided into small sections for extraction and cleaning up of the peptides. A new concept called OFFGEL electrophoresis was recently introduced with the primary aim of purifying proteins and peptides [10]. This technique recovers the sample from the liquid phase and was demonstrated to be of great interest in shotgun proteomics [11]. IEF is not only a high resolution and high capacity separation method for peptides, it also provides additional physicochemical information like their isoelectric point [12,13]. The pI value provided is used as an independent validating and filtering tool during database search for MS/MS peptide sequence identification [14].

Recently, the compatibility of iTRAQ isotope labelling and OFFGEL-IEF for relative quantification and validation of sequence matches from database searching was shown from a BSA tryptic digest sample and complex eukaryotic samples [15,16] but surprisingly, no attempts was done to undertake comprehensive analysis of influence of iTRAQ labelling on the proteome coverage ratio.

In our work, we combined free-labelled peptides or iTRAQ labelled peptides and OFFGEL fractionation for the proteomic study of a very complex sample like the human neuroblastoma SH-SY5Y cell line [17-19] in a wide pI-range (pH 3–10) and compared the proteome coverage between free-samples and iTRAQ-samples.

## Results and discussion

### The influence of iTRAQ-reagent tagging

The 2D-LC separation of 200 μg of free-labelled digested proteins by SCX allowed identification of 159 proteins among which 116 proteins were characterised by at least 2 peptides (73% performance) (Table 1, NL-116). From four fractions of 50 μg of proteins which were reduced, blocked with MMTS, digested with trypsin, labelled using a different iTRAQ reagent, pooled and separated by SCX chromatography (iTRAQ-310), 472 proteins can be identified. 310 out of these have been identified using at least two peptides (Table 1). All the 116 proteins that were identified without labelling (NL-116) were also identified in the iTRAQ experiment. Nevertheless, iTRAQ labelling allowed 2.7 more proteins identified (by at least 2 peptides) compared to free-labelled experiments.

**Table 1:** The list of experiments and the number of identified proteins

Experiment	Quantity (μg)	Fractionation	Labelling	Total proteins	Proteins with 2 peptides (at least)	Yield (%)
NL-116	200	SCX	no	159	116	73

NL-184	200	OFFGEL-IEF	no	285	184	64

NL-235	200	SCX + OFFGEL-IEF	no	302	235	78

iTRAQ-310	200	SCX	iTRAQ	472	310	66

iTRAQ-429	400	SCX	iTRAQ	492	429	87

iTRAQ-739	400	OFFGEL-IEF	iTRAQ	879	739	84

All experiments: 1111 proteins, with 2 pept: 947 proteins

### Increase in peptide mass following iTRAQ labelling

The presence of iTRAQ label on the NH_2 _terminal adds a mass of 145 to the peptide *m/z*. If the peptide had a lysine residue at the Cterm position, two iTRAQ labels are added (290 Da). Looking at the list of peptides identified in the iTRAQ-310 label experiment, 61 unique peptides without lysine in their sequence had a *m/z *between 800 and 945, and 55 other peptides with a lysine at the Cterm extremity had a mass between 800 and 1090. These 116 peptides were not visible without iTRAQ labelling; nevertheless, they were found in the sequence of 60 proteins already identified by at least 2 peptides. Only 3 proteins were identified by a second peptide. The mass increase due to iTRAQ-labelling cannot be the unique explanation of the higher number of proteins identified nor the higher number of peptides contributing to the identification for each protein.

### iTRAQ increases ionisation of peptides containing lysines

**Figure 1:**
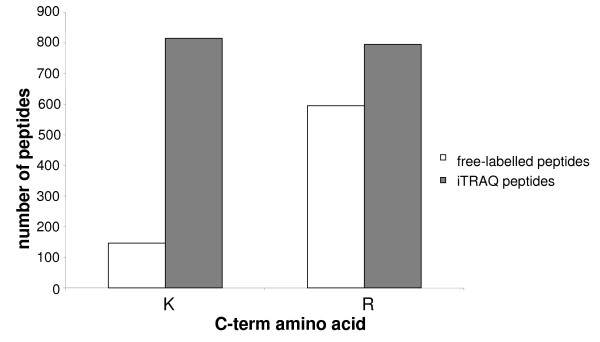
**iTRAQ labelling favours the peptide ionization**. Number of identified peptides with a lysine or an arginine in the C-term position; white bars: free-labelled peptides; black bars: iTRAQ peptides.

Analysis of the non-labelled peptide sequences showed that 20% of them had a lysine termination, and 80% an arginine termination (Figure 1). A recent study demonstrated that during MALDI ionisation, there is a linear correlation between peptide ionisation levels and the proton affinity of the amino acids, with the exception of lysine [20]. Arginine is the residue having the strongest proton affinity, and peptides containing this amino acid are those ionising the best. This conclusion is in line with our results; peptides containing arginine had better ionisation levels, therefore providing a better signal to noise ratio for simple MS, and thus providing higher quality MS/MS spectra responsible for more valid sequence identification than peptides having a Cterm lysine.

**Figure 2:**
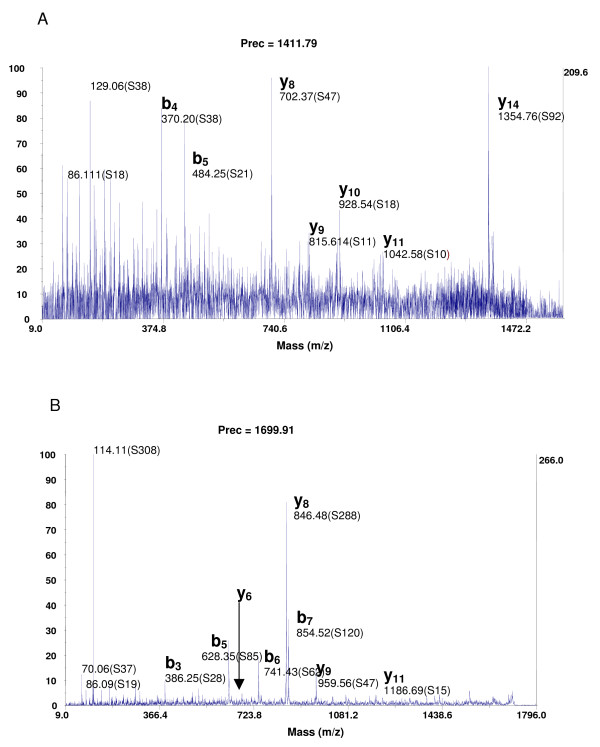
**MS/MS spectra of GALQNIIPASTGAAK (which allowed the identification of the Glyceraldehyde 3 phosphate deshydrogenase) from 200 μg of digested proteins fractionated by SCX chromatography**. (A) free-labelled experiment; (B) iTRAQ labelling experiment.

The analysis of peptides identified following iTRAQ labelling showed a balance of the number of arginine- or lysine-terminated peptides with 50% arginine-terminated and 50% lysine-terminated (Figure 1). Our results confirm those of Ross [1] who showed that in comparable whole-yeast experiments using electrospray, (much less sensitive to proton affinity than MALDI ionisation), the ratio of lysine to arginine-terminated peptides identified increased from 0.79 for native peptide, to 0.98 for iTRAQ derived peptides. The reporter group of the iTRAQ reagent is a piperazine group having 2 tertiary amines groups. By attaching these onto the lysine's lateral chain, a primary amine function is thereby replaced by two tertiary amine functions. We know that tertiary amines have a stronger proton affinity than the one primary amines (about 50 kJ/mole). By introducing 2 tertiary amine functions on peptides having lysines, their proton affinity and therefore their ionisation increase. For example, Figure 2 shows MS/MS spectra of the peptide GALQNIIPASTGAAK before and after iTRAQ labelling. The signal/noise ratio is multiplied by 6 for the most intense y_8 _ion. This result suggests that the proton affinity of iTRAQ modified lysines is almost equal to the one of arginines. We found that iTRAQ gave equivalent identification success rate for arginine- and lysine-terminated peptides, hence increasing the peptide coverage of proteins and increasing the chance of identifying low-concentration peptides.

### The influence of the quantity of iTRAQ-labelled peptides

From only 400 μg of reduced, blocked, digested cellular lysate labelled using iTRAQ, we were able after SCX separation to identify, 492 proteins, of which 429 with at least 2 peptides, giving an identification rate of 87% (iTRAQ-429). By doubling the amount of biological material to start with, identification rate for proteins with at least 2 peptides raised by 39% (Table 1).

### The influence of the fractioning method on free-labelled peptides

**Figure 3:**
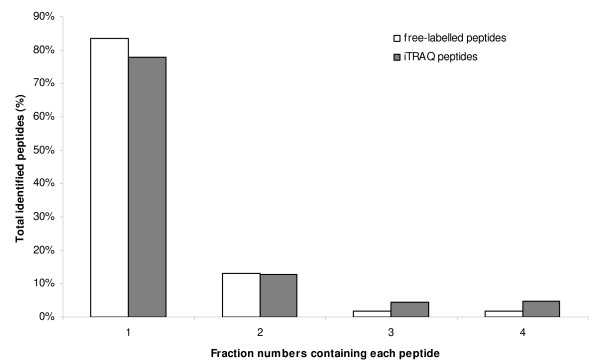
**Fractionwise distribution of identified SH-SY5Y peptides**. white bars: free-labelled peptides; black bars: iTRAQ peptides.

**Figure 4:**
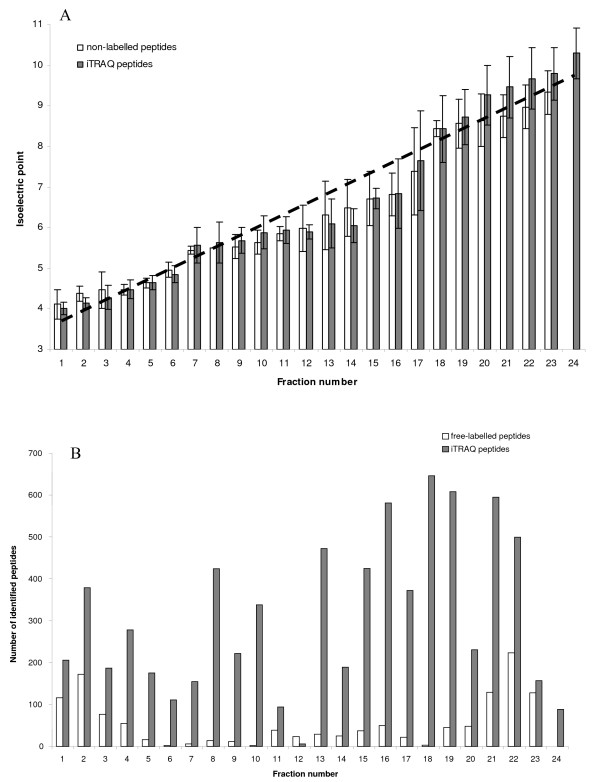
**Analysis of SH-SY5Y peptides pI after OFFGEL fractionation and MALDI- MS/MS identification**. (A) The average experimental pH of all peptides in a single fraction after filtering for false positive is presented as bars; white bars: non-labelled peptides; dark bars: iTRAQ peptides. Error bars indicate the SD of each fraction's experimental pI. The broken line is based on the theoretical pI values for an IPG strip of 24 cm ranging from pH 3–10; Agilent Technologies provided the theoretical pI values. (B) The total bar height shows the number of peptides in each fraction after filtering for false positive; white bars: non-labelled peptides; black bars: iTRAQ peptides.

The OFFGEL technology of 200 μg of free-labelled digested proteins allowed identification of 285 proteins, including 184 with at least 2 peptides (NL-184). By looking at the number of fractions in which each distinct peptide is found we can judge the fractionation quality of the technique. Figure 3 shows that 84% of the identified peptides are found in only one fraction and more than 95% are found in one or two fractions. This result is in agreement with the results found from earlier studies [21]. The average experimental pH value of each fraction is presented as a bar in Figure 4A. The theoretical pH values provided by the manufacturer were overlaid as a broken line. Average experimental pH values deviated from theoretical values by an average error of 0.29 pI unit. Only fractions close to neutrality (Fractions F12 to F18), and which do not contain a large number of peptides, showed an error of 0.38 pI unit in agreement with already published results [22]. Such an error was also found for the 3 more acidic fractions F1 to F3. For these 3 fractions, the error is largely due to the Bjellqvist algorithm [12] which is used in this study. This algorithm is known to overestimate pI values in the acidic range [22]. One feature of this experiment is that after screening with our previously-described criteria, no peptide was identified in fraction 24 (Figure 4B); this is in line with Fraterman's work [23]. In order to verify the identification of the peptides set and decrease false positive percentage after ProteinPilot calculations, we used the pI peptide property as an orthogonal property of these peptides of interest [24]. By separating peptides using OFFGEL, we showed that we can predict their pI with an accuracy of 0.4 pI unit. Under this condition, all peptides with unused ProteinPilot score > 1.3 and showing an experimental pI deviating by more than 0.4 pI unit from calculated value were excluded. All free-labelled peptides presented in this work were filtered according to their ProteinPilot unused score and pI as described above.

### The quality of OFFGEL separation of iTRAQ-labelled peptides

**Figure 5:**
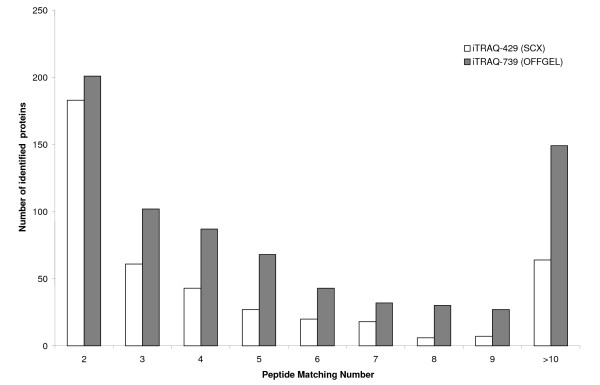
**The distribution of identified proteins based on the number of peptides used for protein identification**. Comparison between SCX and OFFGEL fractionation of iTRAQ samples.

By using OFFGEL separation by 24 fractions of 400 μg of iTRAQ-labelled peptides, we identified 879 proteins, of which 739 with at least 2 peptides (iTRAQ-739), giving an increase of 72% (Table 1) compared to SCX separation. OFFGEL experiment also increased the number of peptides identified per protein from 4.8 (iTRAQ-429) to 7.1 (iTRAQ-739), thereby increasing dramatically the confidence level of the protein identification. Figure 5 shows the quantity of proteins identified with different numbers of matching peptides with the two types of fractionation. We can see that OFFGEL gave a higher number of proteins identified in all fractions.

As shown in Figure 3, 78% of the identified peptides are found in only one fraction and about 90% are found in one or two fractions, confirming the distribution of free-labelled peptides in our work and the results from earlier studies [16,21].

The distribution of peptides per fraction is shown in Figure 4. The pI value for each identified peptide was calculated by using Bjellqvist's algorithm without taking into account the iTRAQ groups in N-term position and/or on the lateral lysine chain. Using these data, average pI values with standard deviations were calculated for all peptides identified in each fraction (Figure 4A). The average experimental pI value deviated from the theoretical pI value by an average error of +/- 0.43. In the pH range of 3 to 8, the average pI value of the labelled peptides fits very well with the average value of the non-labelled peptides. Major deviations were observed at the basic pH range (8.3–10.0) for fractions 18–24. By splitting OFFGEL fractionation into 2 zones, we noticed that the mean error in the pH 3–8 range was +/- 0.34 and was rising to +/- 0.64 in the more alkaline range (pH 8–10); this can probably be explained by the fact that the software used for calculating the pI value does not take into account the presence of the iTRAQ label. As for the free-labelled peptides, we introduced the pI iTRAQ-labelled peptides property as a filter to verify the identification of the set of peptides. For the fractions F1 to F17 (pH 3.3–8.0 range), all peptides with an unused ProteinPilot score above 1.3 and with an experimental pI difference larger than 0.4 pI unit were excluded. For the fractions F18-F24 (pH 8.3–10.0), we excluded all peptides with experimental pI difference greater than 0.7 pI unit. All iTRAQ-labelled peptides presented in this work were filtered using this protocol.

### Proteomic coverage

Combining results of all experiments, we end up with 947 proteins (Additional file1) identified with at least 2 peptides and 6119 unique peptides. 380 detected proteins have at least 5 or more unique peptides identified.

**Figure 6:**
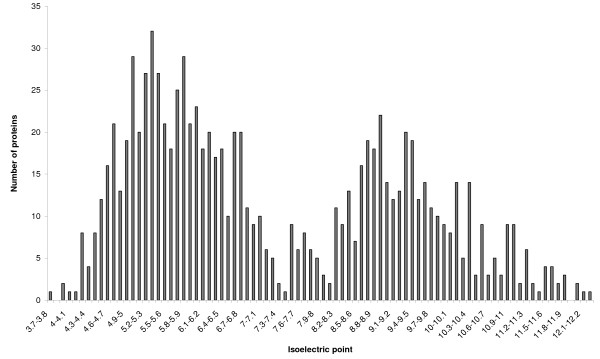
**Protein distribution in function of pI**. The isoelectric point distribution of SH-SY5Y proteins identified with 2 or more peptides.

**Figure 7:**
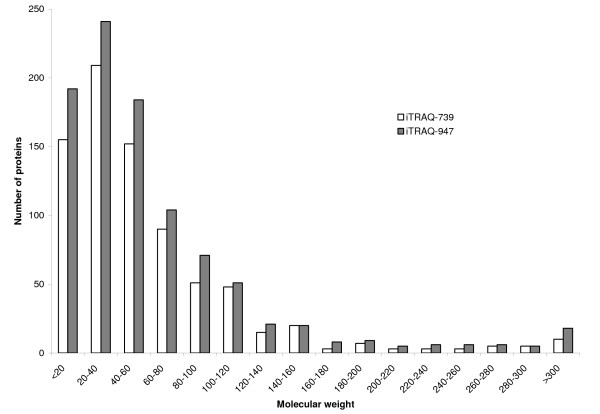
**Protein distribution in function of MW**. The molecular weight distribution of SH-SY5Y proteins identified with two or more peptides; white bars: from one experiement with 400 μg of digested proteins and labelled with iTRAQ reagent ; black bars: from all the experiments

The proteins observed in our experiments were mapped in relation with their MW and pI. The distribution of pI values over a range of 3–12 is shown in Figure 6. Similar to previous computational analyses [25,26] our results show that the distribution of the predicted pI values of our identified proteins is typical of eukaryotic cells, showing a kind of tri-modal distribution. The pI value distribution of our identified proteins shows the same profile as the distribution of human proteins identified from epithelial mammary cells (quite different from human proteome) [25]. Our results show a fairly good coverage for both cytosolic and membrane proteins. Indeed, the coverage is slightly biased towards the identification of cytosolic proteins (cluster at pI 6), presumably due to a better solubility of cytosolic proteins compared to membrane proteins (cluster at pI 9). Improved coverage of membrane proteins from plasma membrane and subcellular organelles can be achieved by coupling OFFGEL with further subcellular fractionation and better membrane-solubilising strategies. The molecular weight (MW) distribution of the identified proteins is shown in Figure 7. The overall trend of the MW distribution of the identified proteins versus the human proteome is very similar, even if proteins in the <20 kDa range is slightly higher suggesting there is no real bias in our protocol.

By comparing MW profiles of the proteins identified in the iTRAQ-947 and iTRAQ-739 experiments, we can see that the profiles are comparable (Figure 7), strengthening the hypothesis that the iTRAQ-739 experiment correctly reflects proteome coverage of the cell line SH-SY5Y.

### Relative abundance of proteins

**Figure 8:**
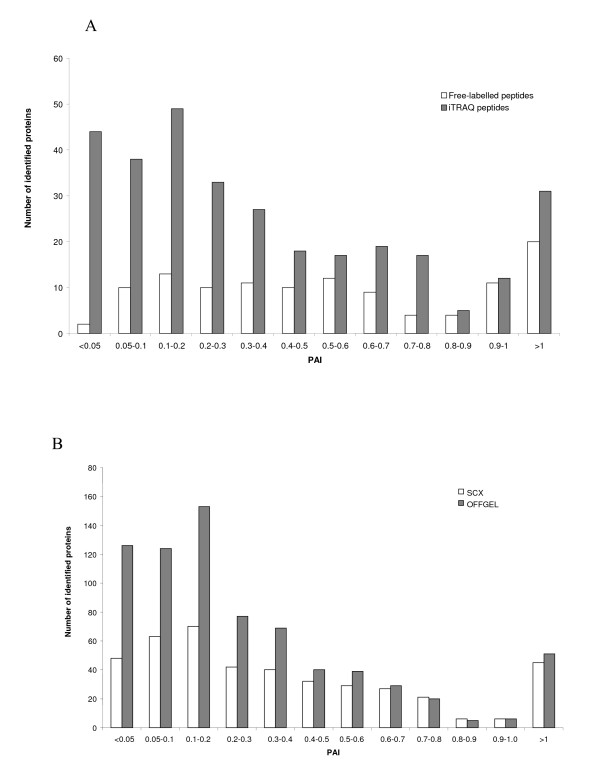
**Protein abundance index (PAI) for the identified proteins**. (A) influence of the iTRAQ labelling: free-labelled *vs *iTRAQ peptides (from 200 μg of digested proteins fractionated by SCX chromatography); (B) influence of the fractionation mode: SCX *vs *OFFGEL (from 400 μg of digested proteins and labelled with iTRAQ reagent).

One way to get an idea of the quantity of a protein in a complex mixture is to calculate its PAI [27], which represents the number of identified peptides divided by the number of theoretical tryptic peptides. The PAI mean value for the iTRAQ-947 experiment is 0.32; 50% of the proteins have a PAI value < 0.14 and 21% have a PAI value < 0.05, a characteristically value for proteins present at low concentration. Some of these low abundance proteins such as Transcription factor BTF3 homologue 4, Transcription initiation factor IIF sub-unit beta, and Nuclear factor 1 B-type are identified in this range of low PAI levels. The comparison between an experiment without labelling and an experiment with iTRAQ labelling, using similar method of separation, shows a higher number of proteins identified in each category for iTRAQ-labelled proteins (Figure 8A). This result is particularly convincing when looking at very low PAI values, and hence at proteins present at low concentration. In the area of PAI levels lower than 0.05, only 2 proteins were identified in the non-labelling experiment, compared to 44 identified following iTRAQ labelling. We carried out comparisons for similar PAI values for SCX-C18/2D-LC and OFFGEL-IEF fractionation. Starting with only 400 μg of proteins with iTRAQ labelling, we demonstrated that OFFGEL-IEF separation gives the largest number of identified proteins, particularly in areas with low PAI values (Figure 8B). By comparing the PAI values of proteins identified with at least 2 peptides in the different experiments, we can clearly see the superiority of iTRAQ labelling in comparison to non-labelled molecules, and the superiority of the OFFGEL-IEF fractionation method as compared to the SCX-C18/2D-LC method.

## Conclusion

iTRAQ is at evidence a very powerful tool, recognised for its ability to relatively quantify proteins. In this work, we showed that the iTRAQ reagent improves MALDI ionisation, especially for peptides containing lysine. A direct consequence of this property is the better chance of identifying low abundance proteins in complex biological materials. We were also able to demonstrate that an OFFGEL fractionation step, has a positive influence on the number of proteins identified compared to SCX method.

From standard clinical protein quantities (400 μg), we proposed a methodology allowing the identification of more than 800 proteins. Having established a protein database of SH-SY5Y cells, we can now use it as a reference for further research in this domain.

## Materials and methods

### Cell culture and protein extraction

The human neuroblastoma cell line SH-SY5Y was a gift from Dr F. Vallette (INSERM U601, Nantes, France). Cells were grown in RPMI-1640 medium (Lonza) supplemented with 10% (v/v) fetal bovine serum (Lonza) without antibiotics in a, 5% humid CO_2 _atmosphere at 37°C. Cells were scraped and washed 3 times with PBS (300 × g, 5 min.). Cell pellets were then lysed by a solution containing 7M urea, 2M thiourea, 4% (w/v) CHAPS at 4°C for 1 h using a rotary shaker. Lysis was achieved by sonication on ice (3 × 5s pulses), and the lysates were clarified by centrifugation at 14,000 × g at 4°C for 15 min.

### Protein digestion and peptide labelling with iTRAQ reagents

Protein samples were cleaned up by precipitation with 6 volumes of cold acetone at -20°C overnight followed by resuspension of pellets in 0.5M triethylammonium bicarbonate (TEAB) pH 8.5 (Sigma-Aldrich) and final centrifugation step at 14,000 × g at 4°C for 15 min. We quantified proteins from supernatant with the 2-D Quant Kit (GE Healthcare, München, Germany) before diluting the protein samples up to 5 mg/ml with TEAB buffer. We took 50 or 100 μg of proteins for further reduction, alkylation, digestion and iTRAQ labelling using iTRAQ Reagents Multiplex Kit (Applied Biosystems) according to manufacturer's protocol. Briefly, protein samples were reduced with 5 mM tris-(2-carboxyethyl)phosphine (TCEP) at 60°C for 1 h. and the cysteine-groups were blocked using a 10 mM methyl methanethiosulfonate (MMTS) solution at room temperature for 10 min. . The proteins were then digested by 10 μg of trypsin at 37°C for 16 h. Each peptide solution was labelled at room temperature for 1 h with one iTRAQ reagent vial (mass tag 114, 115, 116 or 117) previously reconstituted with 70 μl of ethanol. Samples of the same protein content, and labelled respectively with 114, 115, 116 and 117 iTRAQ reagents, were combined and labelling reaction stopped by evaporation in a Speed Vac to obtain a brown pellet.

### Peptide OFFGEL fractionation

For pI-based peptide separation, we used the 3100 OFFGEL Fractionator (Agilent Technologies, Böblingen, Germany) with a 24-well set-up. Prior to electrofocusing, samples were desalted onto a Sep-Pak C18 cartridge (Waters). For 24-well set-up, peptide samples were diluted to a final volume of respectively 3.6 mL using OFFGEL peptide sample solution. To start, the IPG gel strips of 24 cm-long (GE Healthcare, München, Germany) with a 3–10 linear pH range were rehydrated with the Peptide IPG Strip Rehydradation Solution according to the protocol of the manufacturer for 15 min. Then, 150 μL of sample was loaded in each well. Electrofocusing of the peptides is performed at 20°C and 50 μA until the 50 kVh level was reached. After focusing, the 24 peptide fractions were withdrawn and the wells rinsed with 200 μL of a solution of water/methanol/formic acid (49/50/1) after 15 min, the rinsing solutions were pooled with their corresponding peptide fraction. All fractions were evaporated by centrifugation under vacuum and maintained at -20°C. Just prior nano-LC, the fractions were resuspended in 20 μL of H_2_O with 0.1% (v/v) TFA.

### Capillary LC separation

The samples were separated on an Ultimate 3,000 nano-LC system (Dionex, Sunnyvale, USA) using a C18 column (PepMap100, 3 μm, 100A, 75 μm id × 15 cm, Dionex) at a flow rate of 300 nL/min. . Buffer A was 2% ACN in water with 0.05% TFA and buffer B was 80% ACN in water with 0.04% TFA.

Peptides were desalted for 3 min. using only buffer A on the precolumn, followed by a separation for 60 min. using the following gradient: 0 to 20% B in 10 min., 20% to 55% B in 45 min. and 55% to 100% B in 5 min. Chromatograms were recorded at the wavelength of 214 nm. Peptide fractions were collected using a Probot microfraction collector (Dionex).

For the SCX fractionation, we used a salt gradient steps: 20-μl injections of 5 mM, 10 mM, 25 nM, 50 mM, 75 mM,100 mM, 125 mM, 150 mM, 200 mM, 300 mM, 500 mM, 1000 mM NaCl

We used CHCA (LaserBioLabs, Sophia-Antipolis, France) as MALDI matrix. The matrix (concentration of 2 mg/mL in 70% ACN in water with 0.1% TFA) was continuously added to the column effluent via a micro "T" mixing piece at 1.2 μL/min flow rate. After 14 min run, a start signal was sent to the Probot to initiate fractionation. Fractions were collected for 10s and spotted on a MALDI sample plate (1,664 spots per plate, Applied Biosystems, Foster City, CA).

### MALDI-MS/MS

MS and MS/MS analyses of off-line spotted peptide samples were performed using the 4800 MALDI-TOF/TOF Analyser (Applied Biosystems). After screening of all LC-MALDI sample positions in MS-positive reflector mode using 1500 laser shots, the fragmentation of automatically-selected precursors was performed at collision energy of 1 kV using air as collision gas (pressure of ~2 × 10^-6 ^Torr). MS spectra were acquired between *m/z *800 and 4000. For internal calibration, we used the parent ion of Glu1-fibrinopeptide at *m/z *1570.677 diluted in the matrix (3 femtomoles per spot). Up to 12 of the most intense ion signals per spot position having a S/N > 12 were selected as precursors for MS/MS acquisition. Peptide and protein identification were performed by the ProteinPilot™ Software V 2.0 (Applied Biosystems) using the Paragon algorithm [28]. Each MS/MS spectrum was searched for Homo sapiens specie against the Uniprot/swissprot database (release 51 of October 2006). The searches were run using with the fixed modification of methylmethanethiosulfate labelled cysteine parameter enabled. Other parameters such as tryptic cleavage specificity, precursor ion mass accuracy and fragment ion mass accuracy are MALDI 4800 built-in functions of ProteinPilot software.

The ProteinPilot software calculates a confidence percentage (the unused score) which reflects the probability that the hit is a false positive, meaning that at 95% confidence level, there is a false positive identification chance of about 5%.

While this software automatically accepts all peptides having an identification confidence level >1%, only proteins having at least one peptide above 95% confidence were initially recorded. The low confidence peptides cannot give a positive protein identification by themselves, but may support the presence of a protein identified using other peptides with higher confidence. Performing the search against a concatenated database containing both forward and reversed sequences allowed estimation of the false discovery rate below1%.

The experimental pI for each peptide was calculated using a pI/Mw tool of the ExPASy Proteomic Server [12,29].

## Abbreviations

NHS: N-Hydrosuccinimide; SCX: strong cation exchange; IEF: isoelectric focusing; IPG: Immobilized pH gradient; MMTS: methyl methanethiosulfonate; TFA: trifluoroacetic acid; PAI: protein abundance indice; CHCA: α-cyano-*4*-hydroxy-cinnamic acid; ACN: acetonitrile.

## Competing interests

The authors declare that they have no competing interests.

## Authors' contributions

EE performed the laboratory experiments, carried out the LC separation of peptides samples and assisted in the writing of the final manuscript. EG supervised the project. CG carried out the MALDI-TOF/TOF analysis, participated in the design of the study and wrote the manuscript. CG supervised and coordinated the project. All authors read and approved the final manuscript.

## Supplementary Material

Additional file 1 **List of identified proteins with at least 2 peptides.** The proteins provided result from the combination of all the experiements. Click here for file
