# Supplementary material for: Improved proteome coverage by using iTRAQ labelling and peptide OFFGEL fractionation
Source: Proteome Sci. 2008 Oct 13;6:27. doi: 10.1186/1477-5956-6-27 (PMC2572582; doi:10.1186/1477-5956-6-27)
Supplement: Additional file 1 — List of identified proteins with at least 2 peptides. The proteins provided result from the combination of all the experiements. [file 1477-5956-6-27-S1.pdf]

PAI	Accession Number	Proteins
0,018518519	Q9NR96|TLR9_HUMAN	Toll-like receptor 9 precursor (CD289 antigen) - Homo sa
0,019230769	Q10567|AP1B1_HUMAN	AP-1 complex subunit beta-1 (Adapter-related protein co
0,014492754	Q7RTU9|STRC_HUMAN	Stereocilin precursor - Homo sapiens (Human)
0,045454545	P00387|NCB5R_HUMAN	NADH-cytochrome b5 reductase (EC 1.6.2.2) (B5R) (Dia
0,052631579	Q96AY3|FKB10_HUMAN	FK506-binding protein 10 precursor (EC 5.2.1.8) (Peptidy
0,058823529	P24539|AT5F1_HUMAN	ATP synthase B chain, mitochondrial precursor (EC 3.6.3
0,044444444	Q6NW34|CC017_HUMAN	Uncharacterized protein C3orf17 - Homo sapiens (Huma
0,034482759	O00635|TRI38_HUMAN	Tripartite motif-containing protein 38 (RING finger protein
0,041666667	P19320|VCAM1_HUMAN	Vascular cell adhesion protein 1 precursor (V-CAM 1) (C
0,060606061	Q13740|CD166_HUMAN	CD166 antigen precursor (Activated leukocyte-cell adhes
0,019607843	Q92539|LPIN2_HUMAN	Lipin-2 - Homo sapiens (Human)
0,025974026	P49790|NU153_HUMAN	Nuclear pore complex protein Nup153 (Nucleoporin Nup1
0,133333333	P02765|FETUA_HUMAN	Alpha-2-HS-glycoprotein precursor (Fetuin-A) (Alpha-2-Z
0,014084507	P56715|RP1_HUMAN	Oxygen-regulated protein 1 (Retinitis pigmentosa RP1 pr
0,0625	O60216|RAD21_HUMAN	Double-strand-break repair protein rad21 homolog (hHR2
0,022727273	P36871|PGM1_HUMAN	Phosphoglucomutase-1 (EC 5.4.2.2) (Glucose phosphom
0,02919708	Q14690|RRP5_HUMAN	RRP5 protein homolog (Programmed cell death protein 1
0,022727273	O60244|CRSP2_HUMAN	CRSP complex subunit 2 (Cofactor required for Sp1 trans
0,016949153	P25205|MCM3_HUMAN	DNA replication licensing factor MCM3 (DNA polymerase
0,052631579	P17812|PYRG1_HUMAN	CTP synthase 1 (EC 6.3.4.2) (UTP--ammonia ligase 1) (
0,006644518	Q5THJ4|VP13D_HUMAN	Vacuolar protein sorting-associated protein 13D - Homo s
0,035714286	Q8WY21|SORC1_HUMAN	VPS10 domain-containing receptor SorCS1 precursor (h
0,055555556	Q96E17|RAB3C_HUMAN	Ras-related protein Rab-3C - Homo sapiens (Human)
0,064516129	Q86UU5|GGN_HUMAN	Gametogenetin - Homo sapiens (Human)
0,05	Q00537|PCTK2_HUMAN	Serine/threonine-protein kinase PCTAIRE-2 (EC 2.7.11.2
0,153846154	O60884|DNJA2_HUMAN	DnaJ homolog subfamily A member 2 (HIRA-interacting
0,035714286	P23771|GATA3_HUMAN	Trans-acting T-cell-specific transcription factor GATA-3 (
0,032258065	P35249|RFC4_HUMAN	Replication factor C subunit 4 (Replication factor C 37 kD
0,031578947	Q13017|RHG05_HUMAN	Rho GTPase-activating protein 5 (p190-B) - Homo sapie
0,010752688	P49454|CENPF_HUMAN	Centromere protein F (Kinetochore protein CENP-F) (Mit
0,026315789	Q8WVV9|HNRLL_HUMAN	Heterogeneous nuclear ribonucleoprotein L-like (Stromal
0,019230769	Q9BQK8|LPIN3_HUMAN	Lipin-3 (Lipin 3-like) - Homo sapiens (Human)
0,038461538	Q9NR48|ASH1L_HUMAN	Probable histone-lysine N-methyltransferase ASH1L (EC
0,037037037	O60341|LSD1_HUMAN	Lysine-specific histone demethylase 1 (EC 1.-.-.-) (Flavin
0,004535147	Q96PK2|MACF4_HUMAN	Microtubule-actin cross-linking factor 1, isoform 4 - Homo
0,142857143	Q8N1F7|NUP93_HUMAN	Nuclear pore complex protein Nup93 (Nucleoporin Nup93
0,020408163	O60271|JIP4_HUMAN	C-jun-amino-terminal kinase-interacting protein 4 (JNK-in
0,095238095	P11766|ADHX_HUMAN	Alcohol dehydrogenase class 3 chi chain (EC 1.1.1.1) (A
0,025	P33260|CP2CI_HUMAN	Cytochrome P450 2C18 (EC 1.14.14.1) (CYPIIC18) (P45
0,011627907	O15438|MRP3_HUMAN	Canalicular multispecific organic anion transporter 2 (AT
0,024390244	O15371|IF37_HUMAN	Eukaryotic translation initiation factor 3 subunit 7 (eIF-3 z
0,013605442	P49327|FAS_HUMAN	Fatty acid synthase (EC 2.3.1.85) [Includes: [Acyl-carrier-
0,02739726	P53396|ACLY_HUMAN	ATP-citrate synthase (EC 2.3.3.8) (ATP-citrate (pro-S-)-ly
0,022222222	O14497|ARI1A_HUMAN	AT-rich interactive domain-containing protein 1A (ARID d
0,05	Q7Z4W1|DCXR_HUMAN	L-xylulose reductase (EC 1.1.1.10) (XR) (Dicarbonyl/L-xy
0,024691358	Q6P0N0|CV106_HUMAN	Uncharacterized potential DNA-binding protein C14orf10
0,005847953	Q99996|AKAP9_HUMAN	A-kinase anchor protein 9 (Protein kinase A-anchoring pr
0,071428571	Q99961|SH3G1_HUMAN	SH3-containing GRB2-like protein 1 (Endophilin-2) (Endo
0,115384615	P04004|VTNC_HUMAN	Vitronectin precursor (Serum-spreading factor) (S-protein
0,071428571	Q96BR1|SGK3_HUMAN	Serine/threonine-protein kinase Sgk3 (EC 2.7.11.1) (Ser
0,012345679	Q9HAV4|XPO5_HUMAN	Exportin-5 (Exp5) (Ran-binding protein 21) - Homo sapie
0,014084507	Q9Y2L1|RRP44_HUMAN	Exosome complex exonuclease RRP44 (EC 3.1.13.-) (Ri
0,025	P29144|TPP2_HUMAN	Tripeptidyl-peptidase 2 (EC 3.4.14.10) (Tripeptidyl-peptid
0,058823529	Q9H0U4|RAB1B_HUMAN	Ras-related protein Rab-1B - Homo sapiens (Human)
0,045454545	Q9NZ01|GPSN2_HUMAN	Synaptic glycoprotein SC2 - Homo sapiens (Human)

0,020833333	Q08426|ECHP_HUMAN	Peroxisomal bifunctional enzyme (PBE) (PBFE) [Includes
0,095238095	Q12926|ELAV2_HUMAN	ELAV-like protein 2 (Hu-antigen B) (HuB) (ELAV-like neu
0,03125	Q9Y5J1|UTP18_HUMAN	U3 small nucleolar RNA-associated protein 18 homolog (
0,114285714	O94833|BPAEA_HUMAN	Bullous pemphigoid antigen 1, isoforms 6/9/10 (Trabeculi
0,08	Q9HCU4|CELR2_HUMAN	Cadherin EGF LAG seven-pass G-type receptor 2 precur
0,114285714	Q9Y5H2|PCDGB_HUMAN	Protocadherin gamma A11 precursor (PCDH-gamma-A1
0,042253521	Q7Z5Q5|DPOLN_HUMAN	DNA polymerase nu (EC 2.7.7.7) - Homo sapiens (Huma
0,007532957	Q8WXH0|SYNE2_HUMAN	Nesprin-2 (Nuclear envelope spectrin repeat protein 2) (S
0,014492754	P22102|PUR2_HUMAN	Trifunctional purine biosynthetic protein adenosine-3 [Incl
0,005050505	P25054|APC_HUMAN	Adenomatous polyposis coli protein (Protein APC) - Hom
0,022222222	P11137|MAP2_HUMAN	Microtubule-associated protein 2 (MAP 2) (MAP-2) - Hom
0,037735849	P35606|COPB2_HUMAN	Coatomer subunit beta' (Beta'-coat protein) (Beta'-COP) (
0,055555556	O00487|PSDE_HUMAN	26S proteasome non-ATPase regulatory subunit 14 (26S
0,025210084	P42694|HELZ_HUMAN	Probable helicase with zinc-finger domain (EC 3.6.1.-) - H
0,008130081	P46940|IQGA1_HUMAN	Ras GTPase-activating-like protein IQGAP1 (p195) - Hon
0,008849558	Q9BV73|CP250_HUMAN	Centrosome-associated protein CEP250 (Centrosomal pr
0,076923077	P22695|UQCR2_HUMAN	Ubiquinol-cytochrome-c reductase complex core protein 2
0,030769231	O43719|HTSF1_HUMAN	HIV Tat-specific factor 1 (Tat-SF1) - Homo sapiens (Hum
0,030534351	Q8WYP5|AHTF1_HUMAN	AT-hook-containing transcription factor 1 (Embryonic larg
0,017857143	Q7LBC6|JHD2B_HUMAN	JmjC domain-containing histone demethylation protein 2E
0,022222222	Q9H0B6|KLC2_HUMAN	Kinesin light chain 2 (KLC 2) - Homo sapiens (Human)
0,043478261	P24941|CDK2_HUMAN	Cell division protein kinase 2 (EC 2.7.11.22) (p33 protein
0,037974684	P33176|KINH_HUMAN	Kinesin heavy chain (Ubiquitous kinesin heavy chain) (Uk
0,048780488	Q9HCC0|MCCC2_HUMAN	Methylcrotonoyl-CoA carboxylase beta chain, mitochondr
0,01863354	P13533|MYH6_HUMAN	Myosin heavy chain, cardiac muscle alpha isoform (MyH(
0,038461538	Q9Y5Z7|HCFC2_HUMAN	Host cell factor 2 (HCF-2) (C2 factor) - Homo sapiens (Hu
0,021052632	Q9UPQ9|TNC6B_HUMAN	Trinucleotide repeat-containing 6B protein - Homo sapien
0,006993007	Q9UPN3|MACF1_HUMAN	Microtubule-actin cross-linking factor 1, isoforms 1/2/3/5 (
0,038461538	Q9BRP4|WDR71_HUMAN	WD repeat protein 71 (Protein G-16) - Homo sapiens (Hu
0,024390244	P27708|PYR1_HUMAN	CAD protein [Includes: Glutamine-dependent carbamoyl-p
0,042857143	Q13308|PTK7_HUMAN	Tyrosine-protein kinase-like 7 precursor (Colon carcinoma
0,076923077	Q92558|WASF1_HUMAN	Wiskott-Aldrich syndrome protein family member 1 (WAS
0,09375	P02671|FIBA_HUMAN	Fibrinogen alpha chain precursor [Contains: Fibrinopeptic
0,036036036	Q7Z4S6|KI21A_HUMAN	Kinesin family member 21A (Kinesin-like protein KIF2) (R
0,045454545	Q9NQC3|RTN4_HUMAN	Reticulon-4 (Neurite outgrowth inhibitor) (Nogo protein) (F
0,142857143	Q16186|ADRM1_HUMAN	Adhesion-regulating molecule 1 precursor (110 kDa cell m
0,068965517	O00170|AIP_HUMAN	AH receptor-interacting protein (AIP) (Aryl-hydrocarbon re
0,016129032	Q8WWK9|CKAP2_HUMAN	Cytoskeleton-associated protein 2 (Tumor-associated mic
0,111111111	Q96FW1|OTUB1_HUMAN	Ubiquitin thioesterase protein OTUB1 (EC 3.4.-.-) (Otubai
0,066666667	P17252|KPCA_HUMAN	Protein kinase C alpha type (EC 2.7.11.13) (PKC-alpha) (
0,023809524	Q5JPB2|CT174_HUMAN	Uncharacterized protein C20orf174 - Homo sapiens (Hum
0,009090909	Q6WCQ1|MRIP_HUMAN	Myosin phosphatase Rho-interacting protein (Rho-interac
0,272727273	Q9BTT0|AN32E_HUMAN	Acidic leucine-rich nuclear phosphoprotein 32 family mem
0,035294118	O60841|IF2P_HUMAN	Eukaryotic translation initiation factor 5B (eIF-5B) (Transl
0,090909091	P62879|GBB2_HUMAN	Guanine nucleotide-binding protein G(I)/G(S)/G(T) subun
0,010204082	Q6PL18|ATAD2_HUMAN	ATPase family AAA domain-containing protein 2 - Homo
0,037735849	P48147|PPCE_HUMAN	Prolyl endopeptidase (EC 3.4.21.26) (Post-proline cleavir
0,03030303	Q96F86|EDC3_HUMAN	Enhancer of mRNA decapping protein 3 (YjeF domain-co
0,054054054	O95747|OXSR1_HUMAN	Serine/threonine-protein kinase OSR1 (EC 2.7.11.1) (Oxi
0,0234375	Q6TFL3|CI093_HUMAN	Uncharacterized protein C9orf93 - Homo sapiens (Humar
0,047619048	Q13563|PKD2_HUMAN	Polycystin-2 (Polycystic kidney disease 2 protein homolo
0,153846154	O75446|SAP30_HUMAN	Histone deacetylase complex subunit SAP30 (Sin3-assoc
0,083333333	Q9BYG3|MK67I_HUMAN	MKI67 FHA domain-interacting nucleolar phosphoprotein
0,028571429	Q96HC4|PDLI5_HUMAN	PDZ and LIM domain protein 5 (Enigma homolog) (Enigm
0,04	Q9Y570|PPME1_HUMAN	Protein phosphatase methylesterase 1 (EC 3.1.1.-) (PME
0,017964072	O75369|FLNB_HUMAN	Filamin-B (FLN-B) (Beta-filamin) (Actin-binding-like protei

0,025	Q5T7W7|CI097_HUMAN	Uncharacterized protein C9orf97 - Homo sapiens (Human
0,044444444	Q6NXG1|RB35A_HUMAN	RNA-binding protein 35A (RNA-binding motif protein 35A
0,046511628	Q9NTZ6|RBM12_HUMAN	RNA-binding protein 12 (RNA-binding motif protein 12) (S
0,038461538	Q92786|PROX1_HUMAN	Homeobox prospero-like protein PROX1 (PROX 1) - Hom
0,157894737	Q13319|CD5R2_HUMAN	Cyclin-dependent kinase 5 activator 2 precursor (CDK5 a
0,12195122	Q8TDI0|CHD5_HUMAN	Chromodomain helicase-DNA-binding protein 5 (EC 3.6.1
0,027777778	O00257|CBX4_HUMAN	E3 SUMO-protein ligase CBX4 (Chromobox protein homo
0,036363636	Q96QC0|PP1RA_HUMAN	Serine/threonine-protein phosphatase 1 regulatory subun
0,03125	O60664|M6PBP_HUMAN	Mannose-6-phosphate receptor-binding protein 1 (Cargo
0,043478261	P49588|SYAC_HUMAN	Alanyl-tRNA synthetase, cytoplasmic (EC 6.1.1.7) (Alanir
0,040816327	O75400|PRP40_HUMAN	Pre-mRNA-processing factor 40 homolog A (Formin-bind
0,010989011	P35251|RFC1_HUMAN	Replication factor C subunit 1 (Replication factor C large
0,007575758	Q02224|CENPE_HUMAN	Centromeric protein E (CENP-E) - Homo sapiens (Human
0,017241379	Q15042|RB3GP_HUMAN	Rab3 GTPase-activating protein catalytic subunit (RAB3
0,058823529	P09622|DLDH_HUMAN	Dihydrolipoyl dehydrogenase, mitochondrial precursor (E
0,035714286	P09001|RM03_HUMAN	Mitochondrial 39S ribosomal protein L3 (L3mt) (MRP-L3)
0,176470588	P55036|PSMD4_HUMAN	26S proteasome non-ATPase regulatory subunit 4 (26S p
0,111111111	O75390|CISY_HUMAN	Citrate synthase, mitochondrial precursor (EC 2.3.3.1) - H
0,058823529	Q16740|CLPP_HUMAN	Putative ATP-dependent Clp protease proteolytic subunit
0,037735849	Q06418|TYRO3_HUMAN	Tyrosine-protein kinase receptor TYRO3 precursor (EC 2
0,032258065	P40938|RFC3_HUMAN	Replication factor C subunit 3 (Replication factor C 38 kD
0,096774194	Q13177|PAK2_HUMAN	Serine/threonine-protein kinase PAK 2 (EC 2.7.11.1) (p21
0,041237113	P52701|MSH6_HUMAN	DNA mismatch repair protein MSH6 (MutS-alpha 160 kDa
0,023529412	Q9P1Y5|K1543_HUMAN	Protein KIAA1543 - Homo sapiens (Human)
0,043478261	O94760|DDAH1_HUMAN	NG,NG-dimethylarginine dimethylaminohydrolase 1 (EC 3
0,083333333	P69892|HBG2_HUMAN; P6	Hemoglobin subunit gamma-2 (Hemoglobin gamma-2 cha
0,03030303	P17980|PRS6A_HUMAN	26S protease regulatory subunit 6A (Proteasome 26S sul
0,008	P24928|RPB1_HUMAN	DNA-directed RNA polymerase II largest subunit (EC 2.7
0,039215686	P54136|SYRC_HUMAN	Arginyl-tRNA synthetase, cytoplasmic (EC 6.1.1.19) (Argi
0,045454545	P31939|PUR9_HUMAN	Bifunctional purine biosynthesis protein PURH [Includes:
0,142857143	Q04760|LGUL_HUMAN	Lactoylglutathione lyase (EC 4.4.1.5) (Methylglyoxalase)
0,018691589	Q9NTJ3|SMC4_HUMAN	Structural maintenance of chromosomes protein 4 (Chron
0,032608696	Q14203|DYNA_HUMAN	Dynactin-1 (150 kDa dynein-associated polypeptide) (DP
0,028571429	Q13409|DC1I2_HUMAN	Cytoplasmic dynein 1 intermediate chain 2 (Dynein interm
0,027777778	P48444|COPD_HUMAN	Coatomer subunit delta (Delta-coat protein) (Delta-COP)
0,092307692	P11586|C1TC_HUMAN	C-1-tetrahydrofolate synthase, cytoplasmic (C1-THF synt
0,026666667	Q9BXJ9|NARG1_HUMAN	NMDA receptor-regulated protein 1 (N-terminal acetyltran
0,040816327	O76094|SRP72_HUMAN	Signal recognition particle 72 kDa protein (SRP72) - Hom
0,142857143	Q9BYV8|CEP41_HUMAN	Centrosomal protein of 41 kDa (Protein Cep41) (Testis-sp
0,076923077	Q13347|IF32_HUMAN	Eukaryotic translation initiation factor 3 subunit 2 (eIF-3 b
0,045454545	Q15003|CND2_HUMAN	Condensin complex subunit 2 (Non-SMC condensin I com
0,049180328	Q99460|PSMD1_HUMAN	26S proteasome non-ATPase regulatory subunit 1 (26S p
0,088235294	Q13418|ILK_HUMAN	Integrin-linked protein kinase (EC 2.7.11.1) (ILK-1) (ILK-2
0,015503876	Q01484|ANK2_HUMAN	Ankyrin-2 (Brain ankyrin) (Ankyrin-B) (Ankyrin, nonerythro
0,060606061	P05091|ALDH2_HUMAN	Aldehyde dehydrogenase, mitochondrial precursor (EC 1
0,019230769	Q96SB3|NEB2_HUMAN	Neurabin-2 (Neurabin-II) (Spinophilin) (Protein phosphata
0,068965517	O43175|SERA_HUMAN	D-3-phosphoglycerate dehydrogenase (EC 1.1.1.95) (3-P
0,029761905	Q9Y490|TLN1_HUMAN	Talin-1 - Homo sapiens (Human)
0,083333333	P17568|NDUB7_HUMAN	NADH dehydrogenase [ubiquinone] 1 beta subcomplex s
0,086956522	P62714|PP2AB_HUMAN	Serine/threonine-protein phosphatase 2A catalytic subuni
0,029411765	P53618|COPB_HUMAN	Coatomer subunit beta (Beta-coat protein) (Beta-COP) - I
0,058823529	Q15437|SC23B_HUMAN	Protein transport protein Sec23B (SEC23-related protein
0,15	P12004|PCNA_HUMAN	Proliferating cell nuclear antigen (PCNA) (Cyclin) - Homo
0,066666667	O43708|MAAI_HUMAN	Maleylacetoacetate isomerase (EC 5.2.1.2) (MAAI) (Gluta
0,025316456	Q6Y7W6|PERQ2_HUMAN	PERQ amino acid-rich with GYF domain-containing prote
0,055555556	Q5JWF2|GNAS1_HUMAN	Guanine nucleotide-binding protein G(s) subunit alpha iso

0,051724138	P05023|AT1A1_HUMAN	Sodium/potassium-transporting ATPase alpha-1 chain pr
0,066666667	P08758|ANXA5_HUMAN	Annexin A5 (Annexin V) (Lipocortin V) (Endonexin II) (Ca
0,035087719	P13637|AT1A3_HUMAN	Sodium/potassium-transporting ATPase alpha-3 chain (E
0,142857143	Q9NS69|TOM22_HUMAN	Mitochondrial import receptor subunit TOM22 homolog (T
0,048387097	Q02218|ODO1_HUMAN	2-oxoglutarate dehydrogenase E1 component, mitochonc
0,042553191	P31040|DHSA_HUMAN	Succinate dehydrogenase [ubiquinone] flavoprotein subu
0,037735849	Q07065|CKAP4_HUMAN	Cytoskeleton-associated protein 4 (63 kDa membrane pr
0,054545455	Q9UKI8|TLK1_HUMAN	Serine/threonine-protein kinase tousled-like 1 (EC 2.7.11
0,102040816	Q14974|IMB1_HUMAN	Importin beta-1 subunit (Karyopherin beta-1 subunit) (Nu
0,222222222	P35080|PROF2_HUMAN	Profilin-2 (Profilin II) - Homo sapiens (Human)
0,005773672	Q8WZ42|TITIN_HUMAN	Titin (EC 2.7.11.1) (Connectin) (Rhabdomyosarcoma anti
0,166666667	O43670|ZN207_HUMAN	Zinc finger protein 207 - Homo sapiens (Human)
0,039215686	Q8N442|GUF1_HUMAN	GTP-binding protein GUF1 homolog - Homo sapiens (Hu
0,026578073	P78527|PRKDC_HUMAN	DNA-dependent protein kinase catalytic subunit (EC 2.7.[illegible]
0,051282051	P17029|ZKSC1_HUMAN	Zinc finger with KRAB and SCAN domain-containing prot
0,049180328	Q99459|CDC5L_HUMAN	Cell division cycle 5-like protein (Cdc5-like protein) (Pom
0,05	P13798|ACPH_HUMAN	Acylamino-acid-releasing enzyme (EC 3.4.19.1) (AARE)
0,142857143	P27449|VATL_HUMAN	Vacuolar ATP synthase 16 kDa proteolipid subunit (EC 3
0,153846154	P67870|CSK2B_HUMAN	Casein kinase II subunit beta (CK II beta) (Phosvitin) (G5
0,08	P08397|HEM3_HUMAN	Porphobilinogen deaminase (EC 2.5.1.61) (Hydroxymeth
0,037037037	Q8N684|CPSF7_HUMAN	Cleavage and polyadenylation specificity factor 7 (Cleava
0,083333333	Q9Y3F4|STRAP_HUMAN	Serine-threonine kinase receptor-associated protein (UN
0,068965517	P41250|SYG_HUMAN	Glycyl-tRNA synthetase (EC 6.1.1.14) (Glycine--tRNA lig
0,035087719	Q13200|PSMD2_HUMAN	26S proteasome non-ATPase regulatory subunit 2 (26S
0,039215686	P08237|K6PF_HUMAN	6-phosphofructokinase, muscle type (EC 2.7.1.11) (Phos
0,235294118	Q15428|SF3A2_HUMAN	Splicing factor 3A subunit 2 (Spliceosome-associated pro
0,15	P30040|ERP29_HUMAN	Endoplasmic reticulum protein ERp29 precursor (ERp31)
0,142857143	P82979|HCC1_HUMAN	Nuclear protein Hcc-1 (Proliferation-associated cytokine-i
0,04	O00712|NFIB_HUMAN	Nuclear factor 1 B-type (Nuclear factor 1/B) (NF1-B) (NFI
0,086956522	P47756|CAPZB_HUMAN	F-actin capping protein subunit beta (CapZ beta) - Homo
0,142857143	Q9Y5J9|TIM8B_HUMAN	Mitochondrial import inner membrane translocase subuni
0,042553191	Q96PZ0|PUS7_HUMAN	Pseudouridylate synthase 7 homolog (EC 5.4.99.-) - Hom
0,054545455	P13010|KU86_HUMAN	ATP-dependent DNA helicase 2 subunit 2 (EC 3.6.1.-) (A
0,037037037	Q8NI36|WDR36_HUMAN	WD repeat protein 36 (T-cell activation WD repeat-contai
0,021276596	O60306|AQR_HUMAN	Intron-binding protein aquarius (Intron-binding protein of [illegible]
0,011235955	P15924|DESP_HUMAN	Desmoplakin (DP) (250/210 kDa paraneoplastic pemphig
0,03030303	Q8N163|K1967_HUMAN	Protein KIAA1967 (Deleted in breast cancer gene 1 prote
0,022727273	Q9HAU0|PKHA5_HUMAN	Pleckstrin homology domain-containing family A member
0,03030303	P46100|ATRX_HUMAN	Transcriptional regulator ATRX (EC 3.6.1.-) (ATP-depenc
0,071428571	Q96L94|SNX22_HUMAN	Sorting nexin-22 - Homo sapiens (Human)
0,054054054	P27338|AOFB_HUMAN	Amine oxidase [flavin-containing] B (EC 1.4.3.4) (Monoar
0,035087719	P55884|IF39_HUMAN	Eukaryotic translation initiation factor 3 subunit 9 (eIF-3 e
0,081967213	Q15029|U5S1_HUMAN	116 kDa U5 small nuclear ribonucleoprotein component (
0,038461538	P17858|K6PL_HUMAN	6-phosphofructokinase, liver type (EC 2.7.1.11) (Phosphc
0,083333333	P20042|IF2B_HUMAN	Eukaryotic translation initiation factor 2 subunit 2 (Eukary
0,127272727	P13591|NCA11_HUMAN	Neural cell adhesion molecule 1, 140 kDa isoform precur
0,181818182	P61966|AP1S1_HUMAN	AP-1 complex subunit sigma-1A (Adapter-related protein
0,041666667	P04843|RIB1_HUMAN	Dolichyl-diphosphooligosaccharide--protein glycosyltrans
0,040816327	Q01813|K6PP_HUMAN	6-phosphofructokinase type C (EC 2.7.1.11) (Phosphofru
0,022988506	Q6P2Q9|PRP8_HUMAN	Pre-mRNA-processing-splicing factor 8 (Splicing factor P
0,06	P13521|SCG2_HUMAN	Secretogranin-2 precursor (Secretogranin II) (SgII) (Chro
0,064516129	Q92598|HS105_HUMAN	Heat-shock protein 105 kDa (Heat shock 110 kDa protein
0,142857143	P55786|PSA_HUMAN	Puromycin-sensitive aminopeptidase (EC 3.4.11.-) (PSA)
0,133333333	Q8NBS9|TXND5_HUMAN	Thioredoxin domain-containing protein 5 precursor (Thior
0,024539877	P21333|FLNA_HUMAN	Filamin-A (Alpha-filamin) (Filamin-1) (Endothelial actin-bi
0,0625	P61086|UBC1_HUMAN	Ubiquitin-conjugating enzyme E2-25 kDa (EC 6.3.2.19) (

0,076923077	P29084|T2EB_HUMAN	Transcription initiation factor IIE subunit beta (TFIIE-beta)
0,065217391	Q14684|K0179_HUMAN	Protein KIAA0179 - Homo sapiens (Human)
0,023809524	O94906|PRP6_HUMAN	Pre-mRNA-processing factor 6 (PRP6 homolog) (U5 snR
0,028985507	Q96RY5|CRML_HUMAN	Protein cramped-like (Hematological and neurological ex
0,038461538	Q8WWM7|ATX2L_HUMAN	Ataxin-2-like protein (Ataxin-2 domain protein) (Ataxin-2-r
0,049180328	Q13330|MTA1_HUMAN	Metastasis-associated protein MTA1 - Homo sapiens (Hu
0,04	Q96DV4|RM38_HUMAN	39S ribosomal protein L38, mitochondrial precursor (L38r
0,083333333	Q96DI7|WDR57_HUMAN	WD repeat protein 57 (Prp8-binding protein) (hPRP8BP)
0,004796163	Q15149|PLEC1_HUMAN	Plectin-1 (PLTN) (PCN) (Hemidesmosomal protein 1) (HD
0,066666667	O43602|DCX_HUMAN	Neuronal migration protein doublecortin (Lissencephalin-)
0,2	P07858|CATB_HUMAN	Cathepsin B precursor (EC 3.4.22.1) (Cathepsin B1) (APP
0,030534351	Q92878|RAD50_HUMAN	DNA repair protein RAD50 (EC 3.6.-.-) (hRAD50) - Homo
0,033557047	O75643|U520_HUMAN	U5 small nuclear ribonucleoprotein 200 kDa helicase (EC
0,040983607	Q02880|TOP2B_HUMAN	DNA topoisomerase 2-beta (EC 5.99.1.3) (DNA topoisom
0,028571429	O43237|DC1L2_HUMAN	Cytoplasmic dynein 1 light intermediate chain 2 (Dynein li
0,044444444	Q5TZA2|CROCC_HUMAN	Rootletin (Ciliary rootlet coiled-coil protein) - Homo sapien
0,036036036	Q00341|VIGLN_HUMAN	Vigilin (High density lipoprotein-binding protein) (HDL-bin
0,030456853	Q01082|SPTB2_HUMAN	Spectrin beta chain, brain 1 (Spectrin, non-erythroid beta
0,066666667	P30520|PURA2_HUMAN	Adenylosuccinate synthetase isozyme 2 (EC 6.3.4.4) (Ad
0,08974359	P27816|MAP4_HUMAN	Microtubule-associated protein 4 (MAP 4) - Homo sapien
0,081632653	P56192|SYMC_HUMAN	Methionyl-tRNA synthetase, cytoplasmic (EC 6.1.1.10) (M
0,1	Q9Y333|LSM2_HUMAN	U6 snRNA-associated Sm-like protein LSm2 (snRNP core
0,055555556	Q9H6S0|YTDC2_HUMAN	YTH domain-containing protein 2 - Homo sapiens (Huma
0,038961039	Q6ZNG1|ZN600_HUMAN	Zinc finger protein 600 - Homo sapiens (Human)
0,03125	P22570|ADRO_HUMAN	NADPH:adrenodoxin oxidoreductase, mitochondrial precu
0,038461538	P17028|ZNF24_HUMAN	Zinc finger protein 24 (Zinc finger protein 191) (Zinc finge
0,117647059	P51148|RAB5C_HUMAN	Ras-related protein Rab-5C (RAB5L) (L1880) - Homo sap
0,034883721	Q96ST3|SIN3A_HUMAN	Paired amphipathic helix protein Sin3a (Transcriptional co
0,052631579	Q9Y285|SYFA_HUMAN	Phenylalanyl-tRNA synthetase alpha chain (EC 6.1.1.20)
0,073170732	P49591|SYSC_HUMAN	Seryl-tRNA synthetase, cytoplasmic (EC 6.1.1.11) (Serine
0,058823529	Q13427|PPIG_HUMAN	Peptidyl-prolyl cis-trans isomerase G (EC 5.2.1.8) (Peptic
0,033898305	Q12931|TRAP1_HUMAN	Heat shock protein 75 kDa, mitochondrial precursor (HSP
0,051724138	Q13111|CAF1A_HUMAN	Chromatin assembly factor 1 subunit A (CAF-1 subunit A)
0,052631579	P00367|DHE3_HUMAN	Glutamate dehydrogenase 1, mitochondrial precursor (EC
0,055555556	O14910|LIN7A_HUMAN	Lin-7 homolog A (Lin-7A) (hLin-7) (Mammalian lin-seven
0,008	P26358|DNMT1_HUMAN	DNA (cytosine-5)-methyltransferase 1 (EC 2.1.1.37) (Dnm
0,15	P46109|CRKL_HUMAN	Crk-like protein - Homo sapiens (Human)
0,083333333	Q13557|KCC2D_HUMAN	Calcium/calmodulin-dependent protein kinase type II delta
0,079365079	O75534|CSDE1_HUMAN	Cold shock domain-containing protein E1 (UNR protein) (
0,073170732	P50991|TCPD_HUMAN	T-complex protein 1 subunit delta (TCP-1-delta) (CCT-de
0,111111111	Q86U06|RBM23_HUMAN	Probable RNA-binding protein 23 (RNA-binding motif prot
0,090909091	Q99832|TCPH_HUMAN	T-complex protein 1 subunit eta (TCP-1-eta) (CCT-eta) (H
0,055555556	P54578|UBP14_HUMAN	Ubiquitin carboxyl-terminal hydrolase 14 (EC 3.1.2.15) (U
0,036585366	Q6PKG0|LARP1_HUMAN	La-related protein 1 (La ribonucleoprotein domain family r
0,076923077	P40939|ECHA_HUMAN	Trifunctional enzyme subunit alpha, mitochondrial precurs
0,147058824	Q01518|CAP1_HUMAN	Adenylyl cyclase-associated protein 1 (CAP 1) - Homo sa
0,03125	Q07960|RHG01_HUMAN	Rho GTPase-activating protein 1 (GTPase-activating prot
0,333333333	P63000|RAC1_HUMAN	Ras-related C3 botulinum toxin substrate 1 precursor (p2
0,051282051	O60832|DKC1_HUMAN	H/ACA ribonucleoprotein complex subunit 4 (EC 5.4.99.-)
0,078947368	Q02790|FKBP4_HUMAN	FK506-binding protein 4 (EC 5.2.1.8) (Peptidyl-prolyl cis-t
0,075	O43776|SYNC_HUMAN	Asparaginyl-tRNA synthetase, cytoplasmic (EC 6.1.1.22)
0,048780488	Q96ME7|ZN512_HUMAN	Zinc finger protein 512 - Homo sapiens (Human)
0,058823529	P10636|TAU_HUMAN	Microtubule-associated protein tau (Neurofibrillary tangle
0,090909091	P07954|FUMH_HUMAN	Fumarate hydratase, mitochondrial precursor (EC 4.2.1.2
0,016528926	Q96L96|ALPK3_HUMAN	Alpha-protein kinase 3 (EC 2.7.11.-) (Muscle alpha-protei
0,375	P26885|FKBP2_HUMAN	FK506-binding protein 2 precursor (EC 5.2.1.8) (Peptidyl-

0,028409091	Q14204|DYHC_HUMAN	Dynein heavy chain, cytosolic (DYHC) (Cytoplasmic dyne
0,166666667	Q9NRX2|RM17_HUMAN	39S ribosomal protein L17, mitochondrial precursor (L17r
0,044776119	P08133|ANXA6_HUMAN	Annexin A6 (Annexin VI) (Lipocortin VI) (P68) (P70) (Prot
0,19047619	Q92733|PRCC_HUMAN	Proline-rich protein PRCC (Papillary renal cell carcinoma
0,113207547	P45974|UBP5_HUMAN	Ubiquitin carboxyl-terminal hydrolase 5 (EC 3.1.2.15) (Ub
0,046511628	P11413|G6PD_HUMAN	Glucose-6-phosphate 1-dehydrogenase (EC 1.1.1.49) (G
0,058823529	Q9H3P7|GCP60_HUMAN	Golgi resident protein GCP60 (Acyl-CoA-binding domain-
0,048780488	Q9UIG0|BAZ1B_HUMAN	Bromodomain adjacent to zinc finger domain protein 1B (
0,12	Q13148|TADBP_HUMAN	TAR DNA-binding protein 43 (TDP-43) - Homo sapiens (I
0,133333333	P82663|RT25_HUMAN	Mitochondrial 28S ribosomal protein S25 (S25mt) (MRP-S
0,043478261	Q06787|FMR1_HUMAN	Fragile X mental retardation 1 protein (Protein FMR-1) (F
0,038961039	Q9HCS7|XAB2_HUMAN	XPA-binding protein 2 (HCNP protein) - Homo sapiens (H
0,03030303	Q8WUM4|PDC6I_HUMAN	Programmed cell death 6-interacting protein (PDCD6-inte
0,021505376	P08572|CO4A2_HUMAN	Collagen alpha-2(IV) chain precursor - Homo sapiens (Hu
0,054545455	Q9BZE4|NOG1_HUMAN	Nucleolar GTP-binding protein 1 (Chronic renal failure ge
0,071428571	P33993|MCM7_HUMAN	DNA replication licensing factor MCM7 (CDC47 homolog
0,133333333	P06730|IF4E_HUMAN	Eukaryotic translation initiation factor 4E (eIF4E) (eIF-4E)
0,063063063	P07814|SYEP_HUMAN	Bifunctional aminoacyl-tRNA synthetase [Includes: Glutar
0,046511628	P51787|KCNQ1_HUMAN	Potassium voltage-gated channel subfamily KQT membe
0,111111111	Q13541|4EBP1_HUMAN	Eukaryotic translation initiation factor 4E-binding protein 1
0,086956522	O75533|SF3B1_HUMAN	Splicing factor 3B subunit 1 (Spliceosome-associated pro
0,125	Q06323|PSME1_HUMAN	Proteasome activator complex subunit 1 (Proteasome ac
0,052631579	P13984|T2FB_HUMAN	Transcription initiation factor IIF subunit beta (EC 3.6.1.-)
0,034782609	Q9UQE7|SMC3_HUMAN	Structural maintenance of chromosomes protein 3 (Chon
0,023255814	Q07866|KLC1_HUMAN	Kinesin light chain 1 (KLC 1) - Homo sapiens (Human)
0,068493151	Q9Y4L1|OXRP_HUMAN	150 kDa oxygen-regulated protein precursor (Orp150) (H
0,130434783	Q9BQ04|RBM4B_HUMAN	RNA-binding protein 4B (RNA-binding motif protein 4B) (I
0,235294118	Q03701|CEBPZ_HUMAN	CCAAT/enhancer-binding protein zeta (CCAAT-box-bindi
0,03	Q9UDY2|ZO2_HUMAN	Tight junction protein ZO-2 (Zonula occludens 2 protein) (
0,083333333	Q9UBQ0|VPS29_HUMAN	Vacuolar protein sorting-associated protein 29 (Vesicle pr
0,0625	Q8TAQ2|SMRC2_HUMAN	SWI/SNF-related matrix-associated actin-dependent regu
0,166666667	P51572|BAP31_HUMAN	B-cell receptor-associated protein 31 (BCR-associated pr
0,075	P17987|TCPA_HUMAN	T-complex protein 1 subunit alpha (TCP-1-alpha) (CCT-a
0,098039216	Q8NE71|ABCF1_HUMAN	ATP-binding cassette sub-family F member 1 (ATP-bindir
0,192307692	P23526|SAHH_HUMAN	Adenosylhomocysteinase (EC 3.3.1.1) (S-adenosyl-L-hor
0,121212121	Q92769|HDAC2_HUMAN	Histone deacetylase 2 (HD2) - Homo sapiens (Human)
0,1	P68036|UB2L3_HUMAN	Ubiquitin-conjugating enzyme E2 L3 (EC 6.3.2.19) (Ubiqu
0,028169014	O15164|TIF1A_HUMAN	Transcription intermediary factor 1-alpha (TIF1-alpha) (Tr
0,048780488	Q14247|SRC8_HUMAN	Src substrate cortactin (Amplaxin) (Oncogene EMS1) - H
0,125	O60888|CUTA_HUMAN	Protein CutA precursor (Brain acetylcholinesterase putati
0,043478261	Q86UP2|KTN1_HUMAN	Kinectin (Kinesin receptor) (CG-1 antigen) - Homo sapier
0,142857143	O95881|TXD12_HUMAN	Thioredoxin domain-containing protein 12 precursor (EC
0,057142857	Q6STE5|SMRD3_HUMAN	SWI/SNF-related matrix-associated actin-dependent regu
0,104166667	Q9UBT2|SAE2_HUMAN	SUMO-activating enzyme subunit 2 (EC 6.3.2.-) (Ubiquitir
0,153846154	P61081|UBC12_HUMAN	NEDD8-conjugating enzyme Ubc12 (EC 6.3.2.-) (Ubiquiti
0,055555556	P50416|CPT1A_HUMAN	Carnitine O-palmitoyltransferase I, liver isoform (EC 2.3.1
0,130434783	Q9BWF3|RBM4_HUMAN	RNA-binding protein 4 (RNA-binding motif protein 4) (RN/
0,058823529	Q15008|PSMD6_HUMAN	26S proteasome non-ATPase regulatory subunit 6 (26S p
0,074626866	P33991|MCM4_HUMAN	DNA replication licensing factor MCM4 (CDC21 homolog
0,081632653	Q13423|NNTM_HUMAN	NAD(P) transhydrogenase, mitochondrial precursor (EC 1
0,022727273	P48634|BAT2_HUMAN	Large proline-rich protein BAT2 (HLA-B-associated trans
0,071428571	P02751|FINC_HUMAN	Fibronectin precursor (FN) (Cold-insoluble globulin) (CIG
0,333333333	Q9H4B7|TBB1_HUMAN	Tubulin beta-1 chain - Homo sapiens (Human)
0,147058824	Q14157|UBP2L_HUMAN	Ubiquitin-associated protein 2-like (Protein NICE-4) - Hor
0,1	Q9ULV4|COR1C_HUMAN	Coronin-1C (Coronin-3) (hCRNN4) - Homo sapiens (Hum
0,083333333	Q9Y657|SPIN1_HUMAN	Spindlin-1 (Ovarian cancer-related protein) - Homo sapie

0,045977011	O60282|KIF5C_HUMAN	Kinesin heavy chain isoform 5C (Kinesin heavy chain neu
0,051948052	O15067|PUR4_HUMAN	Phosphoribosylformylglycinamidine synthase (EC 6.3.5.3
0,102564103	P22307|NLTP_HUMAN	Nonspecific lipid-transfer protein (EC 2.3.1.176) (Propano
0,153846154	P41223|BUD31_HUMAN	Protein BUD31 homolog (Protein G10 homolog) (EDG-2)
0,076923077	O95169|NDUB8_HUMAN	NADH dehydrogenase [ubiquinone] 1 beta subcomplex s
0,045454545	P45880|VDAC2_HUMAN	Voltage-dependent anion-selective channel protein 2 (VD
0,042857143	Q9H2U1|DHX36_HUMAN	Probable ATP-dependent RNA helicase DHX36 (EC 3.6.1
0,181818182	Q13838|UAP56_HUMAN	Spliceosome RNA helicase BAT1 (EC 3.6.1.-) (DEAD bo
0,09375	P02675|FIBB_HUMAN	Fibrinogen beta chain precursor [Contains: Fibrinopeptide
0,277777778	Q9UMX0|UBQL1_HUMAN	Ubiquilin-1 (Protein linking IAP with cytoskeleton 1) (PLIC
0,05	P60880|SNP25_HUMAN	Synaptosomal-associated protein 25 (SNAP-25) (Synapto
0,115384615	Q92499|DDX1_HUMAN	ATP-dependent RNA helicase DDX1 (EC 3.6.1.-) (DEAD
0,105263158	Q15560|TCEA2_HUMAN	Transcription elongation factor A protein 2 (Transcription
0,017241379	Q9BVJ6|UT14A_HUMAN	U3 small nucleolar RNA-associated protein 14 homolog A
0,053191489	P12270|TPR_HUMAN	Nucleoprotein TPR - Homo sapiens (Human)
0,11627907	Q9P2K5|MYEF2_HUMAN	Myelin expression factor 2 (MyEF-2) (MST156) - Homo sa
0,028571429	O60716|CTND1_HUMAN	Catenin delta-1 (p120 catenin) (p120(ctn)) (Cadherin-ass
0,086956522	O75937|DNJC8_HUMAN	DnaJ homolog subfamily C member 8 (Splicing protein sp
0,051724138	Q9NSE4|SYIM_HUMAN	Isoleucyl-tRNA synthetase, mitochondrial precursor (EC 6
0,060606061	P51532|SMCA4_HUMAN	Probable global transcription activator SNF2L4 (EC 3.6.1
0,136363636	Q12905|ILF2_HUMAN	Interleukin enhancer-binding factor 2 (Nuclear factor of ac
0,105263158	Q9HB71|CYBP_HUMAN	Calcyclin-binding protein (CacyBP) (hCacyBP) (Siah-inter
0,142857143	Q96A72|MGN2_HUMAN; P	Protein mago nashi homolog 2 - Homo sapiens (Human);
0,094017094	Q00610|CLH1_HUMAN	Clathrin heavy chain 1 (CLH-17) - Homo sapiens (Human
0,095238095	O43684|BUB3_HUMAN	Mitotic checkpoint protein BUB3 - Homo sapiens (Human
0,058823529	Q9Y230|RUVB2_HUMAN	RuvB-like 2 (EC 3.6.1.-) (48 kDa TATA box-binding prote
0,073170732	Q02818|NUCB1_HUMAN	Nucleobindin-1 precursor (CALNUC) - Homo sapiens (Hu
0,133333333	P24752|THIL_HUMAN	Acetyl-CoA acetyltransferase, mitochondrial precursor (E
0,08	P40925|MDHC_HUMAN	Malate dehydrogenase, cytoplasmic (EC 1.1.1.37) (Cytos
0,428571429	Q99848|EBP2_HUMAN	Probable rRNA-processing protein EBP2 (EBNA1-binding
0,092307692	P34932|HSP74_HUMAN	Heat shock 70 kDa protein 4 (Heat shock 70-related prote
0,388888889	O00148|DDX39_HUMAN	ATP-dependent RNA helicase DDX39 (EC 3.6.1.-) (DEAD
0,153846154	Q9NPD8|UBE2T_HUMAN	Ubiquitin-conjugating enzyme E2 T (EC 6.3.2.19) (Ubiqui
0,263157895	Q09028|RBBP4_HUMAN	Histone-binding protein RBBP4 (Retinoblastoma-binding
0,006756757	Q96K17|BT3L4_HUMAN	Transcription factor BTF3 homolog 4 (Basic transcription
0,2	P05386|RLA1_HUMAN	60S acidic ribosomal protein P1 - Homo sapiens (Human
0,120481928	P26640|SYV_HUMAN	Valyl-tRNA synthetase (EC 6.1.1.9) (Valine--tRNA ligase)
0,142857143	P55957|BID_HUMAN	BH3-interacting domain death agonist (BID) (p22 BID) [C
0,111111111	Q9P0L0|VAPA_HUMAN	Vesicle-associated membrane protein-associated protein
0,051948052	Q5TF21|CF174_HUMAN	Uncharacterized protein C6orf174 precursor - Homo sapi
0,031746032	P98175|RBM10_HUMAN	RNA-binding protein 10 (RNA-binding motif protein 10) - I
0,148148148	O95433|AHSA1_HUMAN	Activator of 90 kDa heat shock protein ATPase homolog
0,044117647	P12814|ACTN1_HUMAN	Alpha-actinin-1 (Alpha-actinin cytoskeletal isoform) (Non-
0,333333333	Q15185|TEBP_HUMAN	Prostaglandin E synthase 3 (EC 5.3.99.3) (Cytosolic pros
0,095238095	Q8TAA3|PSA7L_HUMAN	Proteasome subunit alpha type 7-like (EC 3.4.25.1) - Hor
0,15	P62993|GRB2_HUMAN	Growth factor receptor-bound protein 2 (Adapter protein G
0,0625	P54577|SYYC_HUMAN	Tyrosyl-tRNA synthetase, cytoplasmic (EC 6.1.1.1) (Tyro
0,046511628	P07197|NFM_HUMAN	Neurofilament triplet M protein (160 kDa neurofilament pr
0,064516129	Q9BUQ8|DDX23_HUMAN	Probable ATP-dependent RNA helicase DDX23 (EC 3.6.1
0,111111111	Q00688|FKBP3_HUMAN	FK506-binding protein 3 (EC 5.2.1.8) (Peptidyl-prolyl cis-t
0,130434783	P52907|CAZA1_HUMAN	F-actin capping protein subunit alpha-1 (CapZ alpha-1) -
0,060606061	P22234|PUR6_HUMAN	Multifunctional protein ADE2 [Includes: Phosphoribosylan
0,166666667	P30084|ECHM_HUMAN	Enoyl-CoA hydratase, mitochondrial precursor (EC 4.2.1.
0,166666667	Q08945|SSRP1_HUMAN	FACT complex subunit SSRP1 (Facilitates chromatin tran
0,076923077	Q15773|MLF2_HUMAN	Myeloid leukemia factor 2 (Myelodysplasia-myeloid leuke
0,051282051	Q8N1G4|LRC47_HUMAN	Leucine-rich repeat-containing protein 47 - Homo sapiens

0,1875	O43809|CPSF5_HUMAN	Cleavage and polyadenylation specificity factor 5 (Cleava
0,045454545	Q9NX63|CHCH3_HUMAN	Coiled-coil-helix-coiled-coil-helix domain-containing prote
0,111111111	P48643|TCPE_HUMAN	T-complex protein 1 subunit epsilon (TCP-1-epsilon) (CC
0,037037037	Q9HDC9|APMAP_HUMAN	Adipocyte plasma membrane-associated protein (BSCv p
0,0625	Q9H0D6|XRN2_HUMAN	5'-3' exoribonuclease 2 (EC 3.1.13.-) (DHM1-like protein)
0,045454545	P63244|GBLP_HUMAN	Guanine nucleotide-binding protein subunit beta 2-like 1 (
0,095238095	Q15293|RCN1_HUMAN	Reticulocalbin-1 precursor - Homo sapiens (Human)
0,153846154	Q9UBE0|SAE1_HUMAN	SUMO-activating enzyme subunit 1 (Ubiquitin-like 1-activ
0,05	O75569|PRKRA_HUMAN	Interferon-inducible double stranded RNA-dependent pro
0,105263158	P62906|RL10A_HUMAN	60S ribosomal protein L10a (CSA-19) - Homo sapiens (H
0,075471698	Q13813|SPTA2_HUMAN	Spectrin alpha chain, brain (Spectrin, non-erythroid alpha
0,042553191	Q9UKV3|ACINU_HUMAN	Apoptotic chromatin condensation inducer in the nucleus
0,016	P49750|YLPM1_HUMAN	YLP motif-containing protein 1 (Nuclear protein ZAP3) (Z
0,09375	P62191|PRS4_HUMAN	26S protease regulatory subunit 4 (P26s4) (Proteasome
0,054545455	O43143|DHX15_HUMAN	Putative pre-mRNA-splicing factor ATP-dependent RNA h
0,05	O96013|PAK4_HUMAN	Serine/threonine-protein kinase PAK 4 (EC 2.7.11.1) (p2
0,136363636	P40429|RL13A_HUMAN	60S ribosomal protein L13a (23 kDa highly basic protein)
0,117647059	O15145|ARPC3_HUMAN	Actin-related protein 2/3 complex subunit 3 (ARP2/3 com
0,125	Q08752|PPID_HUMAN	40 kDa peptidyl-prolyl cis-trans isomerase (EC 5.2.1.8) (F
0,185185185	Q12765|SCRN1_HUMAN	Secernin-1 - Homo sapiens (Human)
0,042105263	Q13428|TCOF_HUMAN	Treacle protein (Treacher Collins syndrome protein) - Hor
0,166666667	O75934|BCAS2_HUMAN	Breast carcinoma amplified sequence 2 (DNA amplified ir
0,043478261	P51114|FXR1_HUMAN	Fragile X mental retardation syndrome-related protein 1 (
0,105263158	Q15691|MARE1_HUMAN	Microtubule-associated protein RP/EB family member 1 (
0,36	P14314|GLU2B_HUMAN	Glucosidase 2 subunit beta precursor (Glucosidase II sub
0,111111111	P36954|RPB9_HUMAN	DNA-directed RNA polymerase II subunit I (EC 2.7.7.6) (I
0,074074074	Q9ULR0|ISY1_HUMAN	Pre-mRNA-splicing factor ISY1 homolog - Homo sapiens
0,181818182	O43852|CALU_HUMAN	Calumenin precursor (Crocalbin) (IEF SSP 9302) - Homo
0,206896552	Q8N8S7|ENAH_HUMAN	Protein enabled homolog - Homo sapiens (Human)
0,133333333	O96008|TOM40_HUMAN	Probable mitochondrial import receptor subunit TOM40 h
0,233333333	Q9UQ80|PA2G4_HUMAN	Proliferation-associated protein 2G4 (Cell cycle protein p
0,108433735	Q9Y5B9|SPT16_HUMAN	FACT complex subunit SPT16 (Facilitates chromatin tran
0,090909091	P61457|PHS_HUMAN	Pterin-4-alpha-carbinolamine dehydratase (EC 4.2.1.96)
0,0625	Q9Y3I0|CV028_HUMAN	UPF0027 protein C22orf28 - Homo sapiens (Human)
0,5	P62979|RS27A_HUMAN	40S ribosomal protein S27a - Homo sapiens (Human)
0,2	P43686|PRS6B_HUMAN	26S protease regulatory subunit 6B (Proteasome 26S sul
0,304347826	Q99733|NP1L4_HUMAN	Nucleosome assembly protein 1-like 4 (Nucleosome asse
0,083333333	P78347|GTF2I_HUMAN	General transcription factor II-I (GTFII-I) (TFII-I) (Bruton t
0,136363636	P29401|TKT_HUMAN	Transketolase (EC 2.2.1.1) (TK) - Homo sapiens (Human
0,037037037	Q969G3|SMCE1_HUMAN	SWI/SNF-related matrix-associated actin-dependent regu
0,071428571	Q9HD42|CHM1A_HUMAN	Charged multivesicular body protein 1a (Chromatin-modi
0,153846154	Q9Y266|NUDC_HUMAN	Nuclear migration protein nudC (Nuclear distribution prote
0,230769231	P30048|PRDX3_HUMAN	Thioredoxin-dependent peroxide reductase, mitochondria
0,214285714	P61163|ACTZ_HUMAN	Alpha-centractin (Centractin) (Centrosome-associated ac
0,117647059	P82930|RT34_HUMAN	Mitochondrial 28S ribosomal protein S34 (S34mt) (MRP-
0,142857143	P50395|GDIB_HUMAN	Rab GDP dissociation inhibitor beta (Rab GDI beta) (Gua
0,055555556	P53041|PPP5_HUMAN	Serine/threonine-protein phosphatase 5 (EC 3.1.3.16) (P
0,076470588	P35579|MYH9_HUMAN	Myosin-9 (Myosin heavy chain, nonmuscle IIa) (Nonmusc
0,137931034	P39748|FEN1_HUMAN	Flap endonuclease 1 (EC 3.1.-.-) (Flap structure-specific
0,115384615	P26368|U2AF2_HUMAN	Splicing factor U2AF 65 kDa subunit (U2 auxiliary factor 6
0,073529412	Q14566|MCM6_HUMAN	DNA replication licensing factor MCM6 (p105MCM) - Hor
0,049382716	Q9C0J8|WDR33_HUMAN	WD repeat protein 33 (WD repeat protein WDC146) - Hor
0,225806452	P55084|ECHB_HUMAN	Trifunctional enzyme subunit beta, mitochondrial precurs
0,071428571	O15173|PGRC2_HUMAN	Membrane-associated progesterone receptor component
0,147058824	Q9UN86|G3BP2_HUMAN	Ras GTPase-activating protein-binding protein 2 (G3BP-2
0,026845638	O43290|SNUT1_HUMAN	U4/U6.U5 tri-snRNP-associated protein 1 (U4/U6.U5 tri-s

0,125	P12956|KU70_HUMAN	ATP-dependent DNA helicase 2 subunit 1 (ATP-depende
0,170731707	P06744|G6PI_HUMAN	Glucose-6-phosphate isomerase (EC 5.3.1.9) (GPI) (Pho
0,117647059	O75351|VPS4B_HUMAN	Vacuolar protein sorting-associating protein 4B (Suppres
0,058823529	O43633|CHM2A_HUMAN	Charged multivesicular body protein 2a (Chromatin-modif
0,034482759	P11310|ACADM_HUMAN	Medium-chain specific acyl-CoA dehydrogenase, mitocho
0,137931034	Q9UJU6|DBNL_HUMAN	Drebrin-like protein (SH3 domain-containing protein 7) (D
0,132352941	P22314|UBE1_HUMAN	Ubiquitin-activating enzyme E1 (A1S9 protein) - Homo sa
0,088235294	P35527|K1C9_HUMAN	Keratin, type I cytoskeletal 9 (Cytokeratin-9) (CK-9) (Kera
0,15	Q9BPU6|DPYL5_HUMAN	Dihydropyrimidinase-related protein 5 (DRP-5) (ULIP6 pr
0,137931034	P31689|DNJA1_HUMAN	DnaJ homolog subfamily A member 1 (Heat shock 40 kD
0,052631579	P49821|NDUV1_HUMAN	NADH dehydrogenase [ubiquinone] flavoprotein 1, mitoch
0,191176471	P46821|MAP1B_HUMAN	Microtubule-associated protein 1B (MAP 1B) [Contains: M
0,076923077	Q16637|SMN_HUMAN	Survival motor neuron protein (Component of gems 1) (G
0,277777778	P55209|NP1L1_HUMAN	Nucleosome assembly protein 1-like 1 (NAP-1-related pro
0,107142857	Q14683|SMC1A_HUMAN	Structural maintenance of chromosomes protein 1A (SMC
0,138888889	P48735|IDHP_HUMAN	Isocitrate dehydrogenase [NADP], mitochondrial precurso
0,166666667	P38919|DDX48_HUMAN	Probable ATP-dependent RNA helicase DDX48 (EC 3.6.
0,162790698	P50990|TCPQ_HUMAN	T-complex protein 1 subunit theta (TCP-1-theta) (CCT-th
0,06557377	Q16891|IMMT_HUMAN	Mitochondrial inner membrane protein (Mitofilin) (p87/89)
0,181818182	O75367|H2AY_HUMAN	Core histone macro-H2A.1 (Histone macroH2A1) (mH2A
0,083333333	Q9Y224|CN166_HUMAN	Protein C14orf166 - Homo sapiens (Human)
0,066666667	P04181|OAT_HUMAN	Ornithine aminotransferase, mitochondrial precursor (EC
0,125	P09012|SNRPA_HUMAN	U1 small nuclear ribonucleoprotein A (U1 snRNP protein
0,181818182	P60520|GBRL2_HUMAN	Gamma-aminobutyric acid receptor-associated protein-lik
0,098039216	Q15459|SF3A1_HUMAN	Splicing factor 3 subunit 1 (Spliceosome-associated prote
0,117647059	P21281|VATB2_HUMAN	Vacuolar ATP synthase subunit B, brain isoform (EC 3.6.
0,192307692	Q96AG4|LRC59_HUMAN	Leucine-rich repeat-containing protein 59 - Homo sapiens
0,142857143	P51665|PSD7_HUMAN	26S proteasome non-ATPase regulatory subunit 7 (26S p
0,096774194	Q9NTK5|GTPB9_HUMAN	Putative GTP-binding protein 9 - Homo sapiens (Human)
0,166666667	P27695|APEX1_HUMAN	DNA-(apurinic or apyrimidinic site) lyase (EC 4.2.99.18) (
0,025	Q05682|CALD1_HUMAN	Caldesmon (CDM) - Homo sapiens (Human)
0,15	Q99426|TBCB_HUMAN	Tubulin-specific chaperone B (Tubulin folding cofactor B)
0,272727273	P63279|UBC9_HUMAN	SUMO-conjugating enzyme UBC9 (EC 6.3.2.-) (SUMO-pr
0,125	P08579|RU2B_HUMAN	U2 small nuclear ribonucleoprotein B'' - Homo sapiens (H
0,277777778	Q96EP5|DAZP1_HUMAN	DAZ-associated protein 1 (Deleted in azoospermia-assoc
0,125	Q16630|CPSF6_HUMAN	Cleavage and polyadenylation specificity factor 6 (Cleava
0,15	O14531|DPYL4_HUMAN	Dihydropyrimidinase-related protein 4 (DRP-4) (Collapsin
0,2	Q7Z7K6|PRR6_HUMAN	Proline-rich protein 6 (Nuclear protein p30) - Homo sapie
0,06779661	Q9UPT8|CS007_HUMAN	Zinc finger CCCH domain-containing protein C19orf7 - H
0,222222222	Q9UFG5|CS025_HUMAN	Uncharacterized protein C19orf25 - Homo sapiens (Huma
0,2	Q9NQ50|RM40_HUMAN	39S ribosomal protein L40, mitochondrial precursor (L40
0,08	Q15019|SEPT2_HUMAN	Septin-2 (Protein NEDD5) - Homo sapiens (Human)
0,095238095	P23396|RS3_HUMAN	40S ribosomal protein S3 - Homo sapiens (Human)
0,212121212	P49411|EFTU_HUMAN	Elongation factor Tu, mitochondrial precursor (EF-Tu) (P
0,230769231	P63208|SKP1_HUMAN	S-phase kinase-associated protein 1A (Cyclin A/CDK2-as
0,133333333	P68402|PA1B2_HUMAN	Platelet-activating factor acetylhydrolase IB subunit beta
0,176470588	Q16181|SEPT7_HUMAN	Septin-7 (CDC10 protein homolog) - Homo sapiens (Hum
0,193548387	Q16543|CDC37_HUMAN	Hsp90 co-chaperone Cdc37 (Hsp90 chaperone protein ki
0,15	P49368|TCPG_HUMAN	T-complex protein 1 subunit gamma (TCP-1-gamma) (CC
0,2	Q9UNZ5|L10K_HUMAN	Leydig cell tumor 10 kDa protein homolog - Homo sapien
0,111111111	Q8IWS0|PHF6_HUMAN	PHD finger protein 6 (PHD-like zinc finger protein) - Hom
0,185185185	P25685|DNJB1_HUMAN	DnaJ homolog subfamily B member 1 (Heat shock 40 kD
0,184210526	Q14194|DPYL1_HUMAN	Dihydropyrimidinase-related protein 1 (DRP-1) (Collapsin
0,178082192	Q7KZF4|SND1_HUMAN	Staphylococcal nuclease domain-containing protein 1 (p1
0,078313253	P35580|MYH10_HUMAN	Myosin-10 (Myosin heavy chain, nonmuscle IIb) (Nonmus
0,061538462	P17480|UBF1_HUMAN	Nucleolar transcription factor 1 (Upstream-binding factor

0,076923077	P35998|PRS7_HUMAN	26S protease regulatory subunit 7 (Proteasome 26S subu
0,2	O75607|NPM3_HUMAN	Nucleoplasmin-3 - Homo sapiens (Human)
0,181818182	P10645|CMGA_HUMAN	Chromogranin A precursor (CgA) (Pituitary secretory prot
0,125	O96000|NDUBA_HUMAN	NADH dehydrogenase [ubiquinone] 1 beta subcomplex s
0,19047619	P25786|PSA1_HUMAN	Proteasome subunit alpha type 1 (EC 3.4.25.1) (Proteaso
0,111111111	P62888|RL30_HUMAN	60S ribosomal protein L30 - Homo sapiens (Human)
0,2	Q9UHV9|PFD2_HUMAN	Prefoldin subunit 2 - Homo sapiens (Human)
0,034482759	Q9NVI7|ATD3A_HUMAN	ATPase family AAA domain-containing protein 3A - Homo
0,111111111	Q9NR30|DDX21_HUMAN	Nucleolar RNA helicase 2 (EC 3.6.1.-) (Nucleolar RNA he
0,109090909	Q14978|NOLC1_HUMAN	Nucleolar phosphoprotein p130 (Nucleolar 130 kDa prote
0,0625	P49756|RBM25_HUMAN	Probable RNA-binding protein 25 (RNA-binding motif pro
0,096774194	O14745|NHERF_HUMAN	Ezrin-radixin-moesin-binding phosphoprotein 50 (EBP50)
0,058823529	P51116|FXR2_HUMAN	Fragile X mental retardation syndrome-related protein 2 -
0,133333333	Q9Y2B0|MSAP_HUMAN	MIR-interacting saposin-like protein precursor (Transmen
0,11627907	O00571|DDX3X_HUMAN	ATP-dependent RNA helicase DDX3X (EC 3.6.1.-) (DEAI
0,173913043	P06493|CDC2_HUMAN	Cell division control protein 2 homolog (EC 2.7.11.22) (E(
0,109090909	Q03252|LMNB2_HUMAN	Lamin-B2 - Homo sapiens (Human)
0,142857143	Q9Y543|HES2_HUMAN	Transcription factor HES-2 (Hairy and enhancer of split 2
0,119047619	Q96I24|FUBP3_HUMAN	Far upstream element-binding protein 3 (FUSE-binding p
0,125	Q9BPW8|NIPS1_HUMAN	Protein NipSnap1 - Homo sapiens (Human)
0,240740741	P49321|NASP_HUMAN	Nuclear autoantigenic sperm protein (NASP) - Homo sap
0,05	Q9BUJ2|HNRL1_HUMAN	Heterogeneous nuclear ribonucleoprotein U-like protein 1
0,222222222	Q8IXM3|RM41_HUMAN	39S ribosomal protein L41, mitochondrial precursor (L41r
0,125	P40227|TCPZ_HUMAN	T-complex protein 1 subunit zeta (TCP-1-zeta) (CCT-zeta
0,086956522	Q9NSV0|PRR8_HUMAN	Proline-rich protein 8 - Homo sapiens (Human)
0,085106383	Q9UHD8|SEPT9_HUMAN	Septin-9 (MLL septin-like fusion protein) (MLL septin-like
0,071428571	P62277|RS13_HUMAN	40S ribosomal protein S13 - Homo sapiens (Human)
0,044444444	P33240|CSTF2_HUMAN	Cleavage stimulation factor 64 kDa subunit (CSTF 64 kD
0,181818182	Q9P0M6|H2AW_HUMAN	Core histone macro-H2A.2 (Histone macroH2A2) (mH2A
0,19047619	Q92841|DDX17_HUMAN	Probable ATP-dependent RNA helicase DDX17 (EC 3.6.
0,333333333	P99999|CYC_HUMAN	Cytochrome c - Homo sapiens (Human)
0,105263158	Q9HAV7|GRPE1_HUMAN	GrpE protein homolog 1, mitochondrial precursor (Mt-Grp
0,153846154	P48047|ATPO_HUMAN	ATP synthase O subunit, mitochondrial precursor (EC 3.6
0,153846154	P36639|8ODP_HUMAN	7,8-dihydro-8-oxoguanine triphosphatase (EC 3.1.6.-) (8-
0,109375	Q14151|SAFB2_HUMAN	Scaffold attachment factor B2 - Homo sapiens (Human)
0,136363636	Q9P015|RM15_HUMAN	39S ribosomal protein L15, mitochondrial precursor (L15r
0,214285714	O43447|PPIH_HUMAN	Peptidyl-prolyl cis-trans isomerase H (EC 5.2.1.8) (PPIas
0,3	P60953|CDC42_HUMAN	Cell division control protein 42 homolog precursor (G25K
0,363636364	P13693|TCTP_HUMAN	Translationally-controlled tumor protein (TCTP) (p23) (His
0,095238095	O76021|RL1D1_HUMAN	Ribosomal L1 domain-containing protein 1 (Cellular sene
0,222222222	O43920|NDUS5_HUMAN	NADH dehydrogenase [ubiquinone] iron-sulfur protein 5 (
0,363636364	O00264|PGRC1_HUMAN	Membrane-associated progesterone receptor component
0,157894737	O43396|TXNL1_HUMAN	Thioredoxin-like protein 1 (32 kDa thioredoxin-related pro
0,142857143	Q14195|DPYL3_HUMAN	Dihydropyrimidinase-related protein 3 (DRP-3) (Unc-33-li
0,142857143	P62241|RS8_HUMAN	40S ribosomal protein S8 - Homo sapiens (Human)
0,193548387	P00505|AATM_HUMAN	Aspartate aminotransferase, mitochondrial precursor (EC
0,189655172	Q14697|GANAB_HUMAN	Neutral alpha-glucosidase AB precursor (EC 3.2.1.84) (G
0,266666667	Q13185|CBX3_HUMAN	Chromobox protein homolog 3 (Heterochromatin protein
0,126984127	P49736|MCM2_HUMAN	DNA replication licensing factor MCM2 (Minichromosome
0,4	P62310|LSM3_HUMAN	U6 snRNA-associated Sm-like protein LSm3 - Homo sapi
0,285714286	P05455|LA_HUMAN	Lupus La protein (Sjoegren syndrome type B antigen) (S
0,222222222	P56211|ARP19_HUMAN	cAMP-regulated phosphoprotein 19 (ARPP-19) - Homo s
0,178571429	Q13283|G3BP1_HUMAN	Ras GTPase-activating protein-binding protein 1 (EC 3.6.
0,083333333	Q13526|PIN1_HUMAN	Peptidyl-prolyl cis-trans isomerase NIMA-interacting 1 (E
0,272727273	Q8WUD4|CCD12_HUMAN	Coiled-coil domain-containing protein 12 - Homo sapiens
0,12195122	P78371|TCPB_HUMAN	T-complex protein 1 subunit beta (TCP-1-beta) (CCT-bet

0,136363636	P05388|RLA0_HUMAN	60S acidic ribosomal protein P0 (L10E) - Homo sapiens (
0,086021505	P55265|DSRAD_HUMAN	Double-stranded RNA-specific adenosine deaminase (EC
0,156626506	Q08211|DHX9_HUMAN	ATP-dependent RNA helicase A (EC 3.6.1.-) (Nuclear DN
0,276595745	Q13263|TIF1B_HUMAN	Transcription intermediary factor 1-beta (TIF1-beta) (Tripa
0,333333333	O15355|PP2CG_HUMAN	Protein phosphatase 2C isoform gamma (EC 3.1.3.16) (P
0,230769231	P60660|MYL6_HUMAN	Myosin light polypeptide 6 (Smooth muscle and nonmusc
0,25	P62304|RUXE_HUMAN	Small nuclear ribonucleoprotein E (snRNP-E) (Sm protein
0,166666667	Q9BXP5|ARS2_HUMAN	Arsenite-resistance protein 2 - Homo sapiens (Human)
0,184210526	Q9P258|RCC2_HUMAN	Protein RCC2 (Telophase disk protein of 60 kDa) (RCC1-
0,083333333	O14737|PDCD5_HUMAN	Programmed cell death protein 5 (Protein TFAR19) (TF-1
0,363636364	P09382|LEG1_HUMAN	Galectin-1 (Lectin galactoside-binding soluble 1) (Beta-ga
0,161764706	Q15424|SAFB1_HUMAN	Scaffold attachment factor B (Scaffold attachment factor I
0,1	Q9Y2W2|WBP11_HUMAN	WW domain-binding protein 11 (WBP-11) (SH3 domain-b
0,275862069	P00338|LDHA_HUMAN	L-lactate dehydrogenase A chain (EC 1.1.1.27) (LDH-A) (
0,096774194	Q9UNF1|MAGD2_HUMAN	Melanoma-associated antigen D2 (MAGE-D2 antigen) (M
0,363636364	P53999|TCP4_HUMAN	Activated RNA polymerase II transcriptional coactivator p
0,105263158	P21796|VDAC1_HUMAN	Voltage-dependent anion-selective channel protein 1 (VD
0,214285714	O00233|PSMD9_HUMAN	26S proteasome non-ATPase regulatory subunit 9 (26S p
0,230769231	Q15637|SF01_HUMAN	Splicing factor 1 (Zinc finger protein 162) (Transcription fa
0,4	P13073|COX41_HUMAN	Cytochrome c oxidase subunit 4 isoform 1, mitochondrial
0,166666667	P31150|GDIA_HUMAN	Rab GDP dissociation inhibitor alpha (Rab GDI alpha) (G
0,105263158	P17677|NEUM_HUMAN	Neuromodulin (Axonal membrane protein GAP-43) (Grow
0,363636364	P26599|PTBP1_HUMAN	Polypyrimidine tract-binding protein 1 (PTB) (Heterogene
0,8	P62841|RS15_HUMAN	40S ribosomal protein S15 (RIG protein) - Homo sapiens
0,15	Q15717|ELAV1_HUMAN	ELAV-like protein 1 (Hu-antigen R) (HuR) - Homo sapiens
0,46875	P27824|CALX_HUMAN	Calnexin precursor (Major histocompatibility complex clas
0,166666667	P05387|RLA2_HUMAN	60S acidic ribosomal protein P2 (Renal carcinoma antige
0,03125	Q9Y6H1|CHCH2_HUMAN	Coiled-coil-helix-coiled-coil-helix domain-containing prote
0,25	Q96PK6|RBM14_HUMAN	RNA-binding protein 14 (RNA-binding motif protein 14) (F
0,196078431	Q13310|PABP4_HUMAN	Polyadenylate-binding protein 4 (Poly(A)-binding protein 4
0,058823529	Q13405|RM49_HUMAN	Mitochondrial 39S ribosomal protein L49 (L49mt) (MRP-L
0,333333333	P39023|RL3_HUMAN	60S ribosomal protein L3 (HIV-1 TAR RNA-binding protei
0,25	P45973|CBX5_HUMAN	Chromobox protein homolog 5 (Heterochromatin protein 1
0,166666667	Q00325|MPCP_HUMAN	Phosphate carrier protein, mitochondrial precursor (PTP)
0,304347826	P29692|EF1D_HUMAN	Elongation factor 1-delta (EF-1-delta) (Antigen NY-CO-4)
0,274509804	P13667|PDIA4_HUMAN	Protein disulfide-isomerase A4 precursor (EC 5.3.4.1) (Pr
0,054054054	Q86U42|PABP2_HUMAN	Polyadenylate-binding protein 2 (Poly(A)-binding protein 2
0,214285714	P61586|RHOA_HUMAN	Transforming protein RhoA precursor (H12) - Homo sapie
0,2	Q99406|TYBN_HUMAN	NB thymosin beta (Thymosin-like protein 8) - Homo sapie
0,095238095	Q15287|RNPS1_HUMAN	RNA-binding protein with serine-rich domain 1 (SR-relate
0,133333333	P25787|PSA2_HUMAN	Proteasome subunit alpha type 2 (EC 3.4.25.1) (Proteaso
0,102941176	P11387|TOP1_HUMAN	DNA topoisomerase 1 (EC 5.99.1.2) (DNA topoisomerase
0,5	P63173|RL38_HUMAN	60S ribosomal protein L38 - Homo sapiens (Human)
0,25	P14927|UCR6_HUMAN	Ubiquinol-cytochrome c reductase complex 14 kDa protei
0,222222222	Q9Y5S9|RBM8A_HUMAN	RNA-binding protein 8A (RNA-binding motif protein 8A) (I
0,176470588	Q96NC0|ZMAT2_HUMAN	Zinc finger matrin-type protein 2 - Homo sapiens (Human
0,5	P25398|RS12_HUMAN	40S ribosomal protein S12 - Homo sapiens (Human)
0,128205128	Q5BKZ1|ZN326_HUMAN	Zinc finger protein 326 - Homo sapiens (Human)
0,328125	P13639|EF2_HUMAN	Elongation factor 2 (EF-2) - Homo sapiens (Human)
0,117647059	P52298|NCBP2_HUMAN	Nuclear cap-binding protein subunit 2 (20 kDa nuclear ca
0,126984127	P33992|MCM5_HUMAN	DNA replication licensing factor MCM5 (CDC46 homolog)
0,5	Q15651|HMGN3_HUMAN	High mobility group nucleosome-binding domain-containi
0,15625	P35269|T2FA_HUMAN	Transcription initiation factor IIF subunit alpha (EC 2.7.11
0,666666667	P14406|CX7A2_HUMAN	Cytochrome c oxidase polypeptide VIIa-liver/heart, mitoch
0,642857143	Q01105|SET_HUMAN	Protein SET (Phosphatase 2A inhibitor I2PP2A) (I-2PP2A
0,714285714	P62263|RS14_HUMAN	40S ribosomal protein S14 - Homo sapiens (Human)

0,181818182	Q96CT7|CC124_HUMAN	Coiled-coil domain-containing protein 124 - Homo sapien
0,166666667	P60842|IF4A1_HUMAN	Eukaryotic initiation factor 4A-I (EC 3.6.1.-) (ATP-depend
0,4	P42677|RS27_HUMAN	40S ribosomal protein S27 (Metallopan-stimulin 1) (MPS-
0,090909091	P62753|RS6_HUMAN	40S ribosomal protein S6 (Phosphoprotein NP33) - Homo
0,333333333	O14813|PHX2A_HUMAN	Paired mesoderm homeobox protein 2A (Paired-like hom
0,123287671	Q9NYF8|BCLF1_HUMAN	Bcl-2-associated transcription factor 1 (Btf) - Homo sapie
0,166666667	P62244|RS15A_HUMAN	40S ribosomal protein S15a - Homo sapiens (Human)
0,391304348	P07195|LDHB_HUMAN	L-lactate dehydrogenase B chain (EC 1.1.1.27) (LDH-B) (
0,125	Q08170|SFRS4_HUMAN	Splicing factor, arginine/serine-rich 4 (Pre-mRNA-splicing
0,5	P10599|THIO_HUMAN	Thioredoxin (Trx) (ATL-derived factor) (ADF) (Surface-as
0,222222222	Q01844|EWS_HUMAN	RNA-binding protein EWS (EWS oncogene) (Ewing sarco
1,75	P20962|PTMS_HUMAN	Parathymosin - Homo sapiens (Human)
0,444444444	P62851|RS25_HUMAN	40S ribosomal protein S25 - Homo sapiens (Human)
0,161290323	P35659|DEK_HUMAN	Protein DEK - Homo sapiens (Human)
0,368421053	P54727|RD23B_HUMAN	UV excision repair protein RAD23 homolog B (hHR23B) (
0,052173913	O75525|KHDR3_HUMAN	KH domain-containing, RNA-binding, signal transduction-
0,3	Q9Y2V2|CHSP1_HUMAN	Calcium-regulated heat stable protein 1 (Calcium-regulat
0,181818182	O60828|PQBP1_HUMAN	Polyglutamine-binding protein 1 (Polyglutamine tract-bind
0,34	Q12906|ILF3_HUMAN	Interleukin enhancer-binding factor 3 (Nuclear factor of ac
0,25	P55072|TERA_HUMAN	Transitional endoplasmic reticulum ATPase (TER ATPas
0,4	Q92688|AN32B_HUMAN	Acidic leucine-rich nuclear phosphoprotein 32 family mem
0,25	P30050|RL12_HUMAN	60S ribosomal protein L12 - Homo sapiens (Human)
0,052238806	Q14152|IF3A_HUMAN	Eukaryotic translation initiation factor 3 subunit 10 (eIF-3
0,173913043	P26378|ELAV4_HUMAN	ELAV-like protein 4 (Paraneoplastic encephalomyelitis ar
0,291666667	P52597|HNRPF_HUMAN	Heterogeneous nuclear ribonucleoprotein F (hnRNP F) (N
0,125	P50402|EMD_HUMAN	Emerin - Homo sapiens (Human)
0,230769231	P24666|PPAC_HUMAN	Low molecular weight phosphotyrosine protein phosphata
0,571428571	P29966|MARCS_HUMAN	Myristoylated alanine-rich C-kinase substrate (MARCKS)
0,228070175	P02545|LMNA_HUMAN	Lamin-A/C (70 kDa lamin) (Renal carcinoma antigen NY-
0,347826087	Q15365|PCBP1_HUMAN	Poly(rC)-binding protein 1 (Alpha-CP1) (hnRNP-E1) (Nuc
0,086956522	Q14980|NUMA1_HUMAN	Nuclear mitotic apparatus protein 1 (NuMA protein) (SP-H
0,181818182	P52815|RM12_HUMAN	39S ribosomal protein L12, mitochondrial precursor (L12r
0,9	P09496|CLCA_HUMAN	Clathrin light chain A (Lca) - Homo sapiens (Human)
0,130434783	Q13595|TRA2A_HUMAN	Transformer-2 protein homolog (TRA-2 alpha) - Homo sa
0,375	P35244|RFA3_HUMAN	Replication protein A 14 kDa subunit (RP-A) (RF-A) (Rep
0,181818182	Q9P0M9|RM27_HUMAN	Mitochondrial 39S ribosomal protein L27 (L27mt) (MRP-L
0,295454545	O43390|HNRPR_HUMAN	Heterogeneous nuclear ribonucleoprotein R (hnRNP R) -
0,222222222	P63165|SUMO1_HUMAN	Small ubiquitin-related modifier 1 precursor (SUMO-1) (S
0,272727273	P55769|NH2L1_HUMAN	NHP2-like protein 1 (High mobility group-like nuclear prot
0,25	Q99714|HCD2_HUMAN	3-hydroxyacyl-CoA dehydrogenase type-2 (EC 1.1.1.35)
0,413793103	P26641|EF1G_HUMAN	Elongation factor 1-gamma (EF-1-gamma) (eEF-1B gamr
0,166666667	Q02543|RL18A_HUMAN	60S ribosomal protein L18a - Homo sapiens (Human)
0,285714286	P18859|ATP5J_HUMAN	ATP synthase coupling factor 6, mitochondrial precursor (
0,407407407	P55795|HNRH2_HUMAN	Heterogeneous nuclear ribonucleoprotein H' (hnRNP H')
0,153846154	P29762|RABP1_HUMAN	Cellular retinoic acid-binding protein 1 (Cellular retinoic ac
0,322580645	Q15084|PDIA6_HUMAN	Protein disulfide-isomerase A6 precursor (EC 5.3.4.1) (Pr
0,274509804	P15311|EZRI_HUMAN	Ezrin (p81) (Cytovillin) (Villin-2) - Homo sapiens (Human)
0,291139241	P09874|PARP1_HUMAN	Poly [ADP-ribose] polymerase 1 (EC 2.4.2.30) (PARP-1)
0,192307692	Q9NQ29|LUC7L_HUMAN	Putative RNA-binding protein Luc7-like 1 (SR+89) (Putati
0,240740741	P23588|IF4B_HUMAN	Eukaryotic translation initiation factor 4B (eIF-4B) - Homo
0,666666667	P01303|NPY_HUMAN	Neuropeptide Y precursor [Contains: Neuropeptide Y (Ne
0,466666667	P14866|HNRPL_HUMAN	Heterogeneous nuclear ribonucleoprotein L (hnRNP L) - I
0,275862069	Q13435|SF3B2_HUMAN	Splicing factor 3B subunit 2 (Spliceosome-associated pro
0,5	P24534|EF1B_HUMAN	Elongation factor 1-beta (EF-1-beta) - Homo sapiens (Hu
0,45	P43243|MATR3_HUMAN	Matrin-3 - Homo sapiens (Human)
0,133333333	O60493|SNX3_HUMAN	Sorting nexin-3 (Protein SDP3) - Homo sapiens (Human)

0,162790698	Q16352|AINX_HUMAN	Alpha-internexin (Alpha-Inx) (66 kDa neurofilament protei
0,714285714	Q71UI9|H2AV_HUMAN	Histone H2AV (H2A.F/Z) - Homo sapiens (Human)
0,285714286	Q9BWJ5|SF3B5_HUMAN	Splicing factor 3B subunit 5 (SF3b5) (Pre-mRNA-splicing
0,083333333	O43678|NDUA2_HUMAN	NADH dehydrogenase [ubiquinone] 1 alpha subcomplex
0,55	O75475|PSIP1_HUMAN	PC4 and SFRS1-interacting protein (Lens epithelium-deri
0,307692308	P32969|RL9_HUMAN	60S ribosomal protein L9 - Homo sapiens (Human)
0,5	O60812|HNRCL_HUMAN	Heterogeneous nuclear ribonucleoprotein C-like 1 (hnRN
0,173913043	Q00059|TFAM_HUMAN	Transcription factor A, mitochondrial precursor (mtTFA) (I
0,333333333	O60506|HNRPQ_HUMAN	Heterogeneous nuclear ribonucleoprotein Q (hnRNP Q) (
0,235294118	P84098|RL19_HUMAN	60S ribosomal protein L19 - Homo sapiens (Human)
0,153846154	P18615|NELFE_HUMAN	Negative elongation factor E (NELF-E) (RD protein) - Hor
0,533333333	P39687|AN32A_HUMAN	Acidic leucine-rich nuclear phosphoprotein 32 family men
0,178571429	Q9Y383|LC7L2_HUMAN	Putative RNA-binding protein Luc7-like 2 - Homo sapiens
0,15	Q9UI15|TAGL3_HUMAN	Transgelin-3 (Neuronal protein NP25) (Neuronal protein 2
0,333333333	P52943|CRIP2_HUMAN	Cysteine-rich protein 2 (CRP2) (Protein ESP1) - Homo sa
0,142857143	P20290|BTF3_HUMAN	Transcription factor BTF3 (RNA polymerase B transcriptic
0,5	P20674|COX5A_HUMAN	Cytochrome c oxidase subunit 5A, mitochondrial precurso
0,428571429	P05114|HMGN1_HUMAN	Nonhistone chromosomal protein HMG-14 (High-mobility
0,333333333	Q16658|FSCN1_HUMAN	Fascin (Singed-like protein) (55 kDa actin-bundling protei
0,5	P30044|PRDX5_HUMAN	Peroxiredoxin-5, mitochondrial precursor (EC 1.11.1.15) (
0,176470588	P07305|H10_HUMAN	Histone H1.0 (Histone H1(0)) (Histone H1') - Homo sapie
0,285714286	P11940|PABP1_HUMAN	Polyadenylate-binding protein 1 (Poly(A)-binding protein 1
0,142857143	O15212|PFD6_HUMAN	Prefoldin subunit 6 (Protein Ke2) - Homo sapiens (Humar
0,457627119	P14625|ENPL_HUMAN	Endoplasmin precursor (Heat shock protein 90 kDa beta
0,25	Q96FJ2|DYL2_HUMAN	Dynein light chain 2, cytoplasmic (Dynein light chain LC8-
0,393939394	P07951|TPM2_HUMAN	Tropomyosin beta chain (Tropomyosin 2) (Beta-tropomyo
0,325	Q16555|DPYL2_HUMAN	Dihydropyrimidinase-related protein 2 (DRP-2) (Collapsin
0,333333333	P62913|RL11_HUMAN	60S ribosomal protein L11 (CLL-associated antigen KW-1
0,333333333	P62913|RL11_HUMAN	60S ribosomal protein L11 (CLL-associated antigen KW-1
0,166666667	Q12904|MCA1_HUMAN	Multisynthetase complex auxiliary component p43 [Conta
0,285714286	P83916|CBX1_HUMAN	Chromobox protein homolog 1 (Heterochromatin protein 1
0,419354839	P06576|ATPB_HUMAN	ATP synthase subunit beta, mitochondrial precursor (EC
0,75	P25705|ATPA_HUMAN	ATP synthase subunit alpha, mitochondrial precursor (EC
0,4	P08107|HSP71_HUMAN	Heat shock 70 kDa protein 1 (HSP70.1) (HSP70-1/HSP7
0,75	P49006|MRP_HUMAN	MARCKS-related protein (MARCKS-like protein 1) (Macr
0,411764706	Q16643|DREB_HUMAN	Drebrin (Developmentally-regulated brain protein) - Homo
0,333333333	P30049|ATPD_HUMAN	ATP synthase delta chain, mitochondrial precursor (EC 3
0,266666667	O60869|EDF1_HUMAN	Endothelial differentiation-related factor 1 (EDF-1) (Multip
0,444444444	P35268|RL22_HUMAN	60S ribosomal protein L22 (Epstein-Barr virus small RNA
0,105263158	P54819|KAD2_HUMAN	Adenylate kinase isoenzyme 2, mitochondrial (EC 2.7.4.3
0,421052632	P08865|RSSA_HUMAN	40S ribosomal protein SA (p40) (34/67 kDa laminin recep
0,368421053	P51858|HDGF_HUMAN	Hepatoma-derived growth factor (HDGF) (High-mobility g
0,192307692	P12236|ADT3_HUMAN	ADP/ATP translocase 3 (Adenine nucleotide translocator
0,259259259	Q13247|SFRS6_HUMAN	Splicing factor, arginine/serine-rich 6 (Pre-mRNA-splicing
0,466666667	Q99497|PARK7_HUMAN	Protein DJ-1 (Oncogene DJ1) - Homo sapiens (Human)
0,428571429	P36578|RL4_HUMAN	60S ribosomal protein L4 (L1) - Homo sapiens (Human)
0,384615385	P00441|SODC_HUMAN	Superoxide dismutase [Cu-Zn] (EC 1.15.1.1) - Homo sap
0,294117647	Q15102|PA1B3_HUMAN	Platelet-activating factor acetylhydrolase IB subunit gamr
0,333333333	P31948|STIP1_HUMAN	Stress-induced-phosphoprotein 1 (STI1) (Hsc70/Hsp90-o
0,466666667	P46782|RS5_HUMAN	40S ribosomal protein S5 - Homo sapiens (Human)
0,375	P62258|1433E_HUMAN	14-3-3 protein epsilon (14-3-3E) - Homo sapiens (Human
0,25	O75348|VATG1_HUMAN	Vacuolar ATP synthase subunit G 1 (EC 3.6.3.14) (V-ATI
0,315789474	P30041|PRDX6_HUMAN	Peroxiredoxin-6 (EC 1.11.1.15) (Antioxidant protein 2) (1-
0,194805195	Q9Y2W1|TR150_HUMAN	Thyroid hormone receptor-associated protein 3 (Thyroid I
0,533333333	P62826|RAN_HUMAN	GTP-binding nuclear protein Ran (GTPase Ran) (Ras-like
0,238095238	P18124|RL7_HUMAN	60S ribosomal protein L7 - Homo sapiens (Human)

0,12244898	Q13573|SNW1_HUMAN	SNW domain-containing protein 1 (Nuclear protein SkiP)
0,340909091	P07237|PDIA1_HUMAN	Protein disulfide-isomerase precursor (EC 5.3.4.1) (PDI)
0,419354839	P09104|ENOG_HUMAN	Gamma-enolase (EC 4.2.1.11) (2-phospho-D-glycerate h
0,666666667	P31946|1433B_HUMAN	14-3-3 protein beta/alpha (Protein kinase C inhibitor prote
0,45	P62917|RL8_HUMAN	60S ribosomal protein L8 - Homo sapiens (Human)
0,375	P09669|COX6C_HUMAN	Cytochrome c oxidase polypeptide VIc precursor (EC 1.9
0,636363636	P27348|1433T_HUMAN	14-3-3 protein theta (14-3-3 protein tau) (14-3-3 protein T
0,666666667	P27797|CALR_HUMAN	Calreticulin precursor (CRP55) (Calregulin) (HACBP) (EF
0,069364162	Q9UQ35|SRRM2_HUMAN	Serine/arginine repetitive matrix protein 2 (Serine/arginine
0,909090909	P62266|RS23_HUMAN	40S ribosomal protein S23 - Homo sapiens (Human)
0,416666667	O00193|SMAP_HUMAN	Small acidic protein - Homo sapiens (Human)
0,142857143	P46781|RS9_HUMAN	40S ribosomal protein S9 - Homo sapiens (Human)
0,363636364	P62701|RS4X_HUMAN	40S ribosomal protein S4, X isoform (Single copy abunda
0,428571429	P62312|LSM6_HUMAN	U6 snRNA-associated Sm-like protein LSm6 (Sm protein
0,142857143	P46776|RL27A_HUMAN	60S ribosomal protein L27a - Homo sapiens (Human)
0,518518519	P46777|RL5_HUMAN	60S ribosomal protein L5 - Homo sapiens (Human)
0,454545455	P37108|SRP14_HUMAN	Signal recognition particle 14 kDa protein (SRP14) (18 kD
0,285714286	Q9H910|HN1L_HUMAN	Hematological and neurological expressed 1-like protein
0,24	P15880|RS2_HUMAN	40S ribosomal protein S2 (S4) (LLRep3 protein) - Homo s
0,32	P61247|RS3A_HUMAN	40S ribosomal protein S3a - Homo sapiens (Human)
0,333333333	P46778|RL21_HUMAN	60S ribosomal protein L21 - Homo sapiens (Human)
0,571428571	P43487|RANG_HUMAN	Ran-specific GTPase-activating protein (Ran-binding prot
0,3	Q9UI30|TR112_HUMAN	TRM112-like protein - Homo sapiens (Human)
0,466666667	Q5H9L2|TCAL5_HUMAN	Transcription elongation factor A protein-like 5 (TCEA-like
0,759259259	P07900|HS90A_HUMAN	Heat shock protein HSP 90-alpha (HSP 86) (Renal carcir
0,346153846	Q02878|RL6_HUMAN	60S ribosomal protein L6 (TAX-responsive enhancer eler
0,333333333	P62633|CNBP_HUMAN	Cellular nucleic acid-binding protein (CNBP) (Zinc finger
0,333333333	P17844|DDX5_HUMAN	Probable ATP-dependent RNA helicase DDX5 (EC 3.6.1.
0,285714286	Q8WW12|PCNP_HUMAN	PEST proteolytic signal-containing nuclear protein (PEST
0,588235294	Q9H1E3|NUCKS_HUMAN	Nuclear ubiquitous casein and cyclin-dependent kinases
0,363636364	P62847|RS24_HUMAN	40S ribosomal protein S24 - Homo sapiens (Human)
0,375	P38646|GRP75_HUMAN	Stress-70 protein, mitochondrial precursor (75 kDa glucos
0,25	Q9Y5J6|TIM9B_HUMAN	Mitochondrial import inner membrane translocase subuni
0,166666667	O95182|NDUA7_HUMAN	NADH dehydrogenase [ubiquinone] 1 alpha subcomplex
0,16	P22087|FBRL_HUMAN	rRNA 2'-O-methyltransferase fibrillarin (EC 2.1.1.-) (34 kD
0,291666667	P62995|TRA2B_HUMAN	Arginine/serine-rich-splicing factor 10 (Transformer-2-beta
0,3125	P04792|HSPB1_HUMAN	Heat-shock protein beta-1 (HspB1) (Heat shock 27 kDa p
0,535714286	Q05639|EF1A2_HUMAN	Elongation factor 1-alpha 2 (EF-1-alpha-2) (Elongation fa
0,217391304	Q15181|IPYR_HUMAN	Inorganic pyrophosphatase (EC 3.6.1.1) (Pyrophosphate
0,5	P23284|PPIB_HUMAN	Peptidyl-prolyl cis-trans isomerase B precursor (EC 5.2.1
0,625	P60866|RS20_HUMAN	40S ribosomal protein S20 - Homo sapiens (Human)
0,45	P52272|HNRPM_HUMAN	Heterogeneous nuclear ribonucleoprotein M (hnRNP M) -
0,111111111	Q9Y237|PIN4_HUMAN	Peptidyl-prolyl cis-trans isomerase NIMA-interacting 4 (E
0,375	P04080|CYTB_HUMAN	Cystatin-B (Stefin-B) (Liver thiol proteinase inhibitor) (CPI
0,166666667	Q13243|SFRS5_HUMAN	Splicing factor, arginine/serine-rich 5 (Pre-mRNA-splicing
0,25	Q9Y3B4|PM14_HUMAN	Pre-mRNA branch site protein p14 (SF3B 14 kDa subunit
0,52	P12277|KCRB_HUMAN	Creatine kinase B-type (EC 2.7.3.2) (Creatine kinase B c
0,1875	P46779|RL28_HUMAN	60S ribosomal protein L28 - Homo sapiens (Human)
0,320754717	P20700|LMNB1_HUMAN	Lamin-B1 - Homo sapiens (Human)
0,25	P14854|CX6B1_HUMAN	Cytochrome c oxidase subunit VIb isoform 1 (EC 1.9.3.1)
0,785714286	P68104|EF1A1_HUMAN	Elongation factor 1-alpha 1 (EF-1-alpha-1) (Elongation fa
0,333333333	P62910|RL32_HUMAN	60S ribosomal protein L32 - Homo sapiens (Human)
0,6	P52565|GDIR_HUMAN	Rho GDP-dissociation inhibitor 1 (Rho GDI 1) (Rho-GDI a
0,16	P51991|ROA3_HUMAN	Heterogeneous nuclear ribonucleoprotein A3 (hnRNP A3
0,608695652	P63104|1433Z_HUMAN	14-3-3 protein zeta/delta (Protein kinase C inhibitor protei
0,6	P62829|RL23_HUMAN	60S ribosomal protein L23 (Ribosomal protein L17) - Hon

0,45	P62424|RL7A_HUMAN	60S ribosomal protein L7a (Surfeit locus protein 3) (PLA-:
1	Q9UII2|ATIF1_HUMAN	ATPase inhibitor, mitochondrial precursor - Homo sapiens
0,470588235	P06753|TPM3_HUMAN	Tropomyosin alpha-3 chain (Tropomyosin-3) (Tropomyos
0,761904762	Q99729|ROAA_HUMAN	Heterogeneous nuclear ribonucleoprotein A/B (hnRNP A/
0,592592593	P31943|HNRH1_HUMAN	Heterogeneous nuclear ribonucleoprotein H (hnRNP H) -
0,451612903	Q07666|SAM68_HUMAN	KH domain-containing, RNA-binding, signal transduction-
0,266666667	Q15819|UB2V2_HUMAN	Ubiquitin-conjugating enzyme E2 variant 2 (MMS2) (Ente
0,769230769	P68366|TBA1_HUMAN	Tubulin alpha-1 chain (Alpha-tubulin 1) (Testis-specific al
0,794871795	P10809|CH60_HUMAN	60 kDa heat shock protein, mitochondrial precursor (Hsp6
0,583333333	P61353|RL27_HUMAN	60S ribosomal protein L27 - Homo sapiens (Human)
0,803921569	P08238|HS90B_HUMAN	Heat shock protein HSP 90-beta (HSP 84) (HSP 90) - Ho
0,540540541	P00558|PGK1_HUMAN	Phosphoglycerate kinase 1 (EC 2.7.2.3) (Primer recogniti
0,5	P62316|SMD2_HUMAN	Small nuclear ribonucleoprotein Sm D2 (snRNP core prot
0,571428571	P62269|RS18_HUMAN	40S ribosomal protein S18 (Ke-3) (Ke3) - Homo sapiens
0,416666667	P18077|RL35A_HUMAN	60S ribosomal protein L35a - Homo sapiens (Human)
0,636363636	P61981|1433G_HUMAN	14-3-3 protein gamma (Protein kinase C inhibitor protein
0,222222222	P62854|RS26_HUMAN	40S ribosomal protein S26 - Homo sapiens (Human)
1	Q9Y2S6|CCD72_HUMAN	Coiled-coil domain-containing protein 72 - Homo sapiens
0,384615385	P08621|RU17_HUMAN	U1 small nuclear ribonucleoprotein 70 kDa (U1 snRNP 70
0,333333333	Q01081|U2AF1_HUMAN	Splicing factor U2AF 35 kDa subunit (U2 auxiliary factor 3
0,2	Q99623|PHB2_HUMAN	Prohibitin-2 (B-cell receptor-associated protein BAP37) (F
0,909090909	P62158|CALM_HUMAN	Calmodulin (CaM) - Homo sapiens (Human)
0,3125	P80723|BASP_HUMAN	Brain acid soluble protein 1 (BASP1 protein) (Neuronal a:
0,477272727	P26038|MOES_HUMAN	Moesin (Membrane-organizing extension spike protein) -
0,428571429	P61088|UBE2N_HUMAN	Ubiquitin-conjugating enzyme E2 N (EC 6.3.2.19) (Ubiqui
0,393939394	P42167|LAP2B_HUMAN	Lamina-associated polypeptide 2, isoforms beta/gamma (
0,461538462	P09211|GSTP1_HUMAN	Glutathione S-transferase P (EC 2.5.1.18) (GST class-pi)
0,709677419	P61978|HNRPK_HUMAN	Heterogeneous nuclear ribonucleoprotein K (hnRNP K) (T
0,575	P30101|PDIA3_HUMAN	Protein disulfide-isomerase A3 precursor (EC 5.3.4.1) (Di
1	P83881|RL36A_HUMAN	60S ribosomal protein L36a (60S ribosomal protein L44)
0,5	P08708|RS17_HUMAN	40S ribosomal protein S17 - Homo sapiens (Human)
0,466666667	P18621|RL17_HUMAN	60S ribosomal protein L17 (L23) - Homo sapiens (Humar
0,363636364	P31942|HNRH3_HUMAN	Heterogeneous nuclear ribonucleoprotein H3 (hnRNP H3
0,235294118	P14678|RSMB_HUMAN	Small nuclear ribonucleoprotein-associated proteins B an
0,333333333	O75531|BAF_HUMAN	Barrier-to-autointegration factor (Breakpoint cluster regior
1	P62308|RUXG_HUMAN	Small nuclear ribonucleoprotein G (snRNP-G) (Sm protei
0,421052632	P50914|RL14_HUMAN	60S ribosomal protein L14 (CAG-ISL 7) - Homo sapiens (
0,666666667	P83731|RL24_HUMAN	60S ribosomal protein L24 (Ribosomal protein L30) - Hon
0,6	P61927|RL37_HUMAN	60S ribosomal protein L37 (G1.16) - Homo sapiens (Hum
7	P06454|PTMA_HUMAN	Prothymosin alpha [Contains: Thymosin alpha-1] - Homo
0,363636364	Q9GZT3|SLIRP_HUMAN	SRA stem-loop-interacting RNA-binding protein, mitochor
0,772727273	P40926|MDHM_HUMAN	Malate dehydrogenase, mitochondrial precursor (EC 1.1.
1,19047619	P06748|NPM_HUMAN	Nucleophosmin (NPM) (Nucleolar phosphoprotein B23) (I
1,4	P14174|MIF_HUMAN	Macrophage migration inhibitory factor (MIF) (Phenylpyru
0,564102564	O15240|VGF_HUMAN	Neurosecretory protein VGF precursor - Homo sapiens (H
0,423076923	P35637|FUS_HUMAN	RNA-binding protein FUS (Oncogene FUS) (Oncogene T
0,78	Q00839|HNRPU_HUMAN	Heterogeneous nuclear ribonucleoprotein U (hnRNP U) (:
0,583333333	P47914|RL29_HUMAN	60S ribosomal protein L29 (Cell surface heparin-binding p
0,7	P27635|RL10_HUMAN	60S ribosomal protein L10 (QM protein) (Tumor suppress
0,307692308	P09455|RET1_HUMAN	Retinol-binding protein I, cellular (Cellular retinol-binding p
0,333333333	Q15843|NEDD8_HUMAN	NEDD8 precursor (Ubiquitin-like protein Nedd8) (Neddylin
0,285714286	P62249|RS16_HUMAN	40S ribosomal protein S16 - Homo sapiens (Human)
0,88	P62736|ACTA_HUMAN	Actin, aortic smooth muscle (Alpha-actin-2) - Homo sapie
0,611111111	P18669|PGAM1_HUMAN	Phosphoglycerate mutase 1 (EC 5.4.2.1) (EC 5.4.2.4) (E(
0,642857143	P62280|RS11_HUMAN	40S ribosomal protein S11 - Homo sapiens (Human)
0,689655172	P67936|TPM4_HUMAN	Tropomyosin alpha-4 chain (Tropomyosin-4) (TM30p1) -

0,347826087	Q92945|FUBP2_HUMAN	Far upstream element-binding protein 2 (FUSE-binding p
1,090909091	Q14103|HNRPD_HUMAN	Heterogeneous nuclear ribonucleoprotein D0 (hnRNP D0
0,571428571	P22392|NDKB_HUMAN	Nucleoside diphosphate kinase B (EC 2.7.4.6) (NDK B) (I
0,75	P62942|FKB1A_HUMAN	FK506-binding protein 1A (EC 5.2.1.8) (Peptidyl-prolyl cis
0,375	P62081|RS7_HUMAN	40S ribosomal protein S7 - Homo sapiens (Human)
0,155555556	Q92804|RBP56_HUMAN	TATA-binding protein-associated factor 2N (RNA-binding
0,75	P49458|SRP09_HUMAN	Signal recognition particle 9 kDa protein (SRP9) - Homo s
0,642857143	P61254|RL26_HUMAN	60S ribosomal protein L26 - Homo sapiens (Human)
0,424242424	Q8NC51|PAIRB_HUMAN	Plasminogen activator inhibitor 1 RNA-binding protein (PA
0,2	Q9NPE3|NOLA3_HUMAN	H/ACA ribonucleoprotein complex subunit 3 (Nucleolar pr
0,571428571	P56385|ATP5I_HUMAN	ATP synthase e chain, mitochondrial (EC 3.6.3.14) - Hom
0,76744186	P11021|GRP78_HUMAN	78 kDa glucose-regulated protein precursor (GRP 78) (H
0,5	Q86V81|THOC4_HUMAN	THO complex subunit 4 (Tho4) (Ally of AML-1 and LEF-1
0,3	O75494|FUSIP_HUMAN	FUS-interacting serine-arginine-rich protein 1 (TLS-assoc
0,473684211	P26373|RL13_HUMAN	60S ribosomal protein L13 (Breast basic conserved prote
1,266666667	Q13151|ROA0_HUMAN	Heterogeneous nuclear ribonucleoprotein A0 (hnRNP A0
0,884615385	P04075|ALDOA_HUMAN	Fructose-bisphosphate aldolase A (EC 4.1.2.13) (Muscle-
0,666666667	P32119|PRDX2_HUMAN	Peroxiredoxin-2 (EC 1.11.1.15) (Thioredoxin peroxidase 1
0,333333333	P61313|RL15_HUMAN	60S ribosomal protein L15 - Homo sapiens (Human)
0,347826087	P35232|PHB_HUMAN	Prohibitin - Homo sapiens (Human)
0,642857143	P33316|DUT_HUMAN	Deoxyuridine 5'-triphosphate nucleotidohydrolase, mitoch
0,384615385	Q07020|RL18_HUMAN	60S ribosomal protein L18 - Homo sapiens (Human)
0,470588235	Q92522|H1X_HUMAN	Histone H1x - Homo sapiens (Human)
0,571428571	Q9H3K6|BOLA2_HUMAN	BolA-like protein 2 - Homo sapiens (Human)
1,181818182	P04350|TBB4_HUMAN	Tubulin beta-4 chain (Tubulin 5 beta) - Homo sapiens (Hu
1	P84090|ERH_HUMAN	Enhancer of rudimentary homolog - Homo sapiens (Huma
0,391304348	Q15056|IF4H_HUMAN	Eukaryotic translation initiation factor 4H (eIF-4H) (Williar
0,666666667	P10606|COX5B_HUMAN	Cytochrome c oxidase subunit 5B, mitochondrial precurso
0,25	Q9Y3Y2|CA077_HUMAN	Uncharacterized protein C1orf77 - Homo sapiens (Humar
1	Q71U36|TBA3_HUMAN	Tubulin alpha-3 chain (Alpha-tubulin 3) (Tubulin B-alpha-
1	P11142|HSP7C_HUMAN	Heat shock cognate 71 kDa protein (Heat shock 70 kDa p
0,4	P62318|SMD3_HUMAN	Small nuclear ribonucleoprotein Sm D3 (snRNP core prot
0,5	Q96C90|PP14B_HUMAN	Protein phosphatase 1 regulatory subunit 14B (Phospholi
1,5	O15347|HMGB3_HUMAN	High mobility group protein B3 (High mobility group protei
1,714285714	P0C0S5|H2AZ_HUMAN	Histone H2A.Z (H2A/z) - Homo sapiens (Human)
1,347826087	P68371|TBB2C_HUMAN	Tubulin beta-2C chain (Tubulin beta-2 chain) - Homo sap
1,173913043	Q13509|TBB3_HUMAN	Tubulin beta-3 chain (Tubulin beta-III) (Tubulin beta-4) - H
0,75	P37802|TAGL2_HUMAN	Transgelin-2 (SM22-alpha homolog) - Homo sapiens (Hu
0,533333333	P30086|PEBP1_HUMAN	Phosphatidylethanolamine-binding protein 1 (PEBP-1) (P
0,428571429	P61024|CKS1_HUMAN	Cyclin-dependent kinases regulatory subunit 1 (CKS-1) -
0,555555556	P49773|HINT1_HUMAN	Histidine triad nucleotide-binding protein 1 (Adenosine 5'-
0,666666667	Q15233|NONO_HUMAN	Non-POU domain-containing octamer-binding protein (No
0,923076923	P19338|NUCL_HUMAN	Nucleolin (Protein C23) - Homo sapiens (Human)
0,75	Q06830|PRDX1_HUMAN	Peroxiredoxin-1 (EC 1.11.1.15) (Thioredoxin peroxidase 2
0,568181818	P14618|KPYM_HUMAN	Pyruvate kinase isozymes M1/M2 (EC 2.7.1.40) (Pyruvat
0,558139535	Q96AE4|FUBP1_HUMAN	Far upstream element-binding protein 1 (FUSE-binding p
0,785714286	P98179|RBM3_HUMAN	Putative RNA-binding protein 3 (RNA-binding motif protei
1,909090909	Q16695|H31T_HUMAN	Histone H3.1t (H3t) (H3/t) (H3/g) - Homo sapiens (Humar
0,25	P62273|RS29_HUMAN	40S ribosomal protein S29 - Homo sapiens (Human)
0,76744186	P23246|SFPQ_HUMAN	Splicing factor, proline- and glutamine-rich (Polypyrimidin
0,647058824	Q13442|HAP28_HUMAN	28 kDa heat- and acid-stable phosphoprotein (PDGF-ass
0,444444444	Q14011|CIRBP_HUMAN	Cold-inducible RNA-binding protein (Glycine-rich RNA-bir
0,538461538	Q04837|SSB_HUMAN	Single-stranded DNA-binding protein, mitochondrial precu
1,045454545	P04406|G3P_HUMAN	Glyceraldehyde-3-phosphate dehydrogenase (EC 1.2.1.1
1,347826087	Q9BVA1|TBB2B_HUMAN	Tubulin beta-2B chain - Homo sapiens (Human)
1,304347826	Q13885|TBB2A_HUMAN	Tubulin beta-2A chain - Homo sapiens (Human)

0,782608696	P67809|YBOX1_HUMAN	Nuclease sensitive element-binding protein 1 (Y-box-bind
0,625	P08670|VIME_HUMAN	Vimentin - Homo sapiens (Human)
0,5	P07108|ACBP_HUMAN	Acyl-CoA-binding protein (ACBP) (Diazepam-binding inhi
1,545454545	P07437|TBB5_HUMAN	Tubulin beta chain (Tubulin beta-5 chain) - Homo sapiens
0,6	P84103|SFRS3_HUMAN	Splicing factor, arginine/serine-rich 3 (Pre-mRNA-splicing
0,714285714	Q9Y3U8|RL36_HUMAN	60S ribosomal protein L36 - Homo sapiens (Human)
0,32	Q13242|SFRS9_HUMAN	Splicing factor, arginine/serine-rich 9 (Pre-mRNA-splicing
0,923076923	P07910|HNRPC_HUMAN	Heterogeneous nuclear ribonucleoproteins C1/C2 (hnRN
0,6	Q01130|SFRS2_HUMAN	Splicing factor, arginine/serine-rich 2 (Splicing factor SC3
1,4	P60709|ACTB_HUMAN	Actin, cytoplasmic 1 (Beta-actin) - Homo sapiens (Human
1	P62861|RS30_HUMAN	40S ribosomal protein S30 - Homo sapiens (Human)
0,384615385	P15531|NDKA_HUMAN	Nucleoside diphosphate kinase A (EC 2.7.4.6) (NDK A) (
0,941176471	P09936|UCHL1_HUMAN	Ubiquitin carboxyl-terminal hydrolase isozyme L1 (EC 3.4
0,666666667	P63241|IF5A1_HUMAN	Eukaryotic translation initiation factor 5A-1 (eIF-5A-1) (eIF
3,222222222	P42766|RL35_HUMAN	60S ribosomal protein L35 - Homo sapiens (Human)
0,666666667	P42766|RL35_HUMAN	60S ribosomal protein L35 - Homo sapiens (Human)
0,636363636	P62750|RL23A_HUMAN	60S ribosomal protein L23a - Homo sapiens (Human)
1,290322581	P06733|ENOA_HUMAN	Alpha-enolase (EC 4.2.1.11) (2-phospho-D-glycerate hyd
2,555555556	P16104|H2AX_HUMAN	Histone H2A.x (H2a/x) - Homo sapiens (Human)
1	P07737|PROF1_HUMAN	Profilin-1 (Profilin I) - Homo sapiens (Human)
1,423076923	P09651|ROA1_HUMAN	Heterogeneous nuclear ribonucleoprotein A1 (Helix-desta
2,933333333	P09429|HMGB1_HUMAN	High mobility group protein B1 (High mobility group protei
0,333333333	Q16629|SFRS7_HUMAN	Splicing factor, arginine/serine-rich 7 (Splicing factor 9G8
0,5	Q07955|SFRS1_HUMAN	Splicing factor, arginine/serine-rich 1 (pre-mRNA-splicing
4,571428571	P0C0S8|H2A1_HUMAN	Histone H2A type 1 (H2A.1) - Homo sapiens (Human)
3,857142857	Q8IUE6|H2A2B_HUMAN	Histone H2A type 2-B - Homo sapiens (Human)
1,052631579	P60174|TPIS_HUMAN	Triosephosphate isomerase (EC 5.3.1.1) (TIM) (Triose-ph
0,764705882	P46783|RS10_HUMAN	40S ribosomal protein S10 - Homo sapiens (Human)
1,777777778	P16402|H13_HUMAN	Histone H1.3 (Histone H1c) - Homo sapiens (Human)
0,857142857	P17096|HMGA1_HUMAN	High mobility group protein HMG-I/HMG-Y (HMG-I(Y)) (H
1,1	O75347|TBCA_HUMAN	Tubulin-specific chaperone A (Tubulin-folding cofactor A)
1,428571429	P61956|SUMO2_HUMAN	Small ubiquitin-related modifier 2 precursor (SUMO-2) (U
2	P22626|ROA2_HUMAN	Heterogeneous nuclear ribonucleoproteins A2/B1 (hnRNI
1,090909091	P62899|RL31_HUMAN	60S ribosomal protein L31 - Homo sapiens (Human)
0,818181818	P61604|CH10_HUMAN	10 kDa heat shock protein, mitochondrial (Hsp10) (10 kD
0,682926829	P38159|HNRPG_HUMAN	Heterogeneous nuclear ribonucleoprotein G (hnRNP G) (
1,125	P63220|RS21_HUMAN	40S ribosomal protein S21 - Homo sapiens (Human)
1,5	P62328|TYB4_HUMAN	Thymosin beta-4 (T beta 4) (Fx) [Contains: Hematopoieti
2,733333333	P26583|HMGB2_HUMAN	High mobility group protein B2 (High mobility group protei
1	P62857|RS28_HUMAN	40S ribosomal protein S28 - Homo sapiens (Human)
2,222222222	P16403|H12_HUMAN	Histone H1.2 (Histone H1d) - Homo sapiens (Human)
1,111111111	P62988|UBIQ_HUMAN	Ubiquitin - Homo sapiens (Human)
0,875	P39019|RS19_HUMAN	40S ribosomal protein S19 - Homo sapiens (Human)
1,642857143	P23528|COF1_HUMAN	Cofilin-1 (Cofilin, non-muscle isoform) (18 kDa phosphop
3,416666667	P06899|H2B1J_HUMAN	Histone H2B type 1-J (H2B.r) (H2B/r) (H2B.1) - Homo sa
2,916666667	P33778|H2B1B_HUMAN	Histone H2B type 1-B (H2B.f) (H2B/f) (H2B.1) - Homo sa
3,583333333	Q93079|H2B1H_HUMAN	Histone H2B type 1-H (H2B.j) (H2B/j) - Homo sapiens (H
3,166666667	Q5QNW6|H2B2F_HUMAN	Histone H2B type 2-F - Homo sapiens (Human)
2,833333333	Q99879|H2B1M_HUMAN	Histone H2B type 1-M (H2B.e) (H2B/e) - Homo sapiens (
4	Q16777|H2A2C_HUMAN	Histone H2A type 2-C (H2A-GL101) (H2A/r) - Homo sapi
0,357142857	P63313|TYB10_HUMAN	Thymosin beta-10 - Homo sapiens (Human)
4,333333333	P05204|HMGN2_HUMAN	Nonhistone chromosomal protein HMG-17 (High-mobility
2	P16949|STMN1_HUMAN	Stathmin (Phosphoprotein p19) (pp19) (Oncoprotein 18) (
7	P62805|H4_HUMAN	Histone H4 - Homo sapiens (Human)
3,222222222	P16401|H15_HUMAN	Histone H1.5 (Histone H1a) - Homo sapiens (Human)
2,5	P10412|H14_HUMAN	Histone H1.4 (Histone H1b) - Homo sapiens (Human)

1,933333333	P62937|PPIA_HUMAN	Peptidyl-prolyl cis-trans isomerase A (EC 5.2.1.8) (PPIas[illegible]

ıpiens (Human)
mplex 1 beta-1 subunit) (Beta-adaptin 1) (Adaptor protein complex AP-1 beta-1 subunit) (Golgi adapt

phorase-1) (Cytochrome b5 reductase 3) [Contains: NADH-cytochrome b5 reductase membrane-bou
/l-prolyl cis-trans isomerase) (PPIase) (Rotamase) (65 kDa FK506-binding protein) (FKBP65) (Immur
3.14) - Homo sapiens (Human)
n)
ı 15) (Zinc finger protein RoRet) - Homo sapiens (Human)
D106 antigen) (INCAM-100) - Homo sapiens (Human)
sion molecule) (ALCAM) - Homo sapiens (Human)

153) (153 kDa nucleoporin) - Homo sapiens (Human)
-globulin) (Ba-alpha-2-glycoprotein) [Contains: Alpha-2-HS-glycoprotein chain A; Alpha-2-HS-glycopr
otein) (Retinitis pigmentosa 1 protein) - Homo sapiens (Human)
21) (Nuclear matrix protein 1) (NXP-1) (SCC1 homolog) - Homo sapiens (Human)
ıutase 1) (PGM 1) - Homo sapiens (Human)
1) - Homo sapiens (Human)
scriptional activation subunit 2) (Transcriptional coactivator CRSP150) (Vitamin D3 receptor-interactir
alpha holoenzyme-associated protein P1) (RLF subunit beta) (P102 protein) (P1-MCM3) - Homo sa|
CTP synthetase 1) - Homo sapiens (Human)
sapiens (Human)
SorCS) - Homo sapiens (Human)

22) (PCTAIRE-motif protein kinase 2) - Homo sapiens (Human)
protein 4) (Cell cycle progression restoration gene 3 protein) (Dnj3) (Renal carcinoma antigen NY-RE
GATA-binding factor 3) - Homo sapiens (Human)
Da subunit) (RF-C 37 kDa subunit) (RFC37) (Activator 1 37 kDa subunit) (A1 37 kDa subunit) - Homo
ns (Human)
osin) (AH antigen) - Homo sapiens (Human)
RNA-regulating factor) (BLOCK24 protein) - Homo sapiens (Human)

2.1.1.43) (ASH1-like protein) (Absent small and homeotic disks protein 1 homolog) (huASH1) - Hom
-containing amine oxidase domain-containing protein 2) (BRAF35-HDAC complex protein BHC110) -
sapiens (Human)
3) (93 kDa nucleoporin) - Homo sapiens (Human)
nteracting protein 4) (JIP-4) (JNK-associated leucine-zipper protein) (JLP) (Sperm-associated antigen
lcohol dehydrogenase class III chi chain) (S-(hydroxymethyl)glutathione dehydrogenase) (EC 1.1.1.2
50-6B/29C) - Homo sapiens (Human)
P-binding cassette sub-family C member 3) (Multidrug resistance-associated protein 3) (Multi-specific
eta) (eIF3 p66) (eIF3d) - Homo sapiens (Human)
-protein] S-acetyltransferase (EC 2.3.1.38); [Acyl-carrier-protein] S-malonyltransferase (EC 2.3.1.39);
/ase) (Citrate cleavage enzyme) - Homo sapiens (Human)
omain-containing protein 1A) (SWI/SNF-related, matrix-associated, actin-dependent regulator of chrc
/lulose reductase) (Kidney dicarbonyl reductase) (kiDCR) (Carbonyl reductase II) (Sperm surface prot
6 (P243) - Homo sapiens (Human)
otein 9) (PRKA9) (A-kinase anchor protein 450 kDa) (AKAP 450) (A-kinase anchor protein 350 kDa)
philin-A2) (SH3 domain protein 2B) (Extra eleven-nineteen leukemia fusion gene) (EEN) (EEN fusior
) (V75) [Contains: Vitronectin V65 subunit; Vitronectin V10 subunit; Somatomedin B] - Homo sapiens
um/glucocorticoid-regulated kinase 3) (Serum/glucocorticoid-regulated kinase-like) - Homo sapiens (H
ns (Human)
bosomal RNA-processing protein 44) (DIS3 protein homolog) - Homo sapiens (Human)
ase II) (TPP-II) (Tripeptidyl aminopeptidase) - Homo sapiens (Human)

s: Enoyl-CoA hydratase (EC 4.2.1.17); 3,2-trans-enoyl-CoA isomerase (EC 5.3.3.8); 3-hydroxyacyl-C
ronal protein 1) (Nervous system-specific RNA-binding protein Hel-N1) - Homo sapiens (Human)
WD repeat protein 50) - Homo sapiens (Human)
in-beta) (Bullous pemphigoid antigen) (BPA) (Hemidesmosomal plaque protein) (Dystonia musculoru
sor (Epidermal growth factor-like 2) (Multiple epidermal growth factor-like domains 3) (Flamingo 1) - I
1) - Homo sapiens (Human)
n)
Syne-2) (Synaptic nuclear envelope protein 2) (Nucleus and actin connecting element protein) (Protei
ludes: Phosphoribosylamine--glycine ligase (EC 6.3.4.13) (GARS) (Glycinamide ribonucleotide synth
o sapiens (Human)
no sapiens (Human)
(p102) - Homo sapiens (Human)
proteasome regulatory subunit rpn11) (26S proteasome-associated PAD1 homolog 1) - Homo sapie
Homo sapiens (Human)
no sapiens (Human)
rotein 2) (Centrosomal Nek2-associated protein 1) (C-Nap1) - Homo sapiens (Human)
2, mitochondrial precursor (EC 1.10.2.2) (Core protein II) (Complex III subunit II) - Homo sapiens (Hu
ian)
je molecule derived from yolk sac) - Homo sapiens (Human)
3 (EC 1.14.11.-) (Jumonji domain-containing protein 1B) (Nuclear protein 5qNCA) - Homo sapiens (H

kinase) - Homo sapiens (Human)
KHC) - Homo sapiens (Human)
rial precursor (EC 6.4.1.4) (3-Methylcrotonyl-CoA carboxylase 2) (MCCase subunit beta) (3-methylcro
C-alpha) - Homo sapiens (Human)
uman)
ns (Human)
(Actin cross-linking family protein 7) (Macrophin-1) (Trabeculin-alpha) (620 kDa actin-binding protein)
uman)
phosphate synthase (EC 6.3.5.5); Aspartate carbamoyltransferase (EC 2.1.3.2); Dihydroorotase (EC
a kinase 4) (CCK-4) - Homo sapiens (Human)
P-family protein member 1) (Protein WAVE-1) (Verprolin homology domain-containing protein 1) - Ho
de A] - Homo sapiens (Human)
Renal carcinoma antigen NY-REN-62) - Homo sapiens (Human)
Foocen) (Neuroendocrine-specific protein) (NSP) (Neuroendocrine-specific protein C homolog) (RTN
membrane glycoprotein) (Gp110) - Homo sapiens (Human)
eceptor-interacting protein) (Immunophilin homolog ARA9) (HBV-X-associated protein 2) - Homo sap
crotubule-associated protein) (CTCL tumor antigen se20-10) - Homo sapiens (Human)
in-1) (OTU domain-containing ubiquitin aldehyde-binding protein 1) (Ubiquitin-specific-processing pro
(PKC-A) - Homo sapiens (Human)
nan)
cting protein 3) (M-RIP) (RIP3) (p116Rip) - Homo sapiens (Human)
nber E (LANP-like protein) (LANP-L) - Homo sapiens (Human)
ation initiation factor IF-2) - Homo sapiens (Human)
it beta 2 (Transducin beta chain 2) (G protein beta 2 subunit) - Homo sapiens (Human)
sapiens (Human)
ng enzyme) (PE) - Homo sapiens (Human)
ontaining protein 1) - Homo sapiens (Human)
idative stress-responsive 1 protein) - Homo sapiens (Human)
n)
g) (Autosomal dominant polycystic kidney disease type II protein) (Polycystwin) (R48321) - Homo sap
ciated polypeptide, 30 kDa) (Sin3 corepressor complex subunit SAP30) - Homo sapiens (Human)
(Nucleolar protein interacting with the FHA domain of pKI-67) (hNIFK) (Nucleolar phosphoprotein Nc
na-like PDZ and LIM domains protein) - Homo sapiens (Human)
E-1) - Homo sapiens (Human)
in) (Thyroid autoantigen) (Truncated actin-binding protein) (Truncated ABP) (ABP-280 homolog) (ABI

n)
.) - Homo sapiens (Human)
SH3/WW domain anchor protein in the nucleus) (SWAN) - Homo sapiens (Human)
no sapiens (Human)
ctivator 2) (Cyclin-dependent kinase 5 regulatory subunit 2) (P39) (P39I) - Homo sapiens (Human)
1.-) (ATP-dependent helicase CHD5) (CHD-5) - Homo sapiens (Human)
olog 4) (Polycomb 2 homolog) (Pc2) (hPc2) - Homo sapiens (Human)
it 10 (Phosphatase 1 nuclear targeting subunit) (MHC class I region proline-rich protein CAT53) (FB1
selection protein TIP47) (47 kDa mannose 6-phosphate receptor-binding protein) (47 kDa MPR-bind
ne--tRNA ligase) (AlaRS) (Renal carcinoma antigen NY-REN-42) - Homo sapiens (Human)
ing protein 3) (Formin-binding protein 11) (Huntingtin-interacting protein HYPA) (Huntingtin yeast par
subunit) (RF-C 140 kDa subunit) (Activator 1 140 kDa subunit) (Activator 1 large subunit) (A1 140 kD
n)
GTPase-activating protein 130 kDa subunit) (Rab3-GAP p130) (Rab3-GAP) - Homo sapiens (Human
C 1.8.1.4) (Dihydrolipoamide dehydrogenase) (Glycine cleavage system L protein) - Homo sapiens (
- Homo sapiens (Human)
proteasome regulatory subunit S5A) (Rpn10) (Multiubiquitin chain-binding protein) (Antisecretory fact
Homo sapiens (Human)
, mitochondrial precursor (EC 3.4.21.92) (Endopeptidase Clp) - Homo sapiens (Human)
2.7.10.1) (Tyrosine-protein kinase RSE) (Tyrosine-protein kinase SKY) (Tyrosine-protein kinase DTK)
Da subunit) (RFC38) (Activator 1 38 kDa subunit) (A1 38 kDa subunit) (RF-C 38 kDa subunit) - Homo
1-activated kinase 2) (PAK-2) (PAK65) (Gamma-PAK) (S6/H4 kinase) - Homo sapiens (Human)
a subunit) (G/T mismatch-binding protein) (GTBP) (GTMBP) (p160) - Homo sapiens (Human)

3.5.3.18) (Dimethylargininase-1) (Dimethylarginine dimethylaminohydrolase 1) (DDAHI) (DDAH-1) - H
ain) (Gamma-2-globin) (Hemoglobin gamma-G chain) (Hb F Ggamma) - Homo sapiens (Human); He
bunit ATPase 3) (Tat-binding protein 1) (TBP-1) (Proteasome subunit P50) - Homo sapiens (Human)
.7.6) (RPB1) - Homo sapiens (Human)
inine--tRNA ligase) (ArgRS) - Homo sapiens (Human)
Phosphoribosylaminoimidazolecarboxamide formyltransferase (EC 2.1.2.3) (5-aminoimidazole-4-car
(Aldoketomutase) (Glyoxalase I) (Glx I) (Ketone-aldehyde mutase) (S-D-lactoylglutathione methylgly
mosome-associated polypeptide C) (hCAP-C) (XCAP-C homolog) - Homo sapiens (Human)
-150) (DAP-150) (p150-glued) (p135) - Homo sapiens (Human)
nediate chain 2, cytosolic) (DH IC-2) (Cytoplasmic dynein intermediate chain 2) - Homo sapiens (Hun
(Archain) - Homo sapiens (Human)
thase) [Includes: Methylenetetrahydrofolate dehydrogenase (EC 1.5.1.5); Methenyltetrahydrofolate cy
nsferase) (Tubedown-1 protein) (Tbdn100) (Gastric cancer antigen Ga19) - Homo sapiens (Human)
no sapiens (Human)
pecific gene A14 protein) - Homo sapiens (Human)
eta) (eIF3 p36) (eIF3i) (TGF-beta receptor-interacting protein 1) (TRIP-1) - Homo sapiens (Human)
mplex subunit H) (Barren homolog protein 1) (Chromosome-associated protein H) (hCAP-H) (XCAP-H
proteasome regulatory subunit RPN2) (26S proteasome regulatory subunit S1) (26S proteasome sub
2) (59 kDa serine/threonine-protein kinase) (p59ILK) - Homo sapiens (Human)
oid) - Homo sapiens (Human)
.2.1.3) (ALDH class 2) (ALDHI) (ALDH-E2) - Homo sapiens (Human)
ase 1 regulatory subunit 9B) - Homo sapiens (Human)
PGDH) - Homo sapiens (Human)

subunit 7 (EC 1.6.5.3) (EC 1.6.99.3) (NADH-ubiquinone oxidoreductase B18 subunit) (Complex I-B18)
it beta isoform (EC 3.1.3.16) (PP2A-beta) - Homo sapiens (Human)
Homo sapiens (Human)
B) - Homo sapiens (Human)
sapiens (Human)
athione S-transferase zeta 1) (EC 2.5.1.18) (GSTZ1-1) - Homo sapiens (Human)
ein 2 (Grb10-interacting GYF protein 2) (Trinucleotide repeat-containing protein 15) - Homo sapiens (H
oforms XLas (Adenylate cyclase-stimulating G alpha protein) (Extra large alphas protein) (XLalphas)

ecursor (EC 3.6.3.9) (Sodium pump 1) (Na(+)/K(+) ATPase 1) - Homo sapiens (Human)
lphobindin I) (CBP-I) (Placental anticoagulant protein I) (PAP-I) (PP4) (Thromboplastin inhibitor) (Vas
C 3.6.3.9) (Sodium pump 3) (Na(+)/K(+) ATPase 3) (Alpha(III)) - Homo sapiens (Human)
Translocase of outer membrane 22 kDa subunit homolog) (hTom22) (1C9-2) - Homo sapiens (Human
drial precursor (EC 1.2.4.2) (Alpha-ketoglutarate dehydrogenase) - Homo sapiens (Human)
nit, mitochondrial precursor (EC 1.3.5.1) (Fp) (Flavoprotein subunit of complex II) - Homo sapiens (H
otein) (p63) - Homo sapiens (Human)
.1) (Tousled-like kinase 1) (PKU-beta) - Homo sapiens (Human)
clear factor P97) (Importin 90) - Homo sapiens (Human)

igen MU-RMS-40.14) - Homo sapiens (Human)

man)
11.1) (DNA-PK catalytic subunit) (DNA-PKcs) (DNPK1) (p460) - Homo sapiens (Human)
ein 1 (Zinc finger protein 36) (Zinc finger protein KOX18) - Homo sapiens (Human)
be cdc5-related protein) - Homo sapiens (Human)
(Acyl-peptide hydrolase) (APH) (Acylaminoacyl-peptidase) (Oxidized protein hydrolase) (OPH) (DNF
.6.3.14) - Homo sapiens (Human)
a) - Homo sapiens (Human)
ylbilane synthase) (HMBS) (Pre-uroporphyrinogen synthase) (PBG-D) - Homo sapiens (Human)
age and polyadenylation specificity factor 59 kDa subunit) (CPSF 59 kDa subunit) (Pre-mRNA cleava
R-interacting protein) (WD-40 repeat protein PT-WD) (MAP activator with WD repeats) - Homo sapie
ase) (GlyRS) - Homo sapiens (Human)
proteasome regulatory subunit RPN1) (26S proteasome regulatory subunit S2) (26S proteasome sub
phofructokinase 1) (Phosphohexokinase) (Phosphofructo-1-kinase isozyme A) (PFK-A) (Phosphofruc
tein 62) (SAP 62) (SF3a66) - Homo sapiens (Human)
(ERp28) - Homo sapiens (Human)
inducible protein CIP29) (Cytokine-induced protein of 29 kDa) - Homo sapiens (Human)
-B) (NF-I/B) (CCAAT-box-binding transcription factor) (CTF) (TGGCA-binding protein) - Homo sapien
sapiens (Human)
t Tim8 B (Deafness dystonia protein 2) (DDP-like protein) - Homo sapiens (Human)
no sapiens (Human)
TP-dependent DNA helicase II 80 kDa subunit) (Lupus Ku autoantigen protein p86) (Ku86) (Ku80) (8
ning protein) (TA-WDRP) - Homo sapiens (Human)
160 kDa) (IBP160) - Homo sapiens (Human)
jus antigen) - Homo sapiens (Human)
in) (DBC.1) (DBC-1) (p30 DBC) - Homo sapiens (Human)
5 (Phosphoinositol 3-phosphate-binding protein 2) (PEPP-2) - Homo sapiens (Human)
dent helicase ATRX) (X-linked helicase II) (X-linked nuclear protein) (XNP) (Znf-HX) - Homo sapiens (

nine oxidase type B) (MAO-B) - Homo sapiens (Human)
ta) (eIF3 p116) (eIF3 p110) (eIF3b) (Prt1 homolog) (hPrt1) - Homo sapiens (Human)
(U5 snRNP-specific protein, 116 kDa) (U5-116 kDa) (Elongation factor Tu GTP-binding domain protei
fructokinase 1) (Phosphohexokinase) (Phosphofructo-1-kinase isozyme B) (PFK-B) - Homo sapiens
otic translation initiation factor 2 subunit beta) (eIF-2-beta) - Homo sapiens (Human)
sor (N-CAM 140) (NCAM-140) (CD56 antigen) - Homo sapiens (Human)
complex 1 sigma-1A subunit) (Sigma-adaptin 1A) (Adaptor protein complex AP-1 sigma-1A subunit)
ferase 67 kDa subunit precursor (EC 2.4.1.119) (Ribophorin I) (RPN-I) - Homo sapiens (Human)
ctokinase 1) (Phosphohexokinase) (Phosphofructo-1-kinase isozyme C) (PFK-C) (6-phosphofructoki
rp8) (PRP8 homolog) (220 kDa U5 snRNP-specific protein) (p220) - Homo sapiens (Human)
mogranin C) [Contains: Secretoneurin (SN)] - Homo sapiens (Human)
) (Antigen NY-CO-25) - Homo sapiens (Human)
- Homo sapiens (Human)
edoxin-like protein p46) (Endoplasmic reticulum protein ERp46) - Homo sapiens (Human)
nding protein) (Actin-binding protein 280) (ABP-280) (Nonmuscle filamin) - Homo sapiens (Human)
Ubiquitin-protein ligase) (Ubiquitin carrier protein) (E2(25K)) (Huntingtin-interacting protein 2) (HIP-2)

) (General transcription factor IIE subunit 2) - Homo sapiens (Human)

:NP-associated 102 kDa protein) (U5-102 kDa protein) - Homo sapiens (Human)
pressed 1-like protein) - Homo sapiens (Human)
related protein) - Homo sapiens (Human)
uman)
nt) (MRP-L38) - Homo sapiens (Human)
(U5 snRNP-specific 40 kDa protein) (38 kDa-splicing factor) - Homo sapiens (Human)
D1) - Homo sapiens (Human)
X) (Lis-X) (Doublin) - Homo sapiens (Human)
P secretase) (APPS) [Contains: Cathepsin B light chain; Cathepsin B heavy chain] - Homo sapiens (I
) sapiens (Human)
: 3.6.1.-) (U5 snRNP-specific 200 kDa protein) (U5-200KD) (Activating signal cointegrator 1 complex
nerase II, beta isozyme) - Homo sapiens (Human)
ight intermediate chain 2, cytosolic) (LIC53/55) (LIC-2) - Homo sapiens (Human)
ns (Human)
ding protein) - Homo sapiens (Human)
chain 1) (Beta-II spectrin) (Fodrin beta chain) - Homo sapiens (Human)
lenylosuccinate synthetase, acidic isozyme) (IMP--aspartate ligase 2) (AdSS 2) (AMPSase 2) - Homo
s (Human)
Methionine--tRNA ligase) (MetRS) - Homo sapiens (Human)
e Sm-like protein Sm-x5) (Small nuclear ribonuclear protein D homolog) (Protein G7b) - Homo sapier
n)

ursor (EC 1.18.1.2) (Adrenodoxin reductase) (AR) (Ferredoxin reductase) (Ferredoxin--NADP(+) redu
r protein KOX17) (Retinoic acid suppression protein A) (RSG-A) (Zinc finger and SCAN domain-cont
piens (Human)
orepressor Sin3a) (Histone deacetylase complex subunit Sin3a) - Homo sapiens (Human)
(Phenylalanine--tRNA ligase alpha chain) (PheRS) (CML33) - Homo sapiens (Human)
e--tRNA ligase) (SerRS) - Homo sapiens (Human)
dyl-prolyl isomerase G) (PPIase G) (Rotamase G) (Cyclophilin G) (Clk-associating RS-cyclophilin) (C
P 75) (Tumor necrosis factor type 1 receptor-associated protein) (TRAP-1) (TNFR-associated protein
) (Chromatin assembly factor I p150 subunit) (CAF-I 150 kDa subunit) (CAF-Ip150) - Homo sapiens (
C 1.4.1.3) (GDH) - Homo sapiens (Human)
protein 1) (MALS-1) (Vertebrate lin-7 homolog 1) (Veli-1 protein) (Tax interaction protein 33) (TIP-33)
nt1) (DNA methyltransferase HsaI) (DNA MTase HsaI) (MCMT) (M.HsaI) - Homo sapiens (Human)

a chain (EC 2.7.11.17) (CaM-kinase II delta chain) (CaM kinase II subunit delta) (CaMK-II subunit de
(N-ras upstream gene protein) - Homo sapiens (Human)
lta) (Stimulator of TAR RNA-binding) - Homo sapiens (Human)
tein 23) (RNA-binding region-containing protein 4) (Splicing factor SF2) - Homo sapiens (Human)
HIV-1 Nef-interacting protein) - Homo sapiens (Human)
Ubiquitin thioesterase 14) (Ubiquitin-specific-processing protease 14) (Deubiquitinating enzyme 14) - I
member 1) - Homo sapiens (Human)
sor (TP-alpha) (78 kDa gastrin-binding protein) [Includes: Long-chain enoyl-CoA hydratase (EC 4.2.1
apiens (Human)
tein rhoOGAP) (Rho-related small GTPase protein activator) (CDC42 GTPase-activating protein) (p5
1-Rac1) (Ras-like protein TC25) - Homo sapiens (Human)
) (Dyskerin) (Nucleolar protein family A member 4) (snoRNP protein DKC1) (Nopp140-associated pro
trans isomerase) (PPIase) (Rotamase) (p59 protein) (HSP-binding immunophilin) (HBI) (FKBP52 pro
(Asparagine--tRNA ligase) (AsnRS) - Homo sapiens (Human)

protein) (Paired helical filament-tau) (PHF-tau) - Homo sapiens (Human)
2) (Fumarase) - Homo sapiens (Human)
in kinase) - Homo sapiens (Human)
-prolyl cis-trans isomerase) (PPIase) (Rotamase) (13 kDa FKBP) (FKBP-13) - Homo sapiens (Humar

ein heavy chain 1) (DHC1) (Dynein heavy chain 1, cytoplasmic 1) - Homo sapiens (Human)
nt) (MRP-L17) (LYST-interacting protein 2) - Homo sapiens (Human)
tein III) (Chromobindin-20) (67 kDa calelectrin) (Calphobindin-II) (CPB-II) - Homo sapiens (Human)
translocation-associated gene protein) - Homo sapiens (Human)
biquitin thioesterase 5) (Ubiquitin-specific-processing protease 5) (Deubiquitinating enzyme 5) (Isopep
6PD) - Homo sapiens (Human)
-containing protein 3) (Golgi phosphoprotein 1) (GOLPH1) (Golgi complex-associated protein 1) (GO
(Williams-Beuren syndrome chromosome region 9 protein) (Williams syndrome transcription factor) (h
Human)
S25) - Homo sapiens (Human)
MRP) - Homo sapiens (Human)
Human)
eracting protein) (ALG-2-interacting protein 1) (Hp95) - Homo sapiens (Human)
uman)
ene protein) (GTP-binding protein NGB) - Homo sapiens (Human)
) (P1.1-MCM3) - Homo sapiens (Human)
) (mRNA cap-binding protein) (eIF-4F 25 kDa subunit) - Homo sapiens (Human)
myl-tRNA synthetase (EC 6.1.1.17) (Glutamate--tRNA ligase); Prolyl-tRNA synthetase (EC 6.1.1.15)
er 1 (Voltage-gated potassium channel subunit Kv7.1) (IKs producing slow voltage-gated potassium c
1 (4E-BP1) (eIF4E-binding protein 1) (Phosphorylated heat- and acid-stable protein regulated by insu
otein 155) (SAP 155) (SF3b155) (Pre-mRNA-splicing factor SF3b 155 kDa subunit) - Homo sapiens (H
tivator 28-alpha subunit) (PA28alpha) (PA28a) (Activator of multicatalytic protease subunit 1) (11S re
(TFIIF-beta) (ATP-dependent helicase GTF2F2) (General transcription factor IIF subunit 2) (Transcri
droitin sulfate proteoglycan 6) (Chromosome-associated polypeptide) (hCAP) (Bamacan) (Basement

ypoxia up-regulated 1) - Homo sapiens (Human)
RNA-binding protein 30) (RNA-binding motif protein 30) - Homo sapiens (Human)
ing transcription factor) (CCAAT-binding factor) (CBF) - Homo sapiens (Human)
(Zona occludens 2 protein) (Tight junction protein 2) - Homo sapiens (Human)
rotein sorting 29) (hVPS29) (PEP11) - Homo sapiens (Human)
ulator of chromatin subfamily C member 2 (SWI/SNF complex 170 kDa subunit) (BRG1-associated fa
rotein Bap31) (p28 Bap31) (Protein CDM) (6C6-AG tumor-associated antigen) - Homo sapiens (Hum
lpha) - Homo sapiens (Human)
ng cassette 50) (TNF-alpha-stimulated ABC protein) - Homo sapiens (Human)
mocysteine hydrolase) (AdoHcyase) - Homo sapiens (Human)

uitin-protein ligase L3) (Ubiquitin carrier protein L3) (UbcH7) (E2-F1) (L-UBC) - Homo sapiens (Huma
ripartite motif-containing protein 24) (RING finger protein 82) - Homo sapiens (Human)
omo sapiens (Human)
ve membrane anchor) (Acetylcholinesterase-associated protein) - Homo sapiens (Human)
ns (Human)
1.8.4.2) (Thioredoxin-like protein p19) (Endoplasmic reticulum protein ERp19) (ERp18) (hTLP19) - H
ulator of chromatin subfamily D member 3 (60 kDa BRG-1/Brm-associated factor subunit C) (BRG1-a
n-like 1-activating enzyme E1B) (Anthracycline-associated resistance ARX) - Homo sapiens (Human)
n-conjugating enzyme E2 M) (NEDD8 protein ligase) (NEDD8 carrier protein) - Homo sapiens (Huma
.21) (CPT I) (CPTI-L) (Carnitine palmitoyltransferase 1A) - Homo sapiens (Human)
A-binding motif protein 4a) (Lark homolog) (hLark) - Homo sapiens (Human)
proteasome regulatory subunit S10) (p42A) (Proteasome regulatory particle subunit p44S10) (Phosph
) (P1-CDC21) - Homo sapiens (Human)
1.6.1.2) (Pyridine nucleotide transhydrogenase) (Nicotinamide nucleotide transhydrogenase) - Homo
cript 2) - Homo sapiens (Human)
) - Homo sapiens (Human)

mo sapiens (Human)
nan)
ns (Human)

uron-specific 2) - Homo sapiens (Human)
) (FGAM synthase) (FGAMS) (Formylglycinamide ribotide amidotransferase) (FGARAT) (Formylglyci
oyl-CoA C-acyltransferase) (NSL-TP) (Sterol carrier protein 2) (SCP-2) (Sterol carrier protein X) (SCP
- Homo sapiens (Human)
subunit 8, mitochondrial precursor (EC 1.6.5.3) (EC 1.6.99.3) (NADH-ubiquinone oxidoreductase ASH
DAC-2) (hVDAC2) (Outer mitochondrial membrane protein porin 2) - Homo sapiens (Human)
1.-) (DEAH box protein 36) (MLE-like protein 1) (RNA helicase associated with AU-rich element ARE)
x protein UAP56) (56 kDa U2AF65-associated protein) (ATP-dependent RNA helicase p47) (HLA-B-a
e B] - Homo sapiens (Human)
C-1) (hPLIC-1) - Homo sapiens (Human)
osomal-associated 25 kDa protein) (Super protein) (SUP) - Homo sapiens (Human)
box protein 1) (DEAD box protein retinoblastoma) (DBP-RB) - Homo sapiens (Human)
elongation factor S-II protein 2) (Testis-specific S-II) (Transcription elongation factor TFIIS.l) - Homo
A (Antigen NY-CO-16) - Homo sapiens (Human)

apiens (Human)
ociated Src substrate) (CAS) (p120(cas)) - Homo sapiens (Human)
of31) - Homo sapiens (Human)
6.1.1.5) (Isoleucine--tRNA ligase) (IleRS) - Homo sapiens (Human)
.-) (ATP-dependent helicase SMARCA4) (SNF2-beta) (BRG-1 protein) (Mitotic growth and transcripti
ctivated T-cells 45 kDa) - Homo sapiens (Human)
racting protein) (S100A6-binding protein) - Homo sapiens (Human)
; Protein mago nashi homolog - Homo sapiens (Human)
n)
n)
in-interacting protein) (48 kDa TBP-interacting protein) (TIP49b) (Repressing pontin 52) (Reptin 52) (
uman)
C 2.3.1.9) (Acetoacetyl-CoA thiolase) (T2) - Homo sapiens (Human)
solic malate dehydrogenase) - Homo sapiens (Human)
g protein 2) (Nucleolar protein p40) - Homo sapiens (Human)
ein APG-2) (HSP70RY) - Homo sapiens (Human)
D box protein 39) (Nuclear RNA helicase URH49) - Homo sapiens (Human)
tin-protein ligase T) (Ubiquitin carrier protein T) - Homo sapiens (Human)
protein 4) (RBBP-4) (Retinoblastoma-binding protein p48) (Chromatin assembly factor 1 subunit C) (
factor 3-like 4) - Homo sapiens (Human)
)
) (ValRS) (Protein G7a) - Homo sapiens (Human)
ontains: BH3-interacting domain death agonist p15 (p15 BID); BH3-interacting domain death agonist
A (VAMP-associated protein A) (VAMP-A) (VAP-A) (33 kDa Vamp-associated protein) (VAP-33) - H
ens (Human)
Homo sapiens (Human)
1 (AHA1) (p38) - Homo sapiens (Human)
-muscle alpha-actinin-1) (F-actin cross-linking protein) - Homo sapiens (Human)
staglandin E2 synthase) (cPGES) (Telomerase-binding protein p23) (Hsp90 co-chaperone) (Progeste
mo sapiens (Human)
GRB2) (SH2/SH3 adapter GRB2) (Protein Ash) - Homo sapiens (Human)
syl--tRNA ligase) (TyrRS) - Homo sapiens (Human)
rotein) (Neurofilament medium polypeptide) (NF-M) (Neurofilament 3) - Homo sapiens (Human)
1.-) (DEAD box protein 23) (100 kDa U5 snRNP-specific protein) (U5-100kD) (PRP28 homolog) - Hom
trans isomerase) (PPIase) (Rotamase) (25 kDa FKBP) (FKBP-25) (Rapamycin-selective 25 kDa imm
Homo sapiens (Human)
minoimidazole-succinocarboxamide synthase (EC 6.3.2.6) (SAICAR synthetase); Phosphoribosylamin
.17) (Short chain enoyl-CoA hydratase) (SCEH) (Enoyl-CoA hydratase 1) - Homo sapiens (Human)
nscription complex subunit SSRP1) (FACT 80 kDa subunit) (FACTp80) (Chromatin-specific transcript
emia factor 2) - Homo sapiens (Human)
s (Human)

age and polyadenylation specificity factor 25 kDa subunit) (CPSF 25 kDa subunit) (Pre-mRNA cleava
in 3 - Homo sapiens (Human)
T-epsilon) - Homo sapiens (Human)
protein) - Homo sapiens (Human)
(DHP protein) - Homo sapiens (Human)
(Guanine nucleotide-binding protein subunit beta-like protein 12.3) (Receptor of activated protein kina

vating enzyme E1A) - Homo sapiens (Human)
tein kinase activator A (Protein kinase, interferon-inducible double stranded RNA-dependent activato
Human)
chain) (Alpha-II spectrin) (Fodrin alpha chain) - Homo sapiens (Human)
(Acinus) - Homo sapiens (Human)
AP113) - Homo sapiens (Human)
26S subunit ATPase 1) - Homo sapiens (Human)
helicase DHX15 (EC 3.6.1.-) (DEAH box protein 15) (ATP-dependent RNA helicase #46) - Homo sap
1-activated kinase 4) (PAK-4) - Homo sapiens (Human)
- Homo sapiens (Human)
plex 21 kDa subunit) (p21-ARC) - Homo sapiens (Human)
PIase) (Rotamase) (Cyclophilin-40) (CYP-40) (Cyclophilin-related protein) - Homo sapiens (Human)

mo sapiens (Human)
n mammary carcinoma 1 protein) (Spliceosome-associated protein SPF 27) - Homo sapiens (Human
hFXR1p) - Homo sapiens (Human)
APC-binding protein EB1) (End-binding protein 1) (EB1) - Homo sapiens (Human)
bunit beta) (Protein kinase C substrate, 60.1 kDa protein, heavy chain) (PKCSH) (80K-H protein) - Ho
DNA-directed RNA polymerase II 14.5 kDa polypeptide) (RPB9) (RPB14.5) - Homo sapiens (Human)
(Human)
sapiens (Human)

omolog (Translocase of outer membrane 40 kDa subunit homolog) (Haymaker protein) (p38.5) - Hom
38-2G4 homolog) (hG4-1) (ErbB3-binding protein 1) - Homo sapiens (Human)
scription complex subunit SPT16) (hSPT16) (FACT 140 kDa subunit) (FACTp140) (Chromatin-speci
(PHS) (4-alpha-hydroxy-tetrahydropterin dehydratase) (Phenylalanine hydroxylase-stimulating protei

bunit ATPase 4) (MIP224) (MB67-interacting protein) (TAT-binding protein 7) (TBP-7) - Homo sapien
embly protein 2) (NAP2) - Homo sapiens (Human)
yrosine kinase-associated protein 135) (BTK-associated protein 135) (BAP-135) (SRF-Phox1-interac
)
ulator of chromatin subfamily E member 1 (BRG1-associated factor 57) - Homo sapiens (Human)
fying protein 1a) (CHMP1a) (Vacuolar protein sorting 46-1) (Vps46-1) (hVps46-1) - Homo sapiens (H
ein C homolog) - Homo sapiens (Human)
l precursor (EC 1.11.1.15) (Peroxiredoxin-3) (PRX III) (Antioxidant protein 1) (AOP-1) (Protein MER5
tin homolog) (Actin-RPV) (ARP1) - Homo sapiens (Human)
S34) - Homo sapiens (Human)
anosine diphosphate dissociation inhibitor 2) (GDI-2) - Homo sapiens (Human)
P5) (Protein phosphatase T) (PP-T) (PPT) - Homo sapiens (Human)
cle myosin heavy chain IIa) (NMMHC II-a) (NMMHC-IIA) (Cellular myosin heavy chain, type A) (Nonm
endonuclease 1) (FEN-1) (Maturation factor 1) (MF1) (hFEN-1) (DNase IV) - Homo sapiens (Human)
65 kDa subunit) (U2 snRNP auxiliary factor large subunit) (hU2AF(65)) - Homo sapiens (Human)
no sapiens (Human)
mo sapiens (Human)
or (TP-beta) [Includes: 3-ketoacyl-CoA thiolase (EC 2.3.1.16) (Acetyl-CoA acyltransferase) (Beta-ket
t 2 (Progesterone membrane-binding protein) (Steroid receptor protein DG6) - Homo sapiens (Human
2) (GAP SH3 domain-binding protein 2) - Homo sapiens (Human)
snRNP-associated 110 kDa protein) (Squamous cell carcinoma antigen recognized by T cells 1) (SAR

ent DNA helicase II 70 kDa subunit) (Lupus Ku autoantigen protein p70) (Ku70) (70 kDa subunit of Ku
sphoglucose isomerase) (PGI) (Phosphohexose isomerase) (PHI) (Neuroleukin) (NLK) (Sperm antig
sor of K(+) transport growth defect 1) (Protein SKD1) - Homo sapiens (Human)
fying protein 2a) (CHMP2a) (Vacuolar protein sorting 2-1) (Vps2-1) (hVps2-1) (Putative breast adeno
ondrial precursor (EC 1.3.99.3) (MCAD) - Homo sapiens (Human)
Drebrin-F) (Cervical SH3P7) (HPK1-interacting protein of 55 kDa) (HIP-55) (Cervical mucin-associated
apiens (Human)
atin-9) (K9) - Homo sapiens (Human)
otein) (Collapsin response mediator protein 5) (CRMP-5) (CRMP3-associated molecule) (CRAM) - H
a protein 4) (DnaJ protein homolog 2) (HSJ-2) (HSDJ) - Homo sapiens (Human)
hondrial precursor (EC 1.6.5.3) (EC 1.6.99.3) (NADH-ubiquinone oxidoreductase 51 kDa subunit) (Co
MAP1 light chain LC1] - Homo sapiens (Human)
Semin-1) - Homo sapiens (Human)
otein) (hNRP) - Homo sapiens (Human)
C1alpha protein) (Sb1.8) - Homo sapiens (Human)
or (EC 1.1.1.42) (Oxalosuccinate decarboxylase) (IDH) (NADP(+)-specific ICDH) (IDP) (ICD-M) - Hor
1.-) (DEAD box protein 48) (Eukaryotic initiation factor 4A-like NUK-34) (Nuclear matrix protein 265) (
eta) (Renal carcinoma antigen NY-REN-15) - Homo sapiens (Human)
(Proliferation-inducing gene 4 protein) - Homo sapiens (Human)
1) (H2A.y) (H2A/y) (Medulloblastoma antigen MU-MB-50.205) - Homo sapiens (Human)

2.6.1.13) (Ornithine--oxo-acid aminotransferase) [Contains: Ornithine aminotransferase, hepatic form
A) (U1A protein) (U1-A) - Homo sapiens (Human)
ke 2 (GABA(A) receptor-associated protein-like 2) (Ganglioside expression factor 2) (GEF-2) (Genera
ein 114) (SAP 114) (SF3a120) - Homo sapiens (Human)
3.14) (V-ATPase B2 subunit) (Vacuolar proton pump B isoform 2) (Endomembrane proton pump 58 k
s (Human)
proteasome regulatory subunit rpn8) (26S proteasome regulatory subunit S12) (Proteasome subunit p

AP endonuclease 1) (APEX nuclease) (APEN) (REF-1 protein) - Homo sapiens (Human)

(Cytoskeleton-associated protein 1) (Cytoskeleton-associated protein CKAPI) - Homo sapiens (Hum
rotein ligase) (Ubiquitin-conjugating enzyme E2 I) (Ubiquitin-protein ligase I) (Ubiquitin carrier protein
Human)
ciated protein 1) - Homo sapiens (Human)
age and polyadenylation specificity factor 68 kDa subunit) (CPSF 68 kDa subunit) (Pre-mRNA cleava
n response mediator protein 3) (CRMP-3) (UNC33-like phosphoprotein 4) (ULIP4 protein) - Homo sap
ns (Human)
omo sapiens (Human)
an)
mt) (MRP-40) (Nuclear localization signal-containing protein deleted in velocardiofacial syndrome) (U

43) - Homo sapiens (Human)
ssociated protein p19) (p19A) (p19skp1) (RNA polymerase II elongation factor-like protein) (Organ of
(EC 3.1.1.47) (PAF acetylhydrolase 30 kDa subunit) (PAF-AH 30 kDa subunit) (PAF-AH subunit beta
nan)
inase-targeting subunit) (p50Cdc37) - Homo sapiens (Human)
CT-gamma) (hTRiC5) - Homo sapiens (Human)
ns (Human)
o sapiens (Human)
a protein 1) (Heat shock protein 40) (HSP40) (DnaJ protein homolog 1) (HDJ-1) - Homo sapiens (Hu
n response mediator protein 1) (CRMP-1) - Homo sapiens (Human)
100 co-activator) (100 kDa coactivator) (EBNA2 coactivator p100) - Homo sapiens (Human)
scle myosin heavy chain IIb) (NMMHC II-b) (NMMHC-IIB) (Cellular myosin heavy chain, type B) (Non
1) (UBF-1) (Autoantigen NOR-90) - Homo sapiens (Human)

ınit ATPase 2) (Protein MSS1) - Homo sapiens (Human)

tein I) (SP-I) [Contains: Vasostatin-1 (Vasostatin I); Vasostatin-2 (Vasostatin II); EA-92; ES-43; Pancr
ıubunit 10 (EC 1.6.5.3) (EC 1.6.99.3) (NADH-ubiquinone oxidoreductase PDSW subunit) (Complex I-
ome component C2) (Macropain subunit C2) (Multicatalytic endopeptidase complex subunit C2) (Prot

ɔ sapiens (Human)
elicase II) (Nucleolar RNA helicase Gu) (RH II/Gu) (Gu-alpha) (DEAD box protein 21) - Homo sapiens
ein) (140 kDa nucleolar phosphoprotein) (Nopp140) (Nucleolar and coiled-body phosphoprotein 1) - H
tein 25) (RNA-binding region-containing protein 7) (Protein S164) - Homo sapiens (Human)
) (Na(+)/H(+) exchange regulatory cofactor NHE-RF) (NHERF-1) (Regulatory cofactor of Na(+)/H(+) e
· Homo sapiens (Human)
nbrane protein 4) (Putative secreted protein ZSIG9) - Homo sapiens (Human)
D box protein 3, X-chromosomal) (Helicase-like protein 2) (HLP2) (DEAD box, X isoform) - Homo sap
C 2.7.11.23) (p34 protein kinase) (Cyclin-dependent kinase 1) (CDK1) - Homo sapiens (Human)

) - Homo sapiens (Human)
ɪrotein 3) - Homo sapiens (Human)

iens (Human)
L (Adenovirus early region 1B-associated protein 5) (E1B-55 kDa-associated protein 5) (E1B-AP5) - H
mt) (MRP-L41) (39S ribosomal protein L27 homolog) (MRP-L27 homolog) (BCL2-interacting mitochoı
a) (CCT-zeta-1) (Tcp20) (HTR3) (Acute morphine dependence-related protein 2) - Homo sapiens (Hu

fusion protein MSF-A) (Ovarian/Breast septin) (Ov/Br septin) (Septin D1) - Homo sapiens (Human)

a subunit) (CF-1 64 kDa subunit) (CstF-64) - Homo sapiens (Human)
2) - Homo sapiens (Human)
1.-) (DEAD box protein 17) (RNA-dependent helicase p72) (DEAD box protein p72) - Homo sapiens (

ɔE#1) (HMGE) - Homo sapiens (Human)
5.3.14) (Oligomycin sensitivity conferral protein) (OSCP) - Homo sapiens (Human)
-oxo-dGTPase) (Nucleoside diphosphate-linked moiety X motif 1) (Nudix motif 1) - Homo sapiens (Hu

mt) (MRP-L15) - Homo sapiens (Human)
e H) (Rotamase H) (U-snRNP-associated cyclophilin SnuCyp-20) (USA-CYP) (Small nuclear ribonuc
GTP-binding protein) - Homo sapiens (Human)
stamine-releasing factor) (HRF) (Fortilin) - Homo sapiens (Human)
scence-inhibited gene protein) (Protein PBK1) (CATX-11) - Homo sapiens (Human)
(EC 1.6.5.3) (EC 1.6.99.3) (NADH-ubiquinone oxidoreductase 15 kDa subunit) (Complex I-15 kDa) (C
t 1 (mPR) - Homo sapiens (Human)
ɔtein) - Homo sapiens (Human)
ke phosphoprotein) (ULIP protein) (Collapsin response mediator protein 4) (CRMP-4) - Homo sapien

C 2.6.1.1) (Transaminase A) (Glutamate oxaloacetate transaminase 2) - Homo sapiens (Human)
ɡlucosidase II subunit alpha) - Homo sapiens (Human)
1 homolog gamma) (HP1 gamma) (Modifier 2 protein) (HECH) - Homo sapiens (Human)
e maintenance protein 2 homolog) (Nuclear protein BM28) - Homo sapiens (Human)
iens (Human)
S-B) (La ribonucleoprotein) (La autoantigen) - Homo sapiens (Human)
apiens (Human)
.1.-) (G3BP-1) (ATP-dependent DNA helicase VIII) (HDH-VIII) (GAP SH3 domain-binding protein 1) -
C 5.2.1.8) (Rotamase Pin1) (PPIase Pin1) - Homo sapiens (Human)
(Human)
a) - Homo sapiens (Human)

(Human)
C 3.5.4.-) (DRADA) (136 kDa double-stranded RNA-binding protein) (P136) (K88DSRBP) (Interferon-
NA helicase II) (NDH II) (DEAH box protein 9) - Homo sapiens (Human)
artite motif-containing protein 28) (Nuclear corepressor KAP-1) (KRAB-associated protein 1) (KAP-1)
PP2C-gamma) (Protein phosphatase magnesium-dependent 1 gamma) (Protein phosphatase 1C) - H
cle myosin light chain alkali 6) (Myosin light chain alkali 3) (Myosin light chain 3) (MLC-3) (LC17) - Ho
n E) (Sm-E) (SmE) - Homo sapiens (Human)

-like protein TD-60) - Homo sapiens (Human)
L cell apoptosis-related gene 19 protein) - Homo sapiens (Human)
alactoside-binding lectin L-14-I) (Lactose-binding lectin 1) (S-Lac lectin 1) (Galaptin) (14 kDa lectin) (H
B1) (SAF-B) (HSP27 estrogen response element-TATA box-binding protein) (HSP27 ERE-TATA-bind
binding protein SNP70) (Npw38-binding protein) (NpwBP) - Homo sapiens (Human)
(LDH muscle subunit) (LDH-M) (Proliferation-inducing gene 19 protein) (Renal carcinoma antigen NY
MAGE-D) (Breast cancer-associated gene 1 protein) (BCG-1) (11B6) (Hepatocellular carcinoma-asso
p15 (SUB1 homolog) (Positive cofactor 4) (PC4) (p14) - Homo sapiens (Human)
DAC-1) (hVDAC1) (Outer mitochondrial membrane protein porin 1) (Plasmalemmal porin) (Porin 31HL
proteasome regulatory subunit p27) - Homo sapiens (Human)
actor ZFM1) (Zinc finger gene in MEN1 locus) (Mammalian branch point-binding protein mBBP) (BBF
precursor (EC 1.9.3.1) (Cytochrome c oxidase subunit IV isoform 1) (COX IV-1) (Cytochrome c oxida
uanosine diphosphate dissociation inhibitor 1) (GDI-1) (XAP-4) (Oligophrenin-2) - Homo sapiens (Hu
wth-associated protein 43) (PP46) (Neural phosphoprotein B-50) - Homo sapiens (Human)
ous nuclear ribonucleoprotein I) (hnRNP I) (57 kDa RNA-binding protein PPTB-1) - Homo sapiens (H
(Human)
s (Human)
ss I antigen-binding protein p88) (p90) (IP90) - Homo sapiens (Human)
n NY-REN-44) - Homo sapiens (Human)
ein 2 (HCV NS2 trans-regulated protein) (NS2TP) - Homo sapiens (Human)
RRM-containing coactivator activator/modulator) (Synaptotagmin-interacting protein) (SYT-interacting
4) (PABP 4) (Inducible poly(A)-binding protein) (iPABP) (Activated-platelet protein 1) (APP-1) - Homo
L49) (Protein NOF1) (Neighbor of FAU) (NOF) - Homo sapiens (Human)
in B) (TARBP-B) - Homo sapiens (Human)
1 homolog alpha) (HP1 alpha) (Antigen p25) - Homo sapiens (Human)
(Solute carrier family 25 member 3) - Homo sapiens (Human)
- Homo sapiens (Human)
rotein ERp-72) (ERp72) - Homo sapiens (Human)
2) (Poly(A)-binding protein II) (PABII) (Polyadenylate-binding nuclear protein 1) (Nuclear poly(A)-bind
ens (Human)
ens (Human)
d protein LDC2) - Homo sapiens (Human)
ome component C3) (Macropain subunit C3) (Multicatalytic endopeptidase complex subunit C3) - Ho
e I) - Homo sapiens (Human)

in (EC 1.10.2.2) (Complex III subunit VI) (QP-C) - Homo sapiens (Human)
Ribonucleoprotein RBM8A) (RNA-binding protein Y14) (Binder of OVCA1-1) (BOV-1) - Homo sapiens
)

p-binding protein) (NCBP 20 kDa subunit) (CBP20) (NCBP-interacting protein 1) (NIP1) (Cell prolifer
) (P1-CDC46) - Homo sapiens (Human)
ng protein 3 (Thyroid receptor-interacting protein 7) (TRIP7) - Homo sapiens (Human)
.1) (TFIIF-alpha) (General transcription factor IIF subunit 1) (Transcription initiation factor RAP74) (G
hondrial precursor (EC 1.9.3.1) (Cytochrome c oxidase subunit VIIa-L) (VIIaL) - Homo sapiens (Huma
A) (Template-activating factor I) (TAF-I) (HLA-DR-associated protein II) (PHAPII) (Inhibitor of granzym

s (Human)
ent RNA helicase eIF4A-1) (eIF4A-I) (eIF-4A-I) - Homo sapiens (Human)
-1) - Homo sapiens (Human)
o sapiens (Human)
neobox 2A) (Aristaless homeobox protein homolog) (ARIX1 homeodomain protein) - Homo sapiens (H
ns (Human)

(LDH heart subunit) (LDH-H) (Renal carcinoma antigen NY-REN-46) - Homo sapiens (Human)
g factor SRP75) (SRP001LB) - Homo sapiens (Human)
sociated sulphydryl protein) (SASP) - Homo sapiens (Human)
oma breakpoint region 1 protein) - Homo sapiens (Human)

(XP-C repair-complementing complex 58 kDa protein) (p58) - Homo sapiens (Human)
-associated protein 3 (Sam68-like mammalian protein 2) (SLM-2) (Sam68-like phosphotyrosine protei
ed heat-stable protein of 24 kDa) (CRHSP-24) - Homo sapiens (Human)
ding protein 1) (PQBP-1) (38 kDa nuclear protein containing a WW domain) (Npw38) - Homo sapiens
ctivated T-cells 90 kDa) (NF-AT-90) (Double-stranded RNA-binding protein 76) (DRBP76) (Translatio
e) (15S Mg(2+)-ATPase p97 subunit) (Valosin-containing protein) (VCP) - Homo sapiens (Human)
nber B (PHAPI2 protein) (Silver-stainable protein SSP29) (Acidic protein rich in leucines) - Homo sap

theta) (eIF3 p167) (eIF3 p180) (eIF3 p185) (eIF3a) - Homo sapiens (Human)
ntigen HuD) (Hu-antigen D) - Homo sapiens (Human)
Nucleolin-like protein mcs94-1) - Homo sapiens (Human)

ase (EC 3.1.3.48) (LMW-PTPase) (LMW-PTP) (Low molecular weight cytosolic acid phosphatase) (E
(Protein kinase C substrate, 80 kDa protein, light chain) (PKCSL) (80K-L protein) - Homo sapiens (H
REN-32) - Homo sapiens (Human)
cleic acid-binding protein SUB2.3) - Homo sapiens (Human)
H antigen) - Homo sapiens (Human)
mt) (MRP-L12) (5c5-2) - Homo sapiens (Human)

apiens (Human)
plication factor-A protein 3) (p14) - Homo sapiens (Human)
L27) - Homo sapiens (Human)
Homo sapiens (Human)
entrin) (Ubiquitin-like protein SMT3C) (SMT3 homolog 3) (Ubiquitin-homology domain protein PIC1) (
tein 2 homolog 1) (U4/U6.U5 tri-snRNP 15.5 kDa protein) (OTK27) (hSNU13) - Homo sapiens (Huma
(3-hydroxyacyl-CoA dehydrogenase type II) (Type II HADH) (3-hydroxy-2-methylbutyryl-CoA dehydro
ma) - Homo sapiens (Human)

(EC 3.6.3.14) (ATPase subunit F6) - Homo sapiens (Human)
(FTP-3) - Homo sapiens (Human)
cid-binding protein I) (CRABP-I) (Retinoic acid-binding protein I, cellular) - Homo sapiens (Human)
rotein disulfide isomerase P5) (Thioredoxin domain-containing protein 7) - Homo sapiens (Human)
)
(ADPRT) (NAD(+) ADP-ribosyltransferase 1) (Poly[ADP-ribose] synthetase 1) - Homo sapiens (Huma
ive SR protein LUC7B1) - Homo sapiens (Human)
o sapiens (Human)
europeptide tyrosine) (NPY); C-flanking peptide of NPY (CPON)] - Homo sapiens (Human)
Homo sapiens (Human)
otein 145) (SAP 145) (SF3b150) (Pre-mRNA-splicing factor SF3b 145 kDa subunit) - Homo sapiens (H
man)

in) (Neurofilament-66) (NF-66) - Homo sapiens (Human)

factor SF3b 10 kDa subunit) - Homo sapiens (Human)
subunit 2 (EC 1.6.5.3) (EC 1.6.99.3) (NADH-ubiquinone oxidoreductase B8 subunit) (Complex I-B8) (
ived growth factor) (Transcriptional coactivator p75/p52) (Dense fine speckles 70 kDa protein) (DFS 7

P core protein C-like 1) - Homo sapiens (Human)
Mitochondrial transcription factor 1) (MtTF1) (Transcription factor 6-like 2) - Homo sapiens (Human)
(hnRNP-Q) (Synaptotagmin-binding, cytoplasmic RNA-interacting protein) (Glycine- and tyrosine-rich

mo sapiens (Human)
nber A (Potent heat-stable protein phosphatase 2A inhibitor I1PP2A) (Acidic nuclear phosphoprotein
s (Human)
22) (NP22) - Homo sapiens (Human)
apiens (Human)
on factor 3) - Homo sapiens (Human)
or (EC 1.9.3.1) (Cytochrome c oxidase polypeptide Va) - Homo sapiens (Human)
group nucleosome-binding domain-containing protein 1) - Homo sapiens (Human)
in) (p55) - Homo sapiens (Human)
(Prx-V) (Peroxisomal antioxidant enzyme) (PLP) (Thioredoxin reductase) (Thioredoxin peroxidase PM
ns (Human)
1) (PABP 1) - Homo sapiens (Human)
n)
member 1) (94 kDa glucose-regulated protein) (GRP94) (gp96 homolog) (Tumor rejection antigen 1)
-type 2) - Homo sapiens (Human)
osin) - Homo sapiens (Human)
n response mediator protein 2) (CRMP-2) (N2A3) - Homo sapiens (Human)
12) - Homo sapiens (Human)
12) - Homo sapiens (Human)
ains: Endothelial monocyte-activating polypeptide 2 (EMAP-II) (Small inducible cytokine subfamily E m
1 homolog beta) (HP1 beta) (Modifier 1 protein) (M31) (Heterochromatin protein p25) (HP1Hsbeta) (p
3.6.3.14) - Homo sapiens (Human)
C 3.6.3.14) - Homo sapiens (Human)
0-2) - Homo sapiens (Human)
ophage myristoylated alanine-rich C kinase substrate) (Mac-MARCKS) (MacMARCKS) - Homo sapie
o sapiens (Human)
.6.3.14) - Homo sapiens (Human)
protein-bridging factor 1) (MBF1) - Homo sapiens (Human)
-associated protein) (EBER-associated protein) (EAP) (Heparin-binding protein HBp15) - Homo sapie
3) (ATP-AMP transphosphorylase) - Homo sapiens (Human)
ptor) (Colon carcinoma laminin-binding protein) (NEM/1CHD4) (Multidrug resistance-associated prote
group protein 1-like 2) (HMG-1L2) - Homo sapiens (Human)
2) (ANT 3) (ADP,ATP carrier protein 3) (Solute carrier family 25 member 6) (ADP,ATP carrier protein
g factor SRP55) - Homo sapiens (Human)

iens (Human)
ma (EC 3.1.1.47) (PAF acetylhydrolase 29 kDa subunit) (PAF-AH 29 kDa subunit) (PAF-AH subunit g
organizing protein) (Hop) (Transformation-sensitive protein IEF SSP 3521) (NY-REN-11 antigen) - Ho

n)
Pase G subunit 1) (Vacuolar proton pump G subunit 1) (V-ATPase 13 kDa subunit 1) (Vacuolar ATP s
-Cys peroxiredoxin) (1-Cys PRX) (Acidic calcium-independent phospholipase A2) (EC 3.1.1.-) (aiPLA
hormone receptor-associated protein complex 150 kDa component) (Trap150) - Homo sapiens (Hum
e protein TC4) (Androgen receptor-associated protein 24) - Homo sapiens (Human)

(Ski-interacting protein) (Nuclear receptor coactivator NCoA-62) - Homo sapiens (Human)
(Prolyl 4-hydroxylase subunit beta) (Cellular thyroid hormone-binding protein) (p55) - Homo sapiens (
ıydro-lyase) (Neural enolase) (Neuron-specific enolase) (NSE) (Enolase 2) - Homo sapiens (Human)
ein 1) (KCIP-1) (Protein 1054) - Homo sapiens (Human)

.3.1) - Homo sapiens (Human)
-cell) (HS1 protein) - Homo sapiens (Human)
Rp60) (grp60) - Homo sapiens (Human)
e-rich splicing factor-related nuclear matrix protein of 300 kDa) (Ser/Arg-related nuclear matrix protei

ant mRNA protein) (SCR10) - Homo sapiens (Human)
F) - Homo sapiens (Human)

Da Alu RNA-binding protein) - Homo sapiens (Human)
(HN1-like protein) - Homo sapiens (Human)
sapiens (Human)

tein 1) (RanBP1) - Homo sapiens (Human)

e protein 5) (Transcription elongation factor S-II protein-like 5) - Homo sapiens (Human)
ıoma antigen NY-REN-38) - Homo sapiens (Human)
ment-binding protein 107) (TAXREB107) (Neoplasm-related protein C140) - Homo sapiens (Human)
protein 9) - Homo sapiens (Human)
.-) (DEAD box protein 5) (RNA helicase p68) - Homo sapiens (Human)
-containing nuclear protein) (PCNP) - Homo sapiens (Human)
substrate (P1) - Homo sapiens (Human)

se-regulated protein) (GRP 75) (Peptide-binding protein 74) (PBP74) (Mortalin) (MOT) - Homo sapier
t Tim9 B (TIMM10B) (Tim10b) (Fracture callus protein 1) (FxC1) - Homo sapiens (Human)
subunit 7 (EC 1.6.5.3) (EC 1.6.99.3) (NADH-ubiquinone oxidoreductase subunit B14.5a) (Complex I-
Da nucleolar scleroderma antigen) - Homo sapiens (Human)
a) (HTRA2-beta) (Transformer 2 protein homolog) - Homo sapiens (Human)
protein) (HSP 27) (Stress-responsive protein 27) (SRP27) (Estrogen-regulated 24 kDa protein) (28 kD
ctor 1 A-2) (eEF1A-2) (Statin S1) - Homo sapiens (Human)
phospho-hydrolase) (PPase) - Homo sapiens (Human)
.8) (PPIase) (Rotamase) (Cyclophilin B) (S-cyclophilin) (SCYLP) (CYP-S1) - Homo sapiens (Human)

- Homo sapiens (Human)
C 5.2.1.8) (Rotamase Pin4) (PPIase Pin4) (Parvulin 14) (Par14) (Peptidyl-prolyl cis/trans isomerase E
I-B) - Homo sapiens (Human)
factor SRP40) (Delayed-early protein HRS) - Homo sapiens (Human)
t) - Homo sapiens (Human)
hain) (B-CK) - Homo sapiens (Human)

(COX VIb-1) - Homo sapiens (Human)
ctor 1 A-1) (eEF1A-1) (Elongation factor Tu) (EF-Tu) (Leukocyte receptor cluster member 7) - Homo

alpha) - Homo sapiens (Human)
) - Homo sapiens (Human)
in 1) (KCIP-1) - Homo sapiens (Human)
no sapiens (Human)

X polypeptide) - Homo sapiens (Human)
s (Human)
;in gamma) (hTM5) - Homo sapiens (Human)
/B) (APOBEC-1-binding protein 1) (ABBP-1) - Homo sapiens (Human)
Homo sapiens (Human)
-associated protein 1 (p21 Ras GTPase-activating protein-associated p62) (GAP-associated tyrosine
:rocyte differentiation-associated factor EDAF-1) (Enterocyte differentiation-promoting factor) (EDPF-
pha-tubulin) (Tubulin H2-alpha) - Homo sapiens (Human)
60) (60 kDa chaperonin) (CPN60) (Heat shock protein 60) (HSP-60) (Mitochondrial matrix protein P1)

omo sapiens (Human)
ion protein 2) (PRP 2) - Homo sapiens (Human)
tein D2) (Sm-D2) - Homo sapiens (Human)
(Human)

1) (KCIP-1) - Homo sapiens (Human)

(Human)
0 kDa) (snRNP70) (U1-70K) - Homo sapiens (Human)
35 kDa subunit) (U2 snRNP auxiliary factor small subunit) - Homo sapiens (Human)
Repressor of estrogen receptor activity) (D-prohibitin) - Homo sapiens (Human)

xonal membrane protein NAP-22) (22 kDa neuronal tissue-enriched acidic protein) - Homo sapiens (I
Homo sapiens (Human)
itin-protein ligase N) (Ubiquitin carrier protein N) (Ubc13) (Bendless-like ubiquitin-conjugating enzyme
(Thymopoietin, isoforms beta/gamma) (TP beta/gamma) (Thymopoietin-related peptide isoforms beta
(GSTP1-1) - Homo sapiens (Human)
Transformation up-regulated nuclear protein) (TUNP) - Homo sapiens (Human)
isulfide isomerase ER-60) (ERp60) (58 kDa microsomal protein) (p58) (ERp57) (58 kDa glucose-regu
(Migration-inducing gene 6 protein) - Homo sapiens (Human)

n)
3) (hnRNP 2H9) - Homo sapiens (Human)
nd B' (snRNP-B) (Sm protein B/B') (Sm-B/Sm-B') (SmB/SmB') - Homo sapiens (Human)
n protein 1) - Homo sapiens (Human)
n G) (Sm-G) (SmG) - Homo sapiens (Human)
(Human)
mo sapiens (Human)
ian)
sapiens (Human)
ndrial precursor - Homo sapiens (Human)
1.37) - Homo sapiens (Human)
Numatrin) (Nucleolar protein NO38) - Homo sapiens (Human)
uvate tautomerase) (EC 5.3.2.1) (Glycosylation-inhibiting factor) (GIF) - Homo sapiens (Human)
Human)
LS) (Translocated in liposarcoma protein) (POMp75) (75 kDa DNA-pairing protein) - Homo sapiens (
Scaffold attachment factor A) (SAF-A) (p120) (pp120) - Homo sapiens (Human)
protein HIP) - Homo sapiens (Human)
sor QM) (Laminin receptor homolog) - Homo sapiens (Human)
protein) (CRBP) - Homo sapiens (Human)
n) - Homo sapiens (Human)

ens (Human)
C 3.1.3.13) (Phosphoglycerate mutase isozyme B) (PGAM-B) (BPG-dependent PGAM 1) - Homo sap

Homo sapiens (Human)

ırotein 2) (KH type-splicing regulatory protein) (KSRP) (p75) - Homo sapiens (Human)
)) (AU-rich element RNA-binding protein 1) - Homo sapiens (Human)
NDP kinase B) (nm23-H2) (C-myc purine-binding transcription factor PUF) - Homo sapiens (Human)
s-trans isomerase) (PPIase) (Rotamase) (12 kDa FKBP) (FKBP-12) (Immunophilin FKBP12) - Homo

ı protein 56) (TAFII68) (TAF(II)68) - Homo sapiens (Human)
sapiens (Human)

AI1 RNA-binding protein 1) (PAI-RBP1) (SERPINE1 mRNA-binding protein 1) - Homo sapiens (Huma
rotein family A member 3) (snoRNP protein NOP10) - Homo sapiens (Human)
no sapiens (Human)
eat shock 70 kDa protein 5) (Immunoglobulin heavy chain-binding protein) (BiP) (Endoplasmic reticul
) (Transcriptional coactivator Aly/REF) (bZIP-enhancing factor BEF) - Homo sapiens (Human)
ciated protein with Ser-Arg repeats) (TLS-associated protein with SR repeats) (TASR) (TLS-associate
in 1) - Homo sapiens (Human)
) - Homo sapiens (Human)
-type aldolase) (Lung cancer antigen NY-LU-1) - Homo sapiens (Human)
1) (Thioredoxin-dependent peroxide reductase 1) (Thiol-specific antioxidant protein) (TSA) (PRP) (Na

ıondrial precursor (EC 3.6.1.23) (dUTPase) (dUTP pyrophosphatase) - Homo sapiens (Human)

ıman)
an)
ns-Beuren syndrome chromosome region 1 protein) - Homo sapiens (Human)
or (EC 1.9.3.1) (Cytochrome c oxidase polypeptide Vb) - Homo sapiens (Human)
n)
1) - Homo sapiens (Human)
protein 8) - Homo sapiens (Human)
tein D3) (Sm-D3) - Homo sapiens (Human)
ipase C beta 3 neighbouring gene protein) - Homo sapiens (Human)
in 4) (HMG-4) (High mobility group protein 2a) (HMG-2a) - Homo sapiens (Human)

ɪiens (Human)
Homo sapiens (Human)
man)
Prostatic-binding protein) (HCNPpp) (Neuropolypeptide h3) (Raf kinase inhibitor protein) (RKIP) [Conta
Homo sapiens (Human)
-monophosphoramidase) (Protein kinase C inhibitor 1) (Protein kinase C-interacting protein 1) (PKCI-
onO protein) (54 kDa nuclear RNA- and DNA-binding protein) (p54(nrb)) (p54nrb) (55 kDa nuclear pro

2) (Thioredoxin-dependent peroxide reductase 2) (Proliferation-associated gene protein) (PAG) (Natu
e kinase muscle isozyme) (Pyruvate kinase 2/3) (Cytosolic thyroid hormone-binding protein) (CTHBP
rotein 1) (FBP) (DNA helicase V) (HDH V) - Homo sapiens (Human)
in 3) (RNPL) - Homo sapiens (Human)
ı)

ie tract-binding protein-associated-splicing factor) (PTB-associated-splicing factor) (PSF) (DNA-bindir
sociated protein) (PAP) (PDGFA-associated protein 1) (PAP1) - Homo sapiens (Human)
nding protein CIRP) (A18 hnRNP) - Homo sapiens (Human)
ursor (Mt-SSB) (MtSSB) (PWP1-interacting protein 17) - Homo sapiens (Human)
12) (GAPDH) - Homo sapiens (Human)

ding protein 1) (Y-box transcription factor) (YB-1) (CCAAT-binding transcription factor I subunit A) (CE

ibitor) (DBI) (Endozepine) (EP) - Homo sapiens (Human)
s (Human)
g factor SRP20) - Homo sapiens (Human)

g factor SRp30C) - Homo sapiens (Human)
P C1 / hnRNP C2) - Homo sapiens (Human)
35) (SC-35) (Splicing component, 35 kDa) (Protein PR264) - Homo sapiens (Human)
n)

NDP kinase A) (Tumor metastatic process-associated protein) (Metastasis inhibition factor nm23) (nn
4.19.12) (EC 6.-.-.-) (UCH-L1) (Ubiquitin thioesterase L1) (Neuron cytoplasmic protein 9.5) (PGP 9.5)
F-5A1) (Eukaryotic initiation factor 5A isoform 1) (eIF-5A) (eIF-4D) (Rev-binding factor) - Homo sapien

dro-lyase) (Non-neural enolase) (NNE) (Enolase 1) (Phosphopyruvate hydratase) (C-myc promoter-bi

abilizing protein) (Single-strand RNA-binding protein) (hnRNP core protein A1) - Homo sapiens (Hum
in 1) (HMG-1) - Homo sapiens (Human)
3) - Homo sapiens (Human)
factor SF2, P33 subunit) (Alternative-splicing factor 1) (ASF-1) - Homo sapiens (Human)

hosphate isomerase) - Homo sapiens (Human)

High mobility group AT-hook protein 1) (High mobility group protein A1) (High mobility group protein-R
(CFA) (TCP1-chaperonin cofactor A) - Homo sapiens (Human)
Ubiquitin-like protein SMT3B) (SMT3 homolog 2) (Sentrin-2) (HSMT3) (SUMO-3) - Homo sapiens (Hu
P A2 / hnRNP B1) - Homo sapiens (Human)

a chaperonin) (CPN10) (Early-pregnancy factor) (EPF) - Homo sapiens (Human)
(RNA-binding motif protein, X chromosome) (Glycoprotein p43) - Homo sapiens (Human)

c system regulatory peptide (Seraspenide)] - Homo sapiens (Human)
in 2) (HMG-2) - Homo sapiens (Human)

rotein) (p18) - Homo sapiens (Human)
piens (Human)
piens (Human)
uman)

Human)
ens (Human)

group nucleosome-binding domain-containing protein 2) - Homo sapiens (Human)
(Op18) (Leukemia-associated phosphoprotein p18) (pp17) (Prosolin) (Metablastin) (Protein Pr22) - H

e A) (Rotamase A) (Cyclophilin A) (Cyclosporin A-binding protein) - Homo sapiens (Human)

:or HA1/AP1 adaptin beta subunit) (Clathrin assembly protein complex 1 beta large chain) - Homo sa|

ınd form; NADH-cytochrome b5 reductase soluble form] - Homo sapiens (Human)
ıophilin FKBP65) - Homo sapiens (Human)

rotein chain B] - Homo sapiens (Human)

ıg protein complex 150 kDa component) (DRIP150) (Thyroid hormone receptor-associated protein cc
piens (Human)

:N-14) - Homo sapiens (Human)

sapiens (Human)

o sapiens (Human)
· Homo sapiens (Human)

9) (Mitogen-activated protein kinase 8-interacting protein 4) (Human lung cancer protein 6) (HLC-6)
84) (Glutathione-dependent formaldehyde dehydrogenase) (FDH) - Homo sapiens (Human)

: organic anion transporter-D) (MOAT-D) - Homo sapiens (Human)

; 3-oxoacyl-[acyl-carrier-protein] synthase (EC 2.3.1.41); 3-oxoacyl-[acyl-carrier-protein] reductase (E

omatin subfamily F member 1) (SWI-SNF complex protein p270) (B120) (SWI-like protein) (Osa homo
tein P34H) - Homo sapiens (Human)

(AKAP 350) (hgAKAP 350) (AKAP 120-like protein) (Protein hyperion) (Protein yotiao) (Centrosome-
ı partner of MLL) - Homo sapiens (Human)
s (Human)
Human)

oA dehydrogenase (EC 1.1.1.35)] - Homo sapiens (Human)

m protein) (Dystonin) - Homo sapiens (Human)
Homo sapiens (Human)

n NUANCE) - Homo sapiens (Human)
etase) (Phosphoribosylglycinamide synthetase); Phosphoribosylformylglycinamidine cyclo-ligase (EC

ens (Human)

man)

Human)

otonyl-CoA:carbon dioxide ligase subunit beta) (3-Methylcrotonyl-CoA carboxylase non-biotin-contain

) (ABP620) - Homo sapiens (Human)

3.5.2.3)] - Homo sapiens (Human)

omo sapiens (Human)

-x) (Reticulon-5) - Homo sapiens (Human)

piens (Human)

otease OTUB1) (Deubiquitinating enzyme OTUB1) - Homo sapiens (Human)

piens (Human

opp34) - Homo sapiens (Human)

P-278) (Filamin 3) (Filamin homolog 1) (Fh1) - Homo sapiens (Human)

19 protein) (PP1-binding protein of 114 kDa) (p99) - Homo sapiens (Human)
ing protein) (Placental protein 17) (PP17) - Homo sapiens (Human)

tner A) (Fas ligand-associated factor 1) (NY-REN-6 antigen) - Homo sapiens (Human)
)a subunit) (DNA-binding protein PO-GA) - Homo sapiens (Human)

ı)
Human)

or 1) (AF) (ASF) - Homo sapiens (Human)

(Protein-tyrosine kinase byk) - Homo sapiens (Human)
sapiens (Human)

Homo sapiens (Human)
emoglobin subunit gamma-1 (Hemoglobin gamma-1 chain) (Gamma-1-globin) (Hemoglobin gamma-A

boxamide ribonucleotide formyltransferase) (AICAR transformylase); IMP cyclohydrolase (EC 3.5.4.1
oxal lyase) - Homo sapiens (Human

nan)

yclohydrolase (EC 3.5.4.9); Formyltetrahydrofolate synthetase (EC 6.3.4.3)] - Homo sapiens (Human

H homolog) - Homo sapiens (Human)
unit p112) - Homo sapiens (Human)

) (CI-B18) (Cell adhesion protein SQM1) - Homo sapiens (Human)

Human)
- Homo sapiens (Human)

scular anticoagulant-alpha) (VAC-alpha) (Anchorin CII) - Homo sapiens (Human)

ı)

uman)

15S2 protein) - Homo sapiens (Human)

ge factor Im 59 kDa subunit) - Homo sapiens (Human)
ns (Human)

ınit p97) (Tumor necrosis factor type 1 receptor-associated protein 2) (55.11 protein) - Homo sapiens
ctokinase-M) - Homo sapiens (Human)

ıs (Human)

36 kDa subunit of Ku antigen) (Thyroid-lupus autoantigen) (TLAA) (CTC box-binding factor 85 kDa su

(Human)

in 2) (hSNU114) - Homo sapiens (Human)
(Human)

(Golgi adaptor HA1/AP1 adaptin sigma-1A subunit) (Clathrin assembly protein complex 1 sigma-1A s

nase, platelet type) - Homo sapiens (Human)

- Homo sapiens (Human)

Human)

subunit 3-like 1) (BRR2 homolog) - Homo sapiens (Human)

) sapiens (Human)

ns (Human)

uctase) - Homo sapiens (Human)
aining protein 3) - Homo sapiens (Human)

ARS-cyclophilin) (CARS-Cyp) (SR-cyclophilin) (SRcyp) (SR-cyp) (CASP10) - Homo sapiens (Human)
1) - Homo sapiens (Human)
(Human)

) - Homo sapiens (Human)

lta) - Homo sapiens (Human)

Homo sapiens (Human)

.17); Long chain 3-hydroxyacyl-CoA dehydrogenase (EC 1.1.1.211)] - Homo sapiens (Human)

0-RhoGAP) - Homo sapiens (Human)

otein of 57 kDa) (Nucleolar protein NAP57) (CBF5 homolog) - Homo sapiens (Human)
tein) (52 kDa FK506-binding protein) (FKBP59) - Homo sapiens (Human)

n)

otidase T) - Homo sapiens (Human)

CAP1) (PBR- and PKA-associated protein 7) (Peripheral benzodiazepine receptor-associated protein
nWALP2) - Homo sapiens (Human)

(Proline--tRNA ligase)] - Homo sapiens (Human)
hannel subunit alpha KvLQT1) (KQT-like 1) - Homo sapiens (Human)
ulin 1) (PHAS-I) - Homo sapiens (Human)
Human)
egulator complex subunit alpha) (REG-alpha) (Interferon gamma up-regulated I-5111 protein) (IGUP I
iption initiation factor RAP30) - Homo sapiens (Human)
: membrane-associated chondroitin proteoglycan) - Homo sapiens (Human)

actor 170) - Homo sapiens (Human)
an)

an)

Homo sapiens (Human)
associated factor 60C) - Homo sapiens (Human)
)
an)

monoformate immuno-associated protein 4) (Breast cancer-associated protein SGA-113M) - Homo sa

sapiens (Human)

inamide ribotide synthetase) - Homo sapiens (Human)
P-X) (SCP-chi) (SCPX) - Homo sapiens (Human)

HI subunit) (Complex I-ASHI) (CI-ASHI) - Homo sapiens (Human)

) - Homo sapiens (Human)
associated transcript-1) - Homo sapiens (Human)

sapiens (Human)

ion activator) (Brahma protein homolog 1) (SWI/SNF-related matrix-associated actin-dependent regul

(51 kDa erythrocyte cytosolic protein) (ECP-51) (TIP60-associated protein 54-beta) (TAP54-beta) - H

(CAF-1 subunit C) (Chromatin assembly factor I p48 subunit) (CAF-I 48 kDa subunit) (CAF-I p48) (Nu

p13 (p13 BID); BH3-interacting domain death agonist p11 (p11 BID)] - Homo sapiens (Human)
omo sapiens (Human)

erone receptor complex p23) - Homo sapiens (Human)

mo sapiens (Human)
nunophilin) - Homo sapiens (Human)

noimidazole carboxylase (EC 4.1.1.21) (AIR carboxylase) (AIRC)] - Homo sapiens (Human)

tion elongation factor 80 kDa subunit) (Structure-specific recognition protein 1) (hSSRP1) (Recombina

ge factor Im 25 kDa subunit) (Nucleoside diphosphate-linked moiety X motif 21) (Nudix motif 21) - Ho

ase C 1) (RACK1) (Receptor for activated C kinase) - Homo sapiens (Human)

or) (Protein activator of the interferon-induced protein kinase) (PKR-associated protein X) (PKR-assoc

piens (Human)

)

omo sapiens (Human)
)

no sapiens (Human)

fic transcription elongation factor 140 kDa subunit) - Homo sapiens (Human)
n) (Pterin carbinolamine dehydratase) (PCD) (Dimerization cofactor of hepatocyte nuclear factor 1-al

s (Human)

ting protein) (SPIN) (Williams-Beuren syndrome chromosome region 6 protein) - Homo sapiens (Hum

uman)

5 homolog) (HBC189) - Homo sapiens (Human)

nuscle myosin heavy chain-A) (NMMHC-A) - Homo sapiens (Human)
)

othiolase)] - Homo sapiens (Human)
n)

RT-1) (hSART-1) (hSnu66) - Homo sapiens (Human)

ı antigen) (Thyroid-lupus autoantigen) (TLAA) (CTC box-binding factor 75 kDa subunit) (CTCBF) (CT
en 36) (SA-36) - Homo sapiens (Human)

ocarcinoma marker BC-2) - Homo sapiens (Human)

d protein) - Homo sapiens (Human)

omo sapiens (Human)

omplex I-51kD) (CI-51kD) (NADH dehydrogenase flavoprotein 1) - Homo sapiens (Human)

mo sapiens (Human)
(hNMP 265) (Eukaryotic translation initiation factor 4A isoform 3) - Homo sapiens (Human)

n; Ornithine aminotransferase, renal form] - Homo sapiens (Human)

al protein transport factor p16) (MAP1 light chain 3-related protein) - Homo sapiens (Human)

kDa subunit) (HO57) - Homo sapiens (Human)

p40) (Mov34 protein homolog) - Homo sapiens (Human)

ıan)
ı I) (Ubiquitin carrier protein 9) (p18) - Homo sapiens (Human)

ge factor Im 68 kDa subunit) (Protein HPBRII-4/7) - Homo sapiens (Human)
oiens (Human)

p-regulated in metastasis) - Homo sapiens (Human)

Corti protein 2) (OCP-II protein) (OCP-2) (Transcription elongation factor B) (SIII) - Homo sapiens (H
a) (PAFAH subunit beta) - Homo sapiens (Human)

man)

ımuscle myosin heavy chain-B) (NMMHC-B) - Homo sapiens (Human)

reastatin; SS-18; WA-8; WE-14; LF-19; AL-11; GV-19; GR-44; ER-37] - Homo sapiens (Human)
PDSW) (CI-PDSW) - Homo sapiens (Human)
teasome nu chain) (30 kDa prosomal protein) (PROS-30) - Homo sapiens (Human)

s (Human)
Homo sapiens (Human)

exchanger) (Sodium-hydrogen exchanger regulatory factor 1) (Solute carrier family 9 isoform 3 regula

piens (Human)

Homo sapiens (Human)
ndrial ribosomal protein L41) (Proliferation-inducing gene 3 protein) - Homo sapiens (Human)
uman)

(Human)

uman)

cleoprotein particle-specific cyclophilin H) (CypH) - Homo sapiens (Human)

CI-15 kDa) - Homo sapiens (Human)

ns (Human)

Homo sapiens (Human)

inducible protein 4) (IFI-4 protein) - Homo sapiens (Human)

(KRAB-interacting protein 1) (KRIP-1) (RING finger protein 96) - Homo sapiens (Human)
lomo sapiens (Human)
mo sapiens (Human)

HPL) (HBL) (Putative MAPK-activating protein MP12) - Homo sapiens (Human)
ding protein) - Homo sapiens (Human)

'-REN-59) - Homo sapiens (Human)
ciated protein JCL-1) - Homo sapiens (Human)

-) (Porin 31HM) - Homo sapiens (Human)

') - Homo sapiens (Human)
ase polypeptide IV) - Homo sapiens (Human)
ıman)

luman)

protein) - Homo sapiens (Human)
sapiens (Human)

ling protein 1) - Homo sapiens (Human

mo sapiens (Human)

s (Human)

ation-inducing gene 55 protein) - Homo sapiens (Human)

ieneral transcription factor IIF polypeptide 1 74 kDa subunit protein) - Homo sapiens (Human)
an)
ne A-activated DNase) (IGAAD) - Homo sapiens (Human)

ɨuman)

in) (RNA-binding protein T-Star) - Homo sapiens (Human)

; (Human)
onal control protein 80) (TCP80) (Nuclear factor associated with dsRNA) (NFAR) (M-phase phosphop

piens (Human)

:C 3.1.3.2) (Red cell acid phosphatase 1) (Adipocyte acid phosphatase) - Homo sapiens (Human)
ɨuman)

(Ubiquitin-like protein UBL1) (GAP-modifying protein 1) (GMP1) - Homo sapiens (Human)
ın)
ɔgenase) (EC 1.1.1.178) (Endoplasmic reticulum-associated amyloid beta-peptide-binding protein) (S

an)

Human)

(CI-B8) - Homo sapiens (Human)
70) (CLL-associated antigen KW-7) - Homo sapiens (Human)

RNA-binding protein) (GRY-RBP) (NS1-associated protein 1) - Homo sapiens (Human)

pp32) (Leucine-rich acidic nuclear protein) (Lanp) (Putative HLA-DR-associated protein I) (PHAPI) (M

MP20) (Antioxidant enzyme B166) (AOEB166) (TPx type VI) (Liver tissue 2D-page spot 71B) (Alu cor

- Homo sapiens (Human)

member 1)] - Homo sapiens (Human)
p25beta) - Homo sapiens (Human)

ens (Human)

ens (Human)

ein MGr1-Ag) - Homo sapiens (Human)

n, isoform T2) - Homo sapiens (Human)

gamma) (PAFAH subunit gamma) - Homo sapiens (Human)
mo sapiens (Human)

synthase subunit M16) - Homo sapiens (Human)
A2) (Non-selenium glutathione peroxidase) (EC 1.11.1.7) (NSGPx) (24 kDa protein) (Liver 2D page sp
an)

(Human)

n) (SR-related nuclear matrix protein of 300 kDa) (Splicing coactivator subunit SRm300) (300 kDa nu

ns (Human)

-B14.5a) (CI-B14.5a) - Homo sapiens (Human)

Da heat shock protein) - Homo sapiens (Human)

EPVH) (hPar14) - Homo sapiens (Human)

sapiens (Human)

phosphoprotein p62) (Src-associated in mitosis 68 kDa protein) (Sam68) (p68) - Homo sapiens (Hum
1) (Vitamin D3-inducible protein) (DDVit 1) - Homo sapiens (Human)

) (P60 lymphocyte protein) (HuCHA60) - Homo sapiens (Human)

Human)

e) - Homo sapiens (Human)
a/gamma) (TPRP isoforms beta/gamma) [Contains: Thymopoietin (TP) (Splenin); Thymopentin (TP5)

ulated protein) - Homo sapiens (Human)

(Human)

piens (Human)

sapiens (Human)

an)

lum lumenal Ca(2+)-binding protein grp78) - Homo sapiens (Human)

ed serine-arginine protein) (TLS-associated SR protein) (40 kDa SR-repressor protein) (SRrp40) (Spli

atural killer cell-enhancing factor B) (NKEF-B) - Homo sapiens (Human)

ains: Hippocampal cholinergic neurostimulating peptide (HCNP)] - Homo sapiens (Human)

-1) - Homo sapiens (Human)
otein) (NMT55) (DNA-binding p52/p100 complex, 52 kDa subunit) - Homo sapiens (Human)

ural killer cell-enhancing factor A) (NKEF-A) - Homo sapiens (Human)
P) (THBP1) - Homo sapiens (Human)

ng p52/p100 complex, 100 kDa subunit) (100 kDa DNA-pairing protein) (hPOMp100) - Homo sapiens

3F-A) (Enhancer factor I subunit A) (EFI-A) (DNA-binding protein B) (DBPB) - Homo sapiens (Human

n23-H1) (Granzyme A-activated DNase) (GAAD) - Homo sapiens (Human)
(PGP9.5) - Homo sapiens (Human)
ns (Human)

inding protein) (MBP-1) (MPB-1) (Plasminogen-binding protein) - Homo sapiens (Human)

an)

) - Homo sapiens (Human)

man)

lomo sapiens (Human)

piens (Human)

omplex 170 kDa component) (Trap170) (Activator-recruited cofactor 150 kDa component) (ARC150) -

(Proliferation-inducing protein 6) (Sperm-specific protein) (Sperm surface protein) (Protein highly exp

C 1.1.1.100); 3-hydroxypalmitoyl-[acyl-carrier-protein] dehydratase (EC 4.2.1.61); Enoyl-[acyl-carrier-

olog 1) (hOSA1) (hELD) (BRG1-associated factor 250) (BAF250) (BRG1-associated factor 250a) (BA

- and Golgi-localized PKN-associated protein) (CG-NAP) - Homo sapiens (Human)

: 6.3.3.1) (AIRS) (Phosphoribosyl-aminoimidazole synthetase) (AIR synthase); Phosphoribosylglycina

ning subunit) - Homo sapiens (Human

A chain) (Hb F Agamma) - Homo sapiens (Human); Hemoglobin subunit epsilon (Hemoglobin epsilon

10) (Inosinicase) (IMP synthetase) (IMP cyclohydrolase) (ATIC)] - Homo sapiens (Human)

s (Human)

ıbunit) (CTCBF) (CTC85) (Nuclear factor IV) (DNA-repair protein XRCC5) - Homo sapiens (Human)

small chain) (Clathrin coat assembly protein AP19) (HA1 19 kDa subunit) (Sigma 1a subunit of AP-1

ı PAP7) - Homo sapiens (Human)

l-5111) - Homo sapiens (Human)

ıpiens (Human)

lator of chromatin subfamily A member 4) - Homo sapiens (Human)

omo sapiens (Human)

ıcleosome remodeling factor subunit RBAP48) - Homo sapiens (Human)

ation signal sequence recognition protein 1) (T160) - Homo sapiens (Human)

omo sapiens (Human)

ciating protein X) - Homo sapiens (Human)

pha) (Dimerization cofactor of HNF1) (DCoH) - Homo sapiens (Human)

nan)

C75) (DNA-repair protein XRCC6) - Homo sapiens (Human)

Human)

atory factor 1) - Homo sapiens (Human)

rotein 4) (MPP4) - Homo sapiens (Human)

Short-chain type dehydrogenase/reductase XH98G2) - Homo sapiens (Humar

lapmodulin) - Homo sapiens (Human)

epressor 1) - Homo sapiens (Human)

ot 40) (Red blood cells page spot 12) - Homo sapiens (Human)

uclear matrix antigen) - Homo sapiens (Human)

nan)

] - Homo sapiens (Human)

icing factor SRp38) - Homo sapiens (Human)

; (Human)

[illegible])

- Homo sapiens (Human)

ɔressed in testis) (PHET) (Sunday driver 1) - Homo sapiens (Human)

-protein] reductase (EC 1.3.1.10); Oleoyl-[acyl-carrier-protein] hydrolase (EC 3.1.2.14)] - Homo sapie

\F250A) - Homo sapiens (Human)

amide formyltransferase (EC 2.1.2.2) (GART) (GAR transformylase) (5'-phosphoribosylglycinamide tr

chain) (Epsilon-globin) - Homo sapiens (Human)

clathrin) - Homo sapiens (Human)

ens (Humar

ransformylase)] - Homo sapiens (Human
